# Simvastatin reduces the carcinogenic effect of 3-methylcholanthrene in renal epithelial cells through histone deacetylase 1 inhibition and RhoA reactivation

**DOI:** 10.1038/s41598-019-40757-6

**Published:** 2019-03-14

**Authors:** Chih-Cheng Chang, Kuo-How Huang, Sung-Po Hsu, Yuan-Chii G. Lee, Yuh-Mou Sue, Shu-Hui Juan

**Affiliations:** 1 Department of Physiology, School of Medicine, College of Medicine, Taipei Medical University, Taipei, Taiwan; 2 National Taiwan University Hospital; Department of Urology, College of Medicine, National Taiwan University; and National Taiwan University Hospital, Taipei, Taiwan; 3 Graduate Institute of Biomedical Informatics, College of Medical Science and Technology, Taipei Medical University, Taipei, Taiwan; 4 Division of Nephrology, Department of Internal Medicine, School of Medicine, College of Medicine and Division of Nephrology, Department of Internal Medicine, Wan Fang Hospital, Taipei Medical University, Taipei, Taiwan

## Abstract

The therapeutic effects of simvastatin for renal cell carcinoma (RCC) are controversial. In this study, the effects of simvastatin on the carcinogenic properties of 3-methylcholanthrene (3MC; an aryl-hydrocarbon receptor [AhR] agonist) in human renal epithelial cells (hRECs) were investigated. We exposed *in vitro* and *in vivo* models to 3MC to induce RCC onset. 3MC upregulated the epithelial–mesenchymal transition (EMT) and tumor biomarkers; the models exhibited the reciprocal expression of histone deacetylase 1 (HDAC1) and RhoA, namely increased HDAC1 and decreased RhoA expression, through hypoxia-inducible-factor (HIF)- and AhR-dependent mechanisms. In addition to inducing EMT biomarkers, 3MC decreased von Hippel–Lindau protein levels (a risk factor for RCC) and increased CD44 expression in hRECs, which were reversed by digoxin (a HIF inhibitor) and HDAC inhibitors (suberoylanilide hydroxamic acid and trichostatin A [TSA]). Simvastatin abolished the detrimental effects of 3MC by reducing HDAC1 expression, with resulting RhoA upregulation, and reactivating RhoA *in vitro* and *in vivo*. Notably, the protective effects of simvastatin were negated by an HDAC activator (ITSA) through TSA suppression. The crucial role of RhoA in RCC carcinogenesis was verified by the overexpression of constitutively active RhoA. Collectively, these results demonstrate that simvastatin restores RhoA function through HDAC1 inhibition; therefore, simvastatin might serve as adjunct therapy for RCC induced by 3MC.

## Introduction

Renal cell carcinoma (RCC) accounts for approximately 3% of adult malignancies and more than 85% of renal cancers. The most prevalent subtype is clear cell (cc) RCC, which contributes to 75–80% of the RCC incidence and mainly results from the loss of function of the von Hippel–Lindau (VHL) gene^1^. The VHL protein (pVHL) functions as a tumor suppressor and is responsible for the ubiquitination and proteasome degradation^1,2^ of hypoxia-inducible factors (HIFs). HIFα is a key regulator of the hypoxic response, a primary target of the pVHL. In hypoxia or when the pVHL functions abnormally, the pVHL fails to ubiquitinate HIFα. HIFα then accumulates, thereby activating the transcription of hypoxia-inducible genes and subsequently increasing tumor aggressiveness. Patients with RCC are usually resistant to chemotherapy, and only a small percentage of patients with RCC benefit from cytokine treatment^3^. Thus, nephrectomy is common and effective treatment for such patients. Although the prognosis of RCC is mainly related to the clinical tumor stages, elucidation of its underlying mechanisms may enable the development of new therapeutic strategies. Sorafenib tosylate (Nexavar; Onyx Pharmaceuticals, Emeryville, CA, USA) is an orally active inhibitor of multiple kinases, including vascular endothelial growth factor (VEGF) receptors 2 and 3 and platelet-derived growth factor β receptor. In a phase III treatment approaches in renal cancer global evaluation trial, the efficacy and tolerability of sorafenib were demonstrated in 903 patients with advanced RCC^4,5^.

The activation of Rho GTPases (e.g., RhoA) produces structural changes in the plasma membrane that are associated with actin filament bundling and actomyosin-based cytoskeletal function^6^. The effects of altered RhoA expression on tumor progression depend on the cell type; conflicting results regarding these effects have been reported^7^. One study indicated that the downregulation of RhoA plays a crucial role in enabling RCC cells to escape apoptosis and enhancing RCC migration^8^. Recent studies have also demonstrated that RhoA downregulation is critical for enhancing the migration and invasion of pancreatic carcinoma cells and the tumorigenesis and migration of conventional RCC^8,9^. However, upregulated RhoA expression is associated with tumor progression in ovarian, gastric, and testicular cancer cells^10–12^.

Statins (e.g., simvastatin and lovastatin) inhibit 3-hydroxy-3-methylglutaryl-coenzyme A and are used clinically as lipid-lowering medications. Studies have indicated that simvastatin induces apoptosis in human colorectal cancer cells by activating RhoA and Rac1 GTPase^13^. Additionally, molecular modeling suggests that statins in acid form, which possess a carboxylic-acid-containing long chain, exhibit similar structures to those of trichostatin A (TSA) and suberoylanilide hydroxamic acid (SAHA), and that they chelate the catalytic site of human histone deacetylase 2 (HDAC2)^14^. These properties enable statins to inhibit HDAC activity and increase the hyperacetylation of histone H3 in human lung cancer cells^14^. Increasing evidence indicates that statins can inhibit tumor growth and induce apoptosis in various tumor cell lines, including glioma, neuroblastoma, melanoma, and leukemia cell lines^15,16^. Moreover, statin use in targeted therapy is associated with an increased survival rate among patients with metastatic RCC^17^ and a reduced risk of progression and reduced overall mortality after surgery for localized RCC^18^, although cohort studies have reported conflicting results regarding the therapeutic effects of statins for RCC^19^. A recent systematic review and meta-analysis demonstrated that statin administration is associated with significantly improved cancer-specific and overall survival among patients with kidney cancer^20^.

Dioxin and other aryl-hydrocarbon receptor (AhR) agonists (e.g., benzo(a)pyrene and 3-methylcholanthrene [3MC]) can enhance the progression of various cancers, including liver, skin, lung, and renal cancers^21–23^. However, whether these AhR agonists exhibit tumor-initiating activity remains unclear. Upon ligation with dioxin-related chemicals, activated AhR translocates to the nuclei along with AhR nuclear translocator (ARNT) to trigger the transcription of numerous genes, including those in the cytochrome P- (CYP) 450 family. However, the effect of AhR activation on cancer cells remains controversial. For example, in a study in which AhR target genes (e.g., *CYP1A1* and *CYP1B1*) were employed to determine AhR activation in human tumor samples, increased nuclear AhR levels were associated with poor prognosis in patients with lung squamous cell carcinoma^24^. By contrast, another study revealed that the AhR-mediated upregulation of miRNA-212/132 suppressed metastasis in breast cancer by targeting the prometastic protein SRY-related HMG-box 4^25^. Similarly, after diethylnitrosamine (carcinogen) treatment, AhR knockout mice developed significantly more liver adenomas and exhibited higher expression of proliferative and inflammatory markers than did AhR wild-type mice^26^. To resolve the inconsistencies in the aforementioned findings, we investigated the effect of 3MC on the onset and progression of RCC using *in vitro* and *in vivo* models of human renal epithelial cells (hRECs) and human RCC cells.

In this study, we aimed to reveal the mechanisms underlying the tumor-promoting effects of 3MC in RECs, with a particular focus on HIF1α/HDAC1 and RhoA, and to determine whether simvastatin can prevent these effects. Information regarding these underlying mechanisms may serve as a reference in the development of therapeutic interventions for RCC involving RhoA activators and HDAC inhibitors.

## Results

### 3MC negatively affected hRECs through HIF1α-mediated HDAC1 upregulation

**Figure 1:**
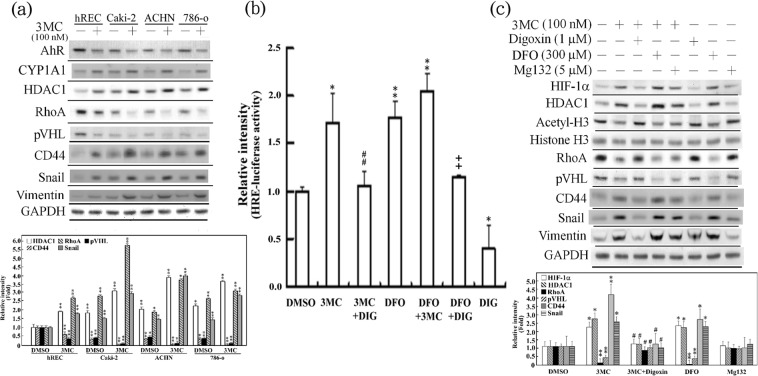
Effect of AhR-ligand exposure on EMT and RCC malignancy. Adverse effect of 3MC in hRECs, Caki-2 and other renal cell carcinoma cells (**a**) was assessed through EMT markers and hypoxia-associated proteins in Western blot analysis. Cyp1A1, a downstream of AhR, was used as a positive control for 3MC’s action, and GAPDH was used to verify equivalent loading. The data are representative of the results of three independent experiments, and the data are presented as the mean ± SD (*P < 0.05 and **P < 0.01 vs. hRECs). (**b**) hRECs were transfected with pGL2/3HRE overnight, followed by pretreatment with digoxin (a HIF inhibitor) for 24 h and deferoxamine (DFO; a HIF inducer) for 4 h prior to a 2-h 3MC challenge. The data are presented as the mean ± SD (n = 4; *P < 0.05 and **P < 0.01 vs. DMSO; ^##^P < 0.01 vs. DFO). (**c**) The adverse effect of 3MC in hRECs was assessed using digoxin, DFO, and Mg132 (**a** proteasome inhibitor). Cells that underwent similar chemical interventions to those described previously were treated with 3MC for 3 h. In the resulting cell lysates, the molecules involved in EMT or carcinogenesis and epigenetic modification were analyzed as indicated. The bar charts and Table S1 show the band intensities of the indicated proteins normalized using densitometry with GAPDH. The data are representative of the results of three independent experiments, and the data are presented as the mean ± SD (*P < 0.05 and **P < 0.01 vs. control; ^#^P < 0.05 and ^##^P < 0.01 vs. 3MC treatment alone). The gels have been run in the same experimental conditions and the cropped blots were shown. The entire gel pictures were shown in the Supplemental Fig. 1.

To examine the adverse effects of 3MC in renal cells, various renal cells were exposed to 3MC, and their epithelial–mesenchymal transition (EMT) and RCC biomarkers were analyzed using Western blotting. The results in Fig. 1a indicated that 3MC treatment altered the levels of proteins potentially involved in RCC onset and progression. Specifically, levels of the pVHL and RhoA were decreased, and the expression of HDAC1, CD44 (a cancer stem cell [CSC] marker), Snail, and vimentin (EMT markers) in normal hRECs and various renal tumor cell types (Caki-2, ACHN, and 798-o) was upregulated. hRECs treated with 3MC were used as a model for exploring the mechanisms underlying RCC onset. In addition to using 3MC as an AhR activator, benzo(a)pyrene, a widespread environmental contaminant, was employed to validate the effect of AhR in RCC. The effects of benzo(a)pyrene were similar to those of 3MC; it induced RCC molecular phenotypes in hRECs and increased RCC progression by upregulating Snail, vimentin and CD44, as depicted in Supplementary Fig. S1.

One potential etiological factor of RCC is the activation of hypoxia signaling due to loss of pVHL function, resulting in HIFα stability. As shown in Fig. 1b, the HRE-driven luciferase assay indicated that 3MC increased HIF transactivational activity in hRECs, and this activity was enhanced by deferoxamine (DFO; a HIF inducer) but inhibited by digoxin (a HIF inhibitor). We examined the detrimental effect of 3MC on hypoxic signaling in hRECs. The results presented in Fig. 1c demonstrate that, similar to the hypoxic effects of DFO, 3MC enhanced RCC molecular phenotypes in hRECs. Specifically, 3MC increased HIF1α, HDAC1, CD44, Snail and vimentin levels and decreased acetyl-histone H3, RhoA, and pVHL levels. Digoxin reversed these effects of 3MC in hRECs. In addition, MG132, a proteasome inhibitor, was employed to examine whether the proteasome degradation of downregulated RhoA protein occurs. However, no restoration was apparent.

### Similar to HDAC inhibitors, simvastatin restored RhoA function in 3MC-treated hRECs through HDAC1 inhibition

**Figure 2:**
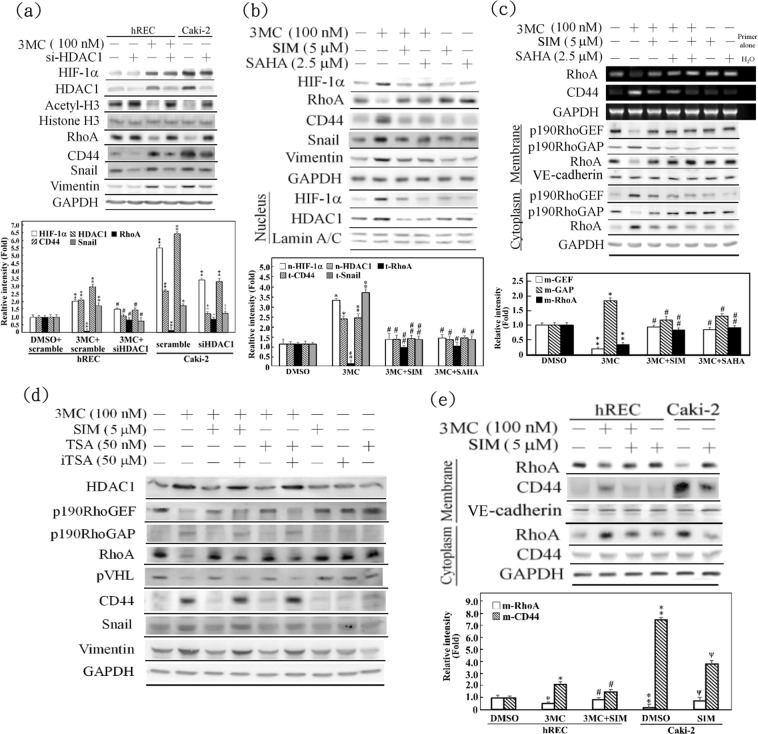
Gain of function of RhoA caused by simvastatin through reducing HDAC1 and p190RhoGAP, and increasing p190RhoGEF expression in 3MC-treated cells. (**a**) hRECs were transfected with siHDAC1 overnight, followed by a 3-h 3MC challenge. The effect of HDAC knockdown in 3MC-mediated tumor-promoting effect was examined by Western blot analysis of the indicated molecules. (**b**,**c**) hRECs were pretreated with SAHA or simvastatin (SIM) for 1 h, followed by a 3-h 3MC challenge, and the resulting cell lysates or cytosolic, nuclear, or membrane fractions were analyzed by Western blots or RT-PCR, with GAPDH, lamin A/C, and VE-cadherin as input controls for the corresponding preparations. (**d**) hRECs were sequentially pretreated with either DMSO or ITSA (50 μM, an HDAC activator via TSA suppression) for 5.5 h, TSA (50 nM, an HDAC1 inhibitor) for 18.5 h or SIM (5 μM) for 1 h, followed by 3MC treatment for 3 h. The resulting cells were harvested and analyzed for the indicated proteins by western blots. (**e**) hRECs and Caki-2 cells were also pretreated with simvastatin for 1 h prior to a 3-h 3MC challenge to compare the therapeutic effect of simvastatin in Caki-2 malignancy with its protection in normal hRECs. The resulting cell lysates were partitioned into cytosolic–membrane fractions to analyze the activated forms of RhoA and CD44 in the membrane. The bar chart and Table S2 show the normalized intensity of each protein band obtained using GAPDH in Western blots. Results are expressed as the mean ± SD (n = 3). Data from a representative experiment are shown. Significant difference: *P < 0.05 and **P < 0.01 vs. control; ^#^P < 0.05, and ^##^P < 0.01 vs. 3MC treatment alone; ^+^P < 0.05 and ^++^P < 0.01 vs. Caki-2 with scramble siRNA treatment; ^ψ^P < 0.05 vs. Caki-2 + DMSO. The gels have been run in the same experimental conditions and the cropped blots were shown. The entire gel pictures were shown in the Supplemental Fig. 2.

We further explored the interdependent relationship of HDAC and RhoA in 3MC-treated hRECs. Specifically, whether 3MC-mediated HDAC1 upregulation is responsible for reduced RhoA expression was investigated in cells transfected with siHDAC1. The siHDAC1 reversed 3MC-induced suppression of RhoA levels in hRECs and alleviated EMT markers and CD44 upregulation, as revealed by Western blot analysis (Fig. 2a). Lin *et al*.^14^ demonstrated the HDAC-inhibitory effect of simvastatin. These findings are consistent with the findings of the present study, which also indicated that simvastatin exhibited similar effects of SAHA and reversed 3MC-mediated decrease in RhoA level. Enhanced RhoA function, promoted by simvastatin and SAHA, was correlated with the inhibition of EMT markers and CD44 upregulation in 3MC-treated hRECs (Fig. 2b). Similarly, reverse transcription (RT) polymerase chain reaction (PCR) results demonstrated that the HDAC1-inhibitory effect of simvastatin and SAHA transcriptionally increased RhoA mRNA levels and reduced CD44 mRNA levels. In addition to inducing RhoA expression, simvastatin and SAHA increased the levels of active RhoA in the membrane, in conjunction with increased p190 Rho guanine nucleotide exchange factor (p190RhoGEF, a RhoA activator) and reduced p190 Rho family GTPase-activating protein (p190RhoGAP, a RhoA inhibitor) expression in 3MC-treated hRECs (Fig. 2c). Similar to the effect of TSA, simvastatin alleviated the detrimental effect of 3MC in hRECs (Fig. 2d). By contrast, HDAC activation by ITSA diminished the protective effects of simvastatin on 3MC-mediated EMT marker and CD44 upregulation, suggesting the anti-HDAC activity of simvastatin. The protective effect of simvastatin was also observed in Caki-2 cells (a ccRCC cell line) and was indicated by an increase in the active form of RhoA in the membrane and a decrease in membrane-bound CD44 (Fig. 2e). These results demonstrated that simvastatin not only protected hRECs from 3MC-mediated alterations but also alleviated CD44 in Caki-2 cells.

### AhR-dependent effect of 3MC on RhoA inhibition and simvastatin-mediated RhoA reactivation protected hRECs against the tumor-promoting effect of 3MC

**Figure 3:**
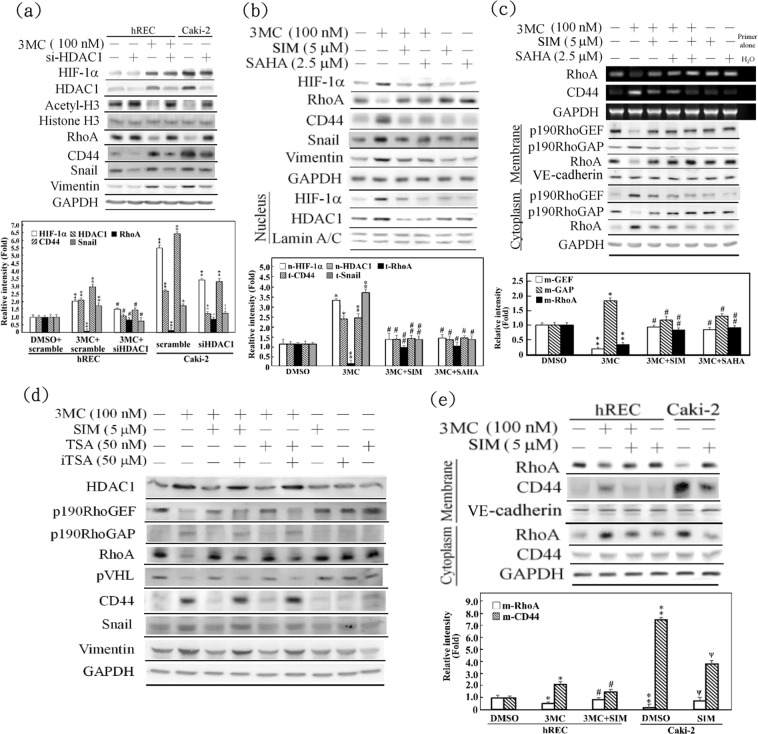
An essential role of RhoA in 3MC-mediated hREC carcinogenesis, in part, through an AhR-dependent mechanism. Cells were transfected with siAhR overnight or pretreated with simvastatin for 1 h, followed by a 3-h 3MC challenge, and the resulting cells were subjected to Western blot analysis (**a**) and immunofluorescence staining (**b**) for the indicated molecules. Scale bar = 25 µm. (**c**) Cells were pretreated with calpeptin and Y27632 (a ROCK inhibitor) for 1 h, followed by 3-h 3MC treatment, and the resulting cell lysates were analyzed by Western blots, with GAPDH used as an internal control. (**d**) hRECs were transfected with CARhoA overnight, and pretreated with calpeptin for 1 h or sorafenib for 5 h, followed by 3MC challenge for 3 h. Western blot analysis of the indicated proteins were carried out to compare the effects of CARhoA overexpression, simvastatin and calpeptin with sorafenib in hREC carcinogenesis caused by 3MC. (**e**) Caki-2 cells were transfected with empty or CARhoA overexpression vector overnight, followed by 1 h of simvastatin and calpeptin, or 5 h of sorafenib treatment. Western blot analysis of the EMT and hypoxia markers was carried out to evaluate the synergistic effects of the combination treatment of simvastatin, CARhoA overexpression (or calpeptin), and sorafenib in alleviating RCC progression. The bar chart and Table S3 show the normalized intensity of each protein band obtained using GAPDH or α-tubulin in Western blots. (*P < 0.05 and **P < 0.01 vs. control; ^#^P < 0.05 and ^##^P < 0.01 vs. 3MC treatment alone). The gels have been run in the same experimental conditions and the cropped blots were shown. The entire gel pictures were shown in the Supplemental Fig. 3.

After elucidating the crucial role of HDAC1 in RhoA regulation, we further examined the AhR-dependent effect of 3MC-induced RhoA downregulation. As depicted in Fig. 3a, RhoA levels were restored in 3MC-treated hRECs with siAhR-mediated AhR knockdown, and Cyp1A1 was used as a positive control for evaluating the effect of 3MC. This finding suggested that 3MC-mediated RhoA downregulation was AhR-dependent. Furthermore, simvastatin and siAhR abolished the effects of 3MC on EMT marker upregulation, accompanied by reduced HIF1α, ARNT2, HDAC1, p190RhoGAP, CD44, Snail and vimentin expression, and increased p190RhoGEF, RhoA, and E-cadherin expression (Fig. 3a). As displayed in Fig. 3b, immunofluorescence staining revealed that 3MC significantly reduced RhoA and E-cadherin levels and increased vimentin expression in hRECs. These expression levels resembled those in Caki-2 cells. These effects were abolished with simvastatin treatment, which restored RhoA levels in Caki-2 cells and 3MC-treated hRECs (Fig. 3b). To validate the critical role of RhoA inhibition in 3MC-mediated EMT maker upregulation, cells were pretreated with calpeptin, a calpain inhibitor with RhoA-activating properties, and Y27632, an inhibitor of the RhoA downstream target Rho-associated protein kinase (ROCK). As displayed in Fig. 3c, the results indicated that calpeptin prevented 3MC-mediated EMT marker upregulation in cells, accompanied by reducing p190RhoGAP, CD44, Snail and vimentin levels and by increasing RhoA, p190RhoGEF, and pVHL, whereas Y27632 mimicked the effect of 3MC treatment.

The crucial role of RhoA activity in 3MC-mediated alterations in molecular phenotypes was genetically validated; constitutively active (CA) was overexpressed, and the results suggested that CA-RhoA attenuated the tumor-promoting effect of 3MC (Fig. 3d). Furthermore, we compared the therapeutic effects of calpeptin (a RhoA inducer), CA-RhoA overexpression, and simvastatin (an HDAC inhibitor) with those of the sorafenib intervention on hREC carcinogenesis caused by 3MC treatment. The efficacy of simvastatin, CA-RhoA, and calpeptin was similar to that of sorafenib. In addition, the synergistic effects of the combined treatment of genetic or pharmacological RhoA activation, simvastatin, and sorafenib were evaluated in Caki-2 cells (Fig. 3e). The combination regimen suppressed EMT marker upregulation and increased RhoA and pVHL levels compared with the conventional targeted therapy of sorafenib in our experimental settings. Thus, the combination regimen can be further developed as RCC treatment.

### Genomic downregulation of RhoA by 3MC through AhR- and HIF1α-dependent mechanisms

**Figure 4:**
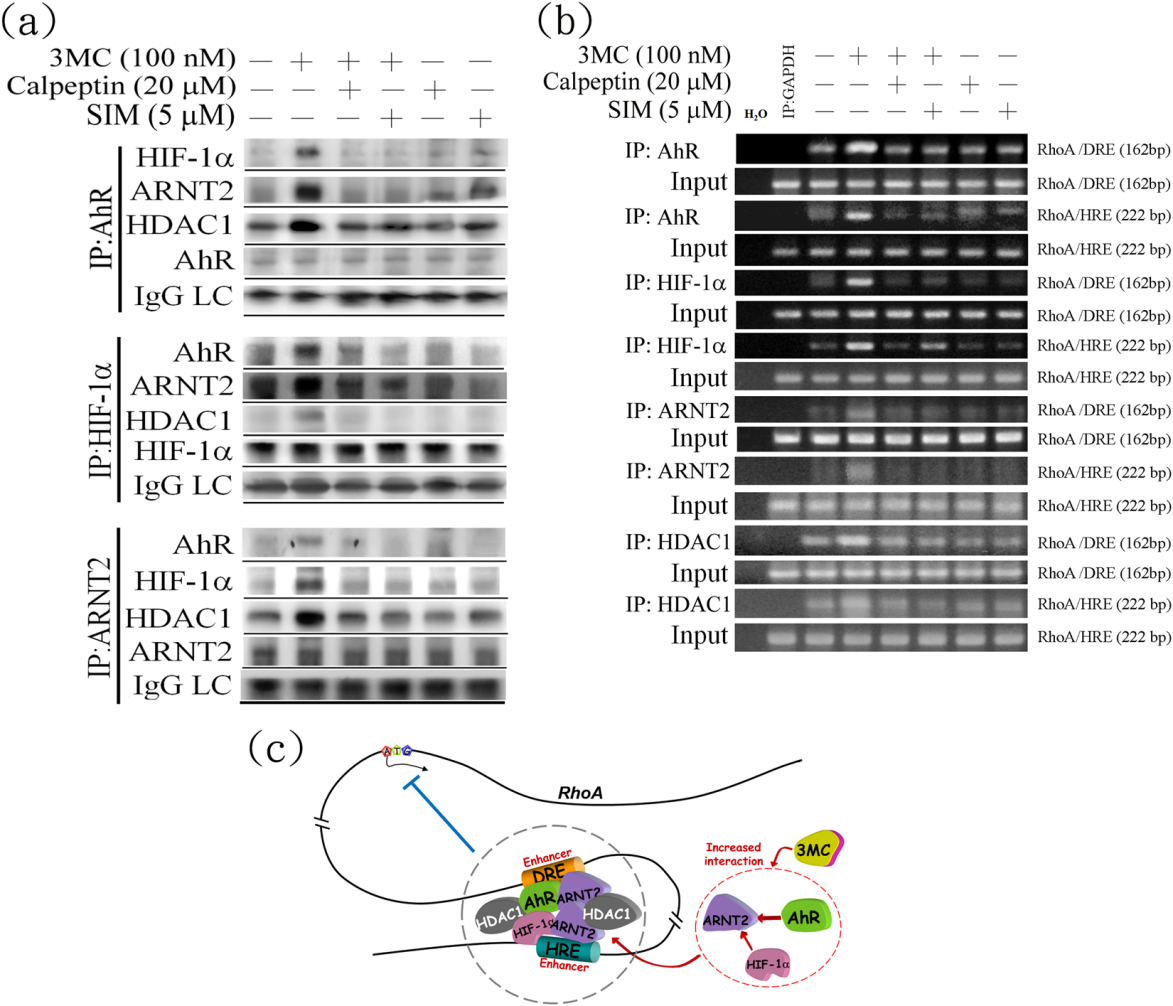
Genomic downregulation of RhoA through increased recruitment of HDAC1 to a complex of AhR–HIF1α–ARNT2 in 3MC-treated hRECs. (**a**) Cells were pretreated with 1 h of simvastatin (SIM) and calpeptin (CAPT), followed by 3 h of 3MC challenge, which were harvested and assayed for protein–protein interaction by using anti-AhR, -HIF1α, or -ARNT2 antibodies. The counterparts of associated proteins were hybridized with the indicated antibodies in Western blot analysis. (**b**) Cells with similar treatments were harvested for ChIP assay analysis. The indicated antibodies of transcription factors were utilized to pull down the association complexes of the transcription factors and DNA; the interacted DNA was amplified by pairs of primers flanking the conserved regions of DRE and HRE in the RhoA promoter. The immunoprecipitation was normalized to each amount of the corresponding antibody pulled down, and an IgG light chain (L chain) was used as an antibody-loading control. The gels have been run in the same experimental conditions and the cropped blots were shown. The entire gel pictures were shown in the Supplemental Fig. 4, and the data are quantified and shown in Table S4. (**c**) A model of the molecular mechanism underlying the genomic downregulation of RhoA through increased HDAC1 recruitment to the AhR and HRE complex in hRECs treated with 3MC.

Because 3MC mediated RhoA downregulation in an AhR-dependent manner through HIF1α-mediated HDAC1 upregulation, we hypothesized that both AhR and HIF are involved in transcriptional RhoA reduction. As illustrated in Fig. 4a, a coimmunoprecipitation (co-IP) assay performed using anti-AhR, anti-HIF1α, and anti-ARNT2 antibodies revealed that the transcription factors of HIF1α, ARNT2, AhR, and HDAC1 formed a complex in 3MC-treated hRECs. Additionally, 3MC increased the recruitment of HDAC1 to the HIF1α–ARNT2–AhR complex, but simvastatin and calpeptin treatments diminished this effect (Fig. 4a). In addition, we identified dioxin-responsive element (DRE) and hypoxia-responsive element (HRE) binding sites in the RhoA promoter region by using Promo and MatInspector software. The conserved sequences of DRE and HRE were located at −422 to −446 bp and −1142 to −1150 bp, respectively, in the RhoA promoter region. The chromatin immunoprecipitation (ChIP) assay demonstrated that 3MC increased the formation of large complexes, including the binding of AhR and HIF1α to the DRE and HRE sites, in conjunction with increased HDAC1 recruitment to the RhoA promoter. These complexes were pulled down using anti-AhR, anti-HIF1α, and anti-ARNT2 antibodies. Simvastatin and calpeptin reduced the complex formation in 3MC-treated hRECs (Fig. 4b). Figure 4c presents a schema depicting the genomic regulation of AhR and HIF for 3MC-mediated RhoA downregulation.

### Functional assessment of the carcinogenic effects of 3MC and simvastatin’s corresponding protective effect *in vitro* and *in vivo*

**Figure 5:**
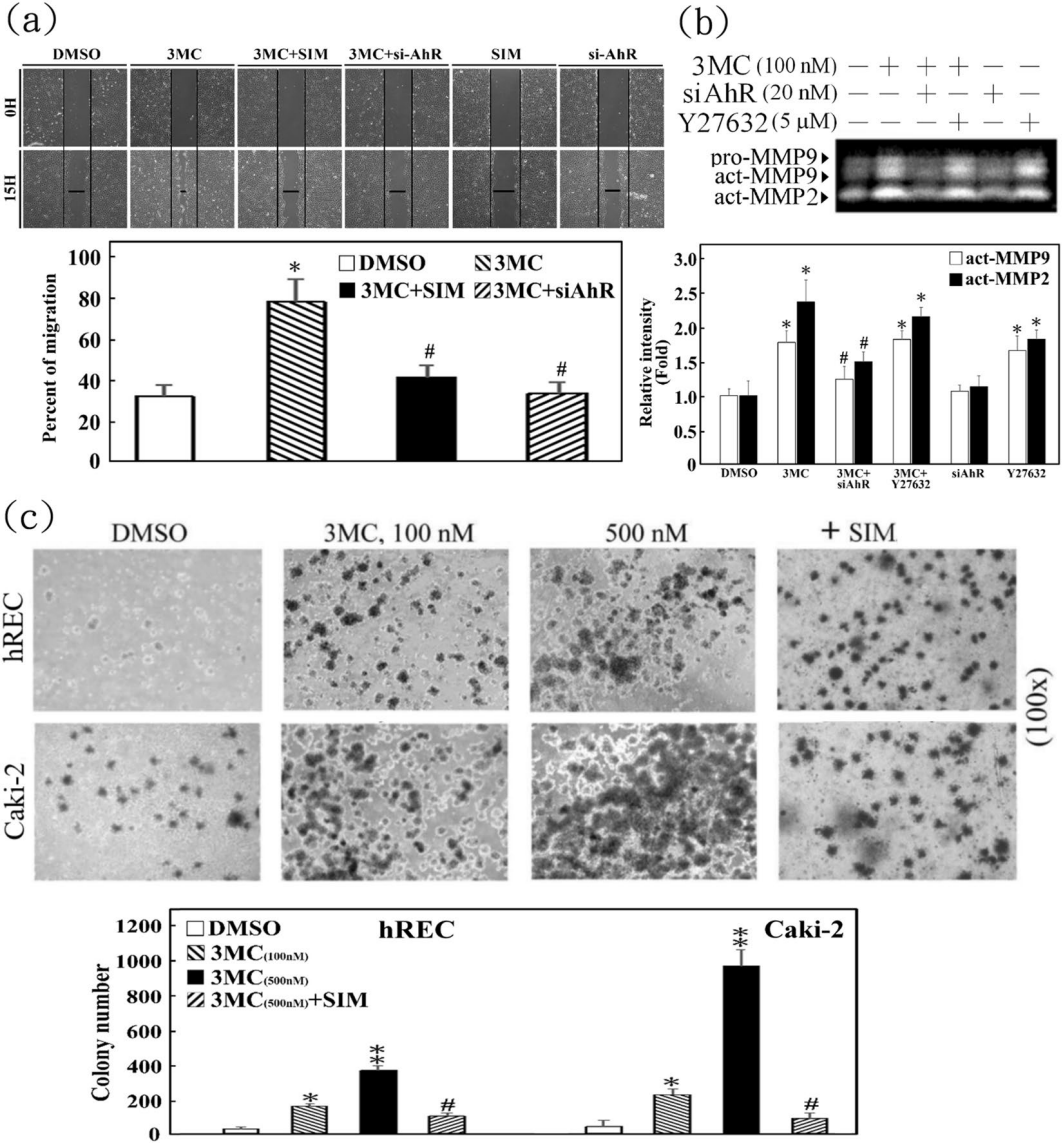
Functional assessment of RhoA gain of function caused by simvastatin for preventing 3MC-mediated increases in REC carcinogenesis. (**a**) A wound-healing assay was performed to assess the antimigratory effect of simvastatin in 3MC-treated hRECs. Magnification, ×100. The percent of cell migration at 15 h was quantitated by ImageJ and displayed as bar graphs. (**b**) The AhR knockdown and RhoA inactivation effect of Y27632 in MMP-2 and -9 activation were examined through a zymography assay. The bar charts show the band intensities of the indicated proteins normalized with those of the control using densitometry. (**c**) Caki-2 cells and hRECs were treated with increasing concentrations of 3MC (100–500 nM) for 3 weeks and assayed for colony formation in a soft-agar colony formation assay. The number of colonies was counted and displayed as bar graphs. The gels have been run in the same experimental conditions and the cropped blots were shown. The entire gel pictures were shown in the Supplemental Fig. 5. Results are expressed as the mean ± SD (n = 3). Data from a representative experiment are shown. Significant difference: *P < 0.05 and **P < 0.01 vs. DMSO-treated group; ^#^P < 0.05 vs. 3MC treatment alone.

The EMT and carcinogenic effects of 3MC were assessed using hRECs and Caki-2 cells. A wound healing assay indicated that 3MC increased hREC migration after 15 h of treatment (Fig. 5a) and concomitantly increased matrix metallopeptidase (MMP)-2 and MMP-9 activation. Gel zymography analysis revealed these effects in 3MC-treated hRECs (Fig. 5b). The effects were abolished through simvastatin treatment and siAhR transfection (Fig. 5a), and AhR silencing was mimicked by the ROCK inhibitor Y27632 (Fig. 5b). A colony formation assay demonstrated that 3MC concentration dependently increased the clonogenic effect in normal hRECs and cancerous Caki-2 cells after 3 weeks of treatment, suggesting the potency of 3MC in the onset and progression of renal tumors. Simvastatin significantly diminished the effects of 3MC in this *ex vivo* functional assay (Fig. 5c).

**Figure 6:**
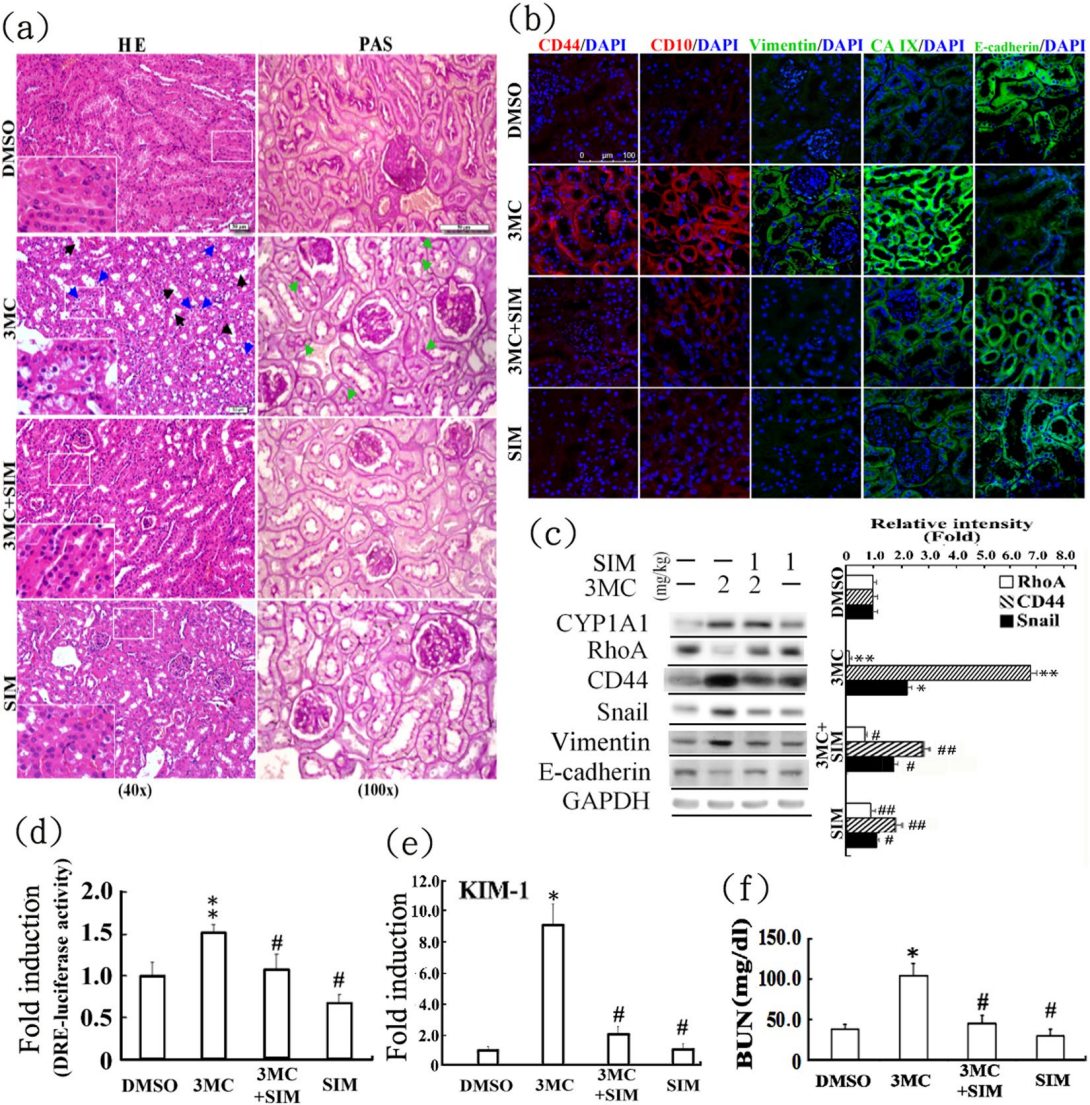
*In vivo* assessment of protection against 3MC-mediated renal carcinogenesis with concomitant EMT marker upregulation by simvastatin. Murine kidneys were dissected and sectioned for (**a**) histological examination through HE and PAS staining. Blue arrow: the loss of cytoplasm in renal proximal tubules; black arrow: tubular dilation and epithelial flattening; and green arrow: PAS-positive. Insets at the lower-left corners are higher magnifications of the images in white squares and representative photographs are shown. White scale bar represents 50 μm. Effect of 3MC in RCC onset was analyzed by immunofluorescence staining (**b**) and Western blots (**c**) for RCC biomarkers (e.g., CD44, CD10, and carbonic anhydrase IX [CA IX]) and EMT markers (e.g., Snail, vimentin and E-cadherin). Scale bar in white = 100 µm. Cyp1A1 was used as a positive control for 3MC’s action. The bar charts and Table S5 show the band intensities of the indicated proteins normalized using densitometry with GAPDH. The gels have been run in the same experimental conditions and the cropped blots were shown. The entire gel pictures were shown in the Supplemental Fig. 6. (**d**) hRECs were transiently transfected with pGL2/3DRE and phRL-TK for 24 h. Cells were pretreated with 20 µL of murine serum for 2 h prior to challenge with the indicated concentrations of 3MC. Luciferase activities of the reported plasmid were normalized to those of the internal control plasmid. Data are presented as the mean ± SD. (**e**) In mice who received the indicated treatments, the integrity of the renal cells was evaluated through renal injury marker, KIM1, by using QPCR. (**f**) Renal function was assessed through blood urea nitrogen (BUN) level after 3 months of treatment. The results are expressed as the mean ± SD (n = 5; *P < 0.05 vs. control; ^#^P < 0.05 vs. 3MC-treated group).

The aforementioned effects were validated in mice injected with 3MC twice a week for 3 months. Histological examination using hematoxylin and eosin (HE) staining revealed that 3MC-treated mice exhibited similar features to those of ccRCC, involving the loss of cytoplasm in renal proximal tubules (blue arrows) and tubular dilation and epithelial flattening (black arrows) (Fig. 6a). Similarly, more periodic acid–Schiff (PAS)-positive cells (green arrows) were observed in the renal sections of the 3MC-treated group than in those of the control and simvastatin groups, suggesting abundant glycogen and lipid deposition in the 3MC samples. The immunofluorescence staining results (Fig. 6b) revealed increased CD44, CD10, vimentin, and carbonic anhydrase IX (a ccRCC biomarker) expression and decreased E-cadherin levels in the kidneys of 3MC-treated mice. Consistent with the results of immunofluorescence staining, Western blot analysis of murine renal tissues indicated that 3MC treatment increased the expression of EMT and CSC markers, compared with CYP1A1 (positive control; Fig. 6c). Simvastatin reversed the 3MC-induced effects of EMT and RCC marker upregulation. AhR activation was evaluated in a DRE-driven luciferase assay (Fig. 6d), which revealed that serum obtained from the 3MC group exhibited 1.5-fold higher DRE activity than did serum from the control group. Simvastatin treatment significantly reduced this effect, suggesting that simvastatin exerted an inhibitory effect on AhR activity. To determine the effect of 3MC on renal injury, the level of kidney injury molecule 1 (KIM1) was assessed through quantitative PCR analysis. The results displayed in Fig. 6e suggest that simvastatin treatment significantly reduced the 3MC-induced elevated expression of KIM1. Additionally, 3MC treatment impaired murine renal function, as indicated by an increase in serum blood urea nitrogen (BUN) level, but simvastatin treatment also reversed this effect (Fig. 6f).

## Discussion

**Figure 7:**
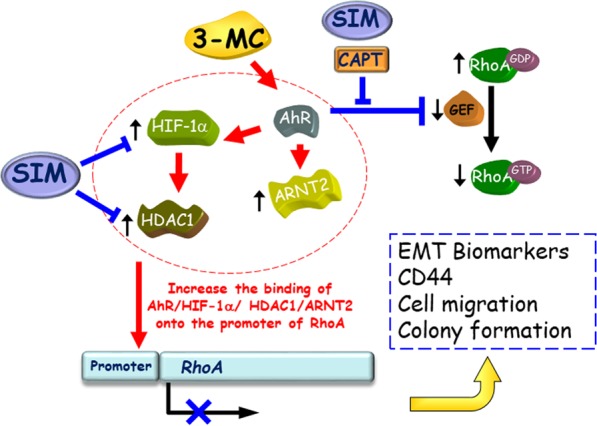
A schematic diagram of the inverse relationship between HDAC1 and RhoA in 3MC-mediated tumor-promoting effect through AhR- and HIF1α-dependent mechanisms. The effect of 3MC on RhoA inhibition can be prevented by simvastatin through HDAC1 inhibition. Notably, the restoration of RhoA by simvastatin and calpeptin reversed the effects of 3MC on HIF1α-mediated upregulation of EMT biomarkers and CD44, cell migration and colony formation.

In the present study, we revealed that 3MC reduced pVHL levels and activated the hypoxia signaling pathway by increasing HIF1α levels. 3MC upregulated HDAC1, inhibited RhoA expression through a HIF-AhR-HDAC1-dependent mechanism, and reduced expression of p190RhoGEF, a RhoA activator. This study demonstrated the novel finding that RhoA function loss plays a critical role in 3MC-treated cells. The results also indicated that simvastatin inhibited the effects of 3MC by reactivating RhoA through HDAC1 downregulation, as summarized in Fig. 7.

Consistent with these findings, 50% of RCC cases exhibit silencing of essential tumor suppressor genes, and this effect is associated with HDAC1 and HDAC2 upregulation^27^. Accordingly, an increasing number of studies are being performed to validate the effectiveness of HDAC inhibitors for RCC interventions^28^. Previous studies have examined the interdependent relationship between HDAC1 and HIF1α and have determined that HDAC1 increases HIFα stability through deacetylation^29^. In the present study, we demonstrated that the hypoxia signaling activated by HIF1α induction increased HDAC1 expression, resulting in loss of RhoA function and subsequently detrimental events in hRECs. Additionally, previous studies have demonstrated that HDAC epigenetically modifies nucleosomes and increases hypoxia-mediated VEGF induction^30^, leading to angiogenesis. Therefore, the synergistic effects of HIF1α and HDAC may form a vicious cycle promoting RCC progression, and these phenomena warrant further investigation.

Although the effects of RhoA activation in the progression of various cancers are still contradictory^1,24–26,31^, both the *in vitro* and *in vivo* results of our study demonstrated that 3MC-induced RhoA inhibition contributed to RCC-tumor-promoting effects. The protective property of RhoA reactivation suggested in the present study is consistent with findings of a recent study, in which honokiol-induced RhoA reactivation protected cells from RCC malignancy^32^, In this study, simvastatin-induced RhoA upregulation created negative feedback for 3MC-mediated HIF1α induction. This phenomenon might be attributable to upregulation of pVHL to block 3MC-induced hypoxia signaling, consistent with the findings of Zhu *et al*.^33^. Thus, simvastatin may function as a deacetylase inhibitor to restore *RhoA* gene expression, which is consistent with previous findings that statin increased p21 through HDAC1/2 inhibition^14^.

Clinically, simvastatin is administered in doses of 5–80 mg to reduce cholesterol in patients with hyperlipidemia. A dose of 1 mg/kg simvastatin was used in our mice model, and this treatment exerted a protective effect against 3MC-mediated renal injury. The dose in the mice model was approximately equivalent to 12 mg for humans, and the results suggested that simvastatin may be a feasible therapeutic approach. Statins are common treatment for cardiovascular disease in some elderly patients with cancer. The use of statins in chemotherapy regimens warrants further evaluation. A previous study indicated that statin use improved survival among patients with metastatic RCC^19^. Additionally, statin use is associated with lower progression after surgery for localized RCC^18^. A recent systematic review and meta-analysis suggested that statin use significantly reduces cancer-specific and all-cause mortality among patients with RCC^20^. This finding suggests that statin may serve as adjunct therapy for RCC. In this study, the combination of simvastatin with calpeptin or sorafenib exhibited stronger effects on reducing HIF1α upregulation and EMT markers and increasing the pVHL level *in vitro* than sorafenib alone. These effects are currently under investigation for optimization.

Exogenous AhR ligands promote tumor progression; however, their tumor initiation effects remain unknown^21–23^. The results of our study suggested the tumor-promoting effects of 3MC in hRECs; 3MC initiated EMT induction and induced cells to exhibit RCC features, including increased migration and invasion, colony formation, and CD44 expression. Further investigations are being performed to determine the *in vivo* effect of 3MC. In addition, a previous study indicated that AhR activation increased hypoxia activation in human trophoblastic JAR cells^34^, further supporting the crucial role of AhR in 3MC-mediated upregulation of hypoxia-related proteins. We also demonstrated the detailed molecular mechanism underlying HIF1α-mediated RhoA suppression, which occurred through an increase in HDAC recruitment to the DRE and HRE binding sites in the RhoA promoter. Additionally, the results of our study confirmed the complexity of AhR–ARNT and HRE–ARNT, which formed large complexes and were subject to HDAC regulation (Fig. 4a). Consistent with the *in vitro* findings, EMT marker upregulation, RhoA downregulation, CD44 overexpression, and tumor phenotypes of ccRCC were observed in mice with chronic exposure to 3MC. Increasing evidence indicates reciprocal expression of RhoA and CD44 in RCC, namely reduced RhoA and increased CD44 expression^8^. In the present study, we discovered that simvastatin-mediated RhoA reactivation reduced CD44 expression in 3MC-treated hRECs. However, the molecular mechanisms underlying the effects of RhoA downregulation, in conjunction with CD44 upregulation, on RCC progression require further investigation.

To the best of our knowledge, this study is the first to demonstrate the tumor-promoting effects of 3MC in inducing RCC phenotypes through HIF1α-associated RhoA downregulation *in vitro* and *in vivo*. Inhibition of HIF1α and HDAC1 and simvastatin-induced reactivation of RhoA significantly protected cells from 3MC-mediated effects. Statins are not administered as antitumor agents because the efficacy of such treatment has not been proven in clinical trials^35^, but the beneficial effects of simvastatin presented in this study suggest that simvastatin can serve as adjunct therapy for RCC. Prospective evaluation of the proposed treatment in clinical trials is required.

## Methods

### Cell culture and reagents

hRECs (PCS-400-012) and human RCC cells (Caki-2, ACHN, and 786**-**o) were purchased from the American Type Culture Collection (Manassas, VA, USA) in May 2015. Caki-2, 786-o, and ACHN cell lines were authenticated through short tandem report profiling on the Promega GenePrint 10 System and were analyzed using ABI PRISM 3730 GENETIC ANALYZER and GeneMapper Software V3.7. The cell lines were routinely tested and were last examined in February and June 2017. Except for hRECs, which were cultured in a renal epithelial cell basal medium (Clonetics^TM^ REGM^TM^) containing 0.5% serum and epithelial growth factor (Lonza Inc., Walkersville, MD, USA), all cells were maintained in Dulbecco’s modified Eagle’s medium (DMEM) supplemented with 10% fetal bovine serum (FBS). Cells were grown to 85%–95% confluence before use. DMEM, FBS, and tissue culture reagents were obtained from Life Technologies (Gaithersburg, MD, USA). Cells from passages 5 to 15 were used in the experiments. Additional reagents were purchased from the following sources: 3MC from Supelco (Bellefonte, PA, USA), simvastatin from Sigma-Aldrich (St. Louis, MO, USA), Y27632 from Calbiochem (San Diego, CA, USA), TSA and N-(1H-benzotriazol-1-yl)-2,4-dichlorobenzamide (ITSA, which activates HDAC through TSA suppression) from Santa Cruz (Dallas, TX, USA), and calpeptin (a calpain inhibitor with RhoA-activating activity)^36^ from Enzo Life Science **(**Farmingdale, NY, USA). The concentrations of chemicals and the duration of treatments for each assay were according to those in our previous publications^37–39^ or pilot studies.

### Preparation of cell fractions (nucleus, cytosol, and membrane) and Western blot analysis

Cells grown in 6- or 10-cm^2^ dishes were harvested after the indicated treatments and were partitioned into cytosolic and nuclear fractions by using NE-PER^TM^ nuclear extraction reagents (Pierce, Rockford, IL, USA) with protease inhibitors, according to the manufacturer’s instructions. To prepare membrane–cytosolic fractions, cells were collected after the indicated treatments and were incubated in 0.1 mL of hypotonic buffer (10 mM Tris [pH 7.5], 0.5 mM EDTA, and 2 mM phenylmethylsulfonyl fluoride) at 4 °C for 30 min. After centrifugation, the supernatant (cytosolic fraction) was collected, and the pellet was resuspended in 0.1 mL of radioimmunoprecipitation assay buffer and incubated at 4 °C for 30 min. The resulting fractions were sheared 100 times through an insulin syringe with a 29-G needle. After centrifugation, the supernatant (membrane fraction) was collected for analysis. Western blotting was performed as described elsewhere^40^ and antibodies are displayed in Table S6. The photos of protein bands in western blots were taken using an image analysis system (UVP BioChemi, UVP LLC, Upland, CA, USA) and their intensities were measured using ImageJ software.

### Luciferase activity assay, transfection of AhR/HDAC1 small interfering RNAs and CARhoA plasmid, and RT and quantitation (Q) PCR analysis

Oligonucleotides with three copies of the DRE (GAGTTGCGTGAGAAGAGCC) and HRE (GCCCTACGTGCTGTCTCA) obtained from rat CYP1A1 and human erythropoietin enhancer, respectively, were synthesized and cloned into the KpnI and NheI sites of the pGL2-promoter vector (Promega, Madison, WI, USA)^41,42^. The identities of the sequences were confirmed using an ABI PRISM 377 DNA Analysis System (Perkin-Elmer). hRECs transiently transfected with the DRE or HRE-luciferase plasmid and phRL-TK (an internal control) overnight were pretreated with serum (20 μL/well in a 24-well plate) or other indicated treatments for 2 h and were then harvested for a luciferase activity assay performed using a dual luciferase reporter assay system (Promega) in a TD-20/20 luminometer (Turner Designs, Madison, WI, USA).

AhR small interfering RNAs (siRNAs) and HDAC1 siRNAs duplexes were chemically synthesized at Ambion (Austin, TX, USA) and MDBio, Inc. (Taipei, Taiwan). The pairs of oligonucleotides are displayed in Table S7. hRECs were seeded in 6-well plates and transfected with 5 pmol of AhR siRNA or 20 pmol of HDAC1 siRNA in a volume of 100 μL by using a TransIT-TKO transfection reagent (Mirus Bio Corporation, Madison, WI, USA). CARhoA cDNA (Q63L RhoA) in pUSEamp was purchased from Millipore (Burlington, MA). hRECs and Caki-2 cells were transfected with pUSEamp-overexpressing CARhoA (6 μg/10-cm Petri dish) overnight by using Lipofectamine 2000 (Invitrogen, Carlsbad, USA). After transfection for 4–6 h, cells were plated in DMEM supplemented with 10% FBS and incubated overnight.

For RT-PCR and QPCR analyses, total RNA was obtained using a previously described method^39^ with minor modifications. Sequences of the primer pairs for amplification of each gene are presented in Table S8.

### Co-IP and ChIP assays

Cells grown in 10-cm^2^ dishes were treated as indicated and were harvested as described for Western blot analysis. Protein molecules of AhR, HIF1α, or ARNT2 were immunoprecipitated from 100 µg of cell lysates by using anti-AhR, anti-HIF1α, or anti-ARNT2 antibodies (2 µg) and protein A and G agarose beads (20 µg). The precipitates were washed five times with lysis buffer and once with phosphate-buffered saline. The pellet was then resuspended in sample buffer (50 mM Tris [pH 6.8], 100 mM bromophenol blue, and 10% glycerol) and incubated at 90 °C for 10 min to release the proteins from the beads before electrophoresis. A ChIP assay was performed according to the instructions of Upstate Biotechnology (Lake Placid, NY, USA) with minor modifications. In brief, 6 × 10^5^ cells with the indicated treatments in 10-cm^2^ dishes were harvested. For the co-IP assay, 100 µg of the total cell lysate was incubated overnight with anti-AhR, anti-HIF1α, anti-ARNT2, or anti-HDAC1 antibodies. The DNA filtrates were amplified through PCR by using primers flanking the promoter of the RhoA gene containing the putative HRE or DRE binding site. Sequences of the primer pairs used for amplifying the enhancer regions of the RhoA promoter are displayed in Table S9. Double-distilled H_2_O replaced the DNA template as a negative internal control. The PCR program of the MasterMix system involved a denaturing step at 95 °C for 5 min, followed by 40 cycles at 94 °C for 30 s, 53 or 55 °C for 20 s, and 72 °C for 20 s. An anti-GAPDH antibody was used as a negative control for nonspecific reactions. The PCR products were electrophoresed on a 2% agarose gel; products of the expected sizes of 222 bp and 162 bp for RhoA/HRE and RhoA/DRE, respectively, were visualized and quantified using an image analysis system.

### Gelatin zymography

The gelatinase activity of MMP-2 and MMP-9 in conditioned medium used for culturing 3MC-treated hRECs was evaluated through gelatin zymography on modified SDS-PAGE gel^43^. Briefly, the conditioned medium was centrifuged (10,000 g for 5 min at 4 °C), and an equal volume (25 µL/5 mL at 10 cm^2^) was mixed with nonreducing sample buffer (240 mM Tris-hydrochloride [Tris-HCl; pH 6.8], 40% glycerol, and 0.2% bromophenol blue). The mixture was then electrophoresed on 7.5% SDS-PAGE gel with 0.1% gelatin at room temperature. The resulting gel was washed three times at room temperature with washing buffer I (50 mM Tris-HCl [pH 7.4] and 2% Triton-X100) for 15 min and was then washed three times with buffer II (50 mM Tris-HCl [pH 7.4]) for 5 min, followed by the application of the developing buffer (50 mM Tris-HCl [pH 7.4], 0.02% NaN_3_, 0.02 mM zinc chloride, 5 mM calcium chloride, and 200 mM sodium chloride) at 37 °C for 24 h. The gel was subsequently stained and destained with conventional solutions. The molecular size of each gelatinolytic band was evaluated using protein standard ladders (Thermo Fisher Scientific, Waltham, MA, USA).

### Soft agar colony formation assay

Soft agar comprising 0.5% agar in 1.5 mL of DMEM supplemented with 10% FBS was added to 6-well plates to form the bottom layer, and the plates were incubated at 37 °C in a humidified incubator for agar solidification. An additional 1.5 mL of 0.3% agar containing suspended hRECs or Caki-2 cells (5 × 10^3^ cells/well) was added on top of the bottom layer, and 100 µL of medium was added twice a week to maintain humidity. Nitroblue tetrazolium chloride was used to stain formed colonies for alkaline phosphatase activity, and the colonies were then visualized under a microscope^44^.

### *In vivo* effect of 3MC on renal carcinogenesis associated with EMT induction

All animal study procedures were conducted in accordance with the Taipei Medical University Animal Care and Use rules (license no. LAC-2015-0206) and were reviewed and approved by the Institutional Animal Care and Use Committee and Panel. Eight-week-old male Balb/c mice weighing 20–25 g were obtained from the National Laboratory Animal Center (Taipei, Taiwan). The animals were housed in a central facility under a 12-h light–dark cycle and were provided with regular rat chow and tap water. The mice were divided into control, 3MC (2 mg/kg), 3MC with simvastatin (1 mg/kg), and simvastatin alone groups (n = 5). They were then intraperitoneally administered 3MC, 3MC with simvastatin, or simvastatin three times a week for 3 months. At the end of the treatment period, the animals were intramuscularly anesthetized using a combination of ketamine (8 mg/100 g body weight), xylazine (2 mg/100 g), and atropine (0.16 mg/100 g). Their blood samples were collected for assessment of BUN levels using Fuji Dri-Chem slides (Fujifilm, Tokyo, Japan). One kidney from each mouse was removed through laparotomy and was snap-frozen in dry ice for the Western blot analysis of EMT-associated markers. The other kidney was fixed in 10% formalin and embedded in paraffin. Serial 5-µm sections of these samples were stained with HE, PAS, and immunofluorescence (IFC) stains for histological analysis, as described previously^40,45^. The procedures of IFC and PAS staining were included in Supplementary Information. Histological examination was performed by a pathologist in a single-blind manner.

### Statistical analysis

Data are presented as the mean ± SD of at least three experiments. Differences in the means of two groups were calculated using unpaired Student’s *t* tests, and differences among multiple groups were determined using one-way analysis of variance. The Bonferroni method was used for post hoc analysis. *P* < 0.05 was considered significant.

## Supplementary information

Supplementary Information

Raw data
