# Author Correction: Simvastatin reduces the carcinogenic effect of 3-methylcholanthrene in renal epithelial cells through histone deacetylase 1 inhibition and RhoA reactivation

**DOI:** 10.1038/s41598-020-69592-w

**Published:** 2020-07-22

**Authors:** Chih-Cheng Chang, Kuo-How Huang, Sung-Po Hsu, Yuan-Chii G. Lee, Yuh-Mou Sue, Shu-Hui Juan

**Affiliations:** 1 Department of Physiology, School of Medicine, College of Medicine, Taipei Medical University, Taipei, Taiwan; 2 National Taiwan University Hospital; Department of Urology, College of Medicine, National Taiwan University; and National Taiwan University Hospital, Taipei, Taiwan; 3 Graduate Institute of Biomedical Informatics, College of Medical Science and Technology, Taipei Medical University, Taipei, Taiwan; 4 Division of Nephrology, Department of Internal Medicine, School of Medicine, College of Medicine and Division of Nephrology, Department of Internal Medicine, Wan Fang Hospital, Taipei Medical University, Taipei, Taiwan

Correction to: *Scientific Reports* 10.1038/s41598-019-40757-6, published online 14 March 2019

In this Article, Figure 3 is a duplication of Figure 2. The correct Figure 3 appears below as Figure 1.

**Figure 1:**
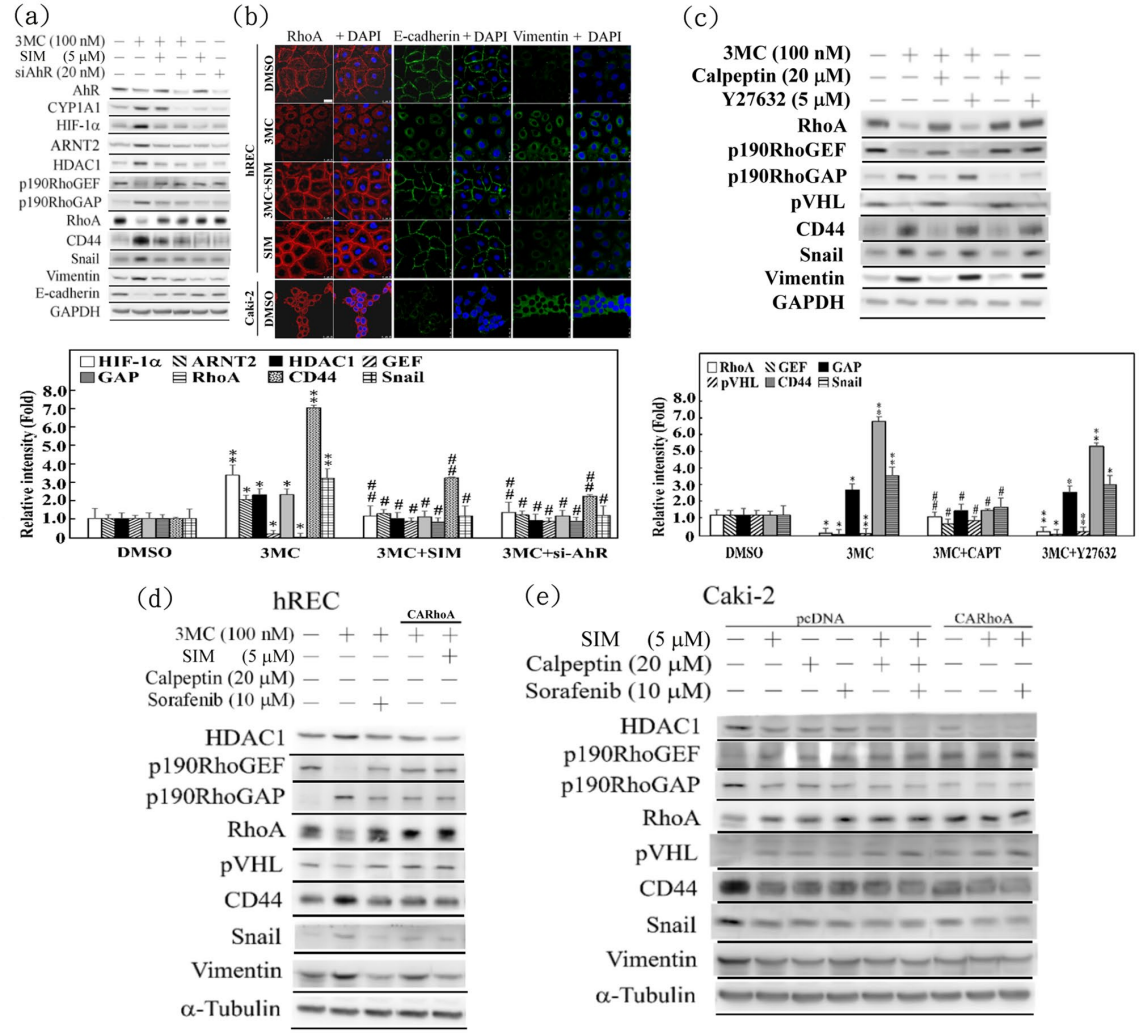
An essential role of RhoA in 3MC-mediated hREC carcinogenesis, in part, through an AhR-dependent mechanism. Cells were transfected with siAhR overnight or pretreated with simvastatin for 1 h, followed by a 3-h 3MC challenge, and the resulting cells were subjected to Western blot analysis (**a**) and immunofluorescence staining (**b**) for the indicated molecules. Scale bar = 25 μm. (**c**) Cells were pretreated with calpeptin and Y27632 (a ROCK inhibitor) for 1 h, followed by 3-h 3MC treatment, and the resulting cell lysates were analyzed by Western blots, with GAPDH used as an internal control. (**d**) hRECs were transfected with CARhoA overnight, and pretreated with calpeptin for 1 h or sorafenib for 5 h, followed by 3MC challenge for 3 h. Western blot analysis of the indicated proteins were carried out to compare the effects of CARhoA overexpression, simvastatin and calpeptin with sorafenib in hREC carcinogenesis caused by 3MC. (**e**) Caki-2 cells were transfected with empty or CARhoA overexpression vector overnight, followed by 1 h of simvastatin and calpeptin, or 5 h of sorafenib treatment. Western blot analysis of the EMT and hypoxia markers was carried out to evaluate the synergistic effects of the combination treatment of simvastatin, CARhoA overexpression (or calpeptin), and sorafenib in alleviating RCC progression. The bar chart and Table S3 show the normalized intensity of each protein band obtained using GAPDH or *α*-tubulin in Western blots. (*P < 0.05 and **P < 0.01 vs. control; ^#^P < 0.05 and ^##^P < 0.01 vs. 3MC treatment alone). The gels have been run in the same experimental conditions and the cropped blots were shown. The entire gel pictures were shown in the Supplemental Fig. 3.

