# Supplementary material for: Simvastatin reduces the carcinogenic effect of 3-methylcholanthrene in renal epithelial cells through histone deacetylase 1 inhibition and RhoA reactivation
Source: Sci Rep. 2019 Mar 14;9:4606. doi: 10.1038/s41598-019-40757-6 (PMC6418087; doi:10.1038/s41598-019-40757-6)

Supplementary Information

Simvastatin reduces the carcinogenic effect of 3-methylcholanthrene in renal epithelial cells through histone deacetylase 1 inhibition and RhoA reactivation

Chih-Cheng Chang,[1] Kuo-How Huang,[2] Sung-Po Hsu,[1] Yuan-Chii G. Lee,[3] Yuh-Mou Sue,[4] and Shu-Hui Juan[1,*]

[1] Department of Physiology, School of Medicine, College of Medicine, Taipei Medical University, Taipei, Taiwan

[2] National Taiwan University Hospital; Department of Urology, College of Medicine, National Taiwan University; and National Taiwan University Hospital, Taipei, Taiwan

[3] Graduate Institute of Biomedical Informatics, College of Medical Science and Technology, Taipei Medical University, Taipei, Taiwan

[4] Division of Nephrology, Department of Internal Medicine, School of Medicine, College of Medicine and Division of Nephrology, Department of Internal Medicine, Wan Fang Hospital, Taipei Medical University, Taipei, Taiwan.

Corresponding author:

Shu-Hui Juan, Ph.D.

Department of Physiology

Graduate Institute of Medical Sciences, Taipei Medical University

250 Wu-Hsing Street, Taipei 110, Taiwan

e-mail: juansh@tmu.edu.tw   Tel: 886-2-27361661

Running title: Simvastatin inhibits 3MC-mediated carcinogenesis through RhoA reactivation

Keywords: aryl-hydrocarbon receptor, 3-methylcholanthrene, simvastatin, calpeptin, renal cell carcinoma

Supplementary data

Fig. S1

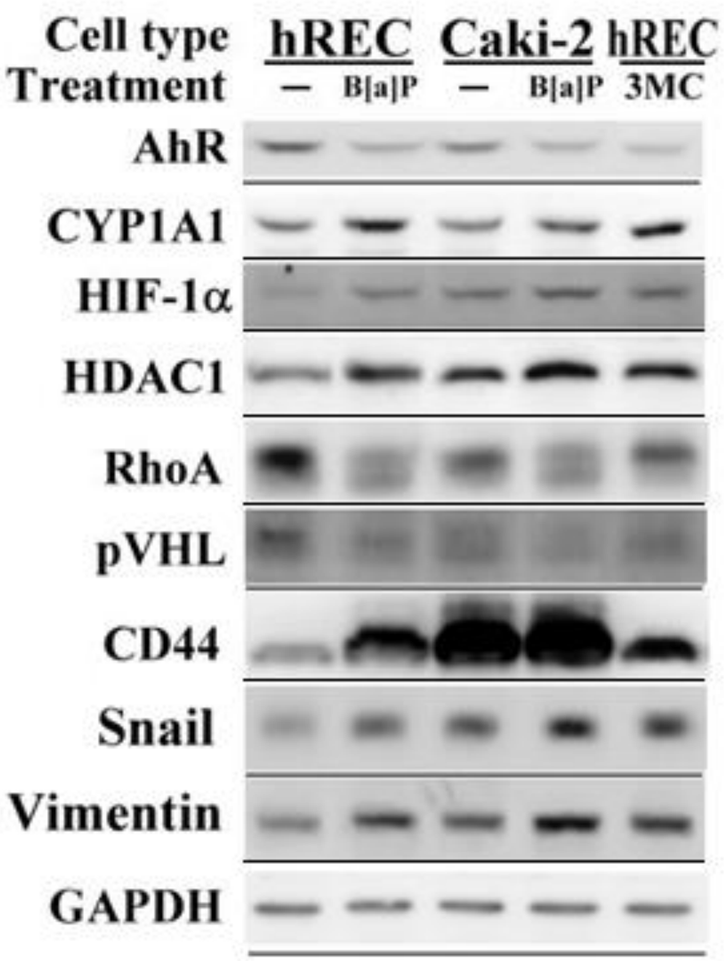

protein	Mean value of relative intensity ± SD				
	hREC			Caki-2	
	DMSO	B[a]P	3MC	DMSO	B[a]P
**AhR**	1.00±0.11	0.33±0.06*	0.22±0.17*	1.00±0.13	0.37±0.06*
**CYP1A1**	1.00±0.10	2.72±0.11*	2.81±0.16*	1.00±0.11	1.46±0.12*
**HIF-1α**	1.00±0.09	1.51±0.14*	1.57±0.12*	1.00±0.10	1.43±0.15*
**HDAC1**	1.00±0.11	2.52±0.18**	2.43±0.18**	1.00±0.08	3.22±0.18**
**RhoA**	1.00±0.12	0.10±0.12*	0.27±0.11*	1.00±0.05	0.34±0.14*
**pVHL**	1.00±0.14	0.24±0.12*	0.37±0.04*	1.00±0.09	0.37±0.13*
**CD44**	1.00±0.09	5.62±0.40*	5.28±0.35*	1.00±0.13	2.21±0.16**
**Snail**	1.00±0.09	2.02±0.19*	2.89±0.16*	1.00±0.13	2.47±0.15**
**Vimentin**	1.00±0.09	2.54±0.17*	2.13±0.17*	1.00±0.13	2.14±0.16**

Fig. S1. Effect of benzo(a)pyrene exposure on EMT in renal epithelial cells.
Indicated cells were exposed to 1 μM benzo(a)pyrene (B[a]P) or 100 nM 3MC for 3 h and Western blot analysis was performed by using Cyp1A1 as a positive control and GAPDH as a loading control. n = 3 in each group, $^{*}p \leqq 0.05$ and $^{**}p \leqq 0.01$ versus DMSO group.

Table S1: Quantification for Figure 1

Fig. 1(A)

protein	Mean value of relative intensity ± SD			
	hREC		Caki-2	
	DMSO	3MC	DMSO	3MC
**AhR**	1.00±0.11	0.45±0.12**	0.62±0.07*	0.31±0.11*
**CYP1A1**	1.00±0.08	1.89±0.09*	1.42±0.11*	2.14±0.06*
**Vimentin**	1.00±0.04	1.31±0.03*	1.45±0.12*	2.72±0.20*

protein	Mean value of relative intensity ± SD			
	ACHN		786-o	
	DMSO	3MC	DMSO	3MC
**AhR**	0.68±0.06*	0.32±0.10*	0.79±0.05*	0.28±0.15**
**CYP1A1**	0.87±0.12	1.97±0.06**	0.43±0.05*	1.85±0.08**
**Vimentin**	1.38±0.10*	2.13±0.08*	1.39±0.12*	2.24±0.08*

n = 3 in each group, $^{*}p \leqq 0.05$ and $^{**}p \leqq 0.01$ versus DMSO group.

Fig. 1(C)

protein	Mean value of relative intensity ± SD				
	DMSO	3MC	3MC+Digoxin	DFO	Mg132
**Acetyl-H3**	1.00±0.11	0.37±0.12**	1.02±0.07$^{\#}$	0.51±0.11*	1.03±0.11
**Histone H3**	1.00±0.09	1.05±0.12	1.10±0.14	0.92±0.17	1.01±0.13
**Vimentin**	1.00±0.10	4.21±0.33*	1.05±0.11$^{\#\#}$	3.97±0.29*	1.07±0.14

n = 3 in each group, $^{*}p \leqq 0.05$ and $^{**}p \leqq 0.01$ versus DMSO group, $^{\#}p \leqq 0.05$, and $^{\#\#}p \leqq 0.01$ versus 3MC treated group.

Table S2: Quantification for Figure 2

Fig. 2(A)

protein	Mean value of relative intensity ± SD				
	hREC			Caki-2	
	DMSO+ scramble	3MC+ scramble	3MC+ siHDAC1	scramble	siHDAC1
Acetyl-H3	1.00±0.08	0.28±0.09**	0.92±0.09#	0.14±0.08*	0.43±0.07+
Histone H3	1.00±0.12	0.92±0.10	1.02±0.11	1.08±0.12	0.98±0.06
Vimentin	1.00±0.10	2.15±0.20*	1.13±0.08#	2.11±0.16*	1.35±0.10+

n = 3 in each group, *$p \leqq 0.05$ and **$p \leqq 0.01$ versus DMSO+scramble group, #$p \leqq 0.05$, and ##$p \leqq 0.01$ versus 3MC+scramble treated group, +$p \leqq 0.05$ versus scramble treated caki-2 group.

Fig. 2(B)

protein	Mean value of relative intensity ± SD			
	DMSO	3MC	3MC+ SIM	3MC+ SAHA
t-HIF-1α	1.00±0.02	2.01±0.18*	1.12±0.10#	1.14±0.07#
Vimentin	1.00±0.12	2.61±0.16**	1.43±0.07#	1.39±0.05#

n = 3 in each group, *$p \leqq 0.05$ and **$p \leqq 0.01$ versus DMSO group, #$p \leqq 0.05$, and ##$p \leqq 0.01$ versus 3MC treated group.

Fig. 2(D)

protein	Mean value of relative intensity ± SD				
	DMSO	3MC	3MC+ SIM	3MC+ TSA	3MC+ TSA+iTSA
HDAC1	1.00±0.06	2.02±0.08*	1.19±0.07#	1.21±0.11#	2.15±0.14
GEF	1.00±0.12	0.74±0.05*	0.93±0.11#	0.90±0.08#	0.78±0.08
GAP	1.00±0.10	1.33±0.06*	1.04±0.13#	1.08±0.06#	1.39±0.05
RhoA	1.00±0.08	0.11±0.08*	0.94±0.11#	0.90±0.07#	0.23±0.08
pVHL	1.00±0.07	0.21±0.10*	0.89±0.09#	0.92±0.04#	0.29±0.12
CD44	1.00±0.13	3.42±0.25*	1.22±0.07#	1.10±0.12#	3.04±0.20
Snail	1.00±0.09	1.64±0.12*	1.13±0.04#	1.06±0.11#	1.55±0.13
Vimentin	1.00±0.03	2.16±0.17**	1.13±0.12#	1.05±0.05#	1.68±0.20

n = 3 in each group, *$p \leqq 0.05$ and **$p \leqq 0.01$ versus DMSO group, #$p \leqq 0.05$, and ##$p \leqq 0.01$ versus 3MC treated group.

Table S3: Quantification for Figure 3

Fig. 3(A)

protein	Mean value of relative intensity ± SD			
	DMSO	3MC	3MC+ SIM	3MC+ siAhR
**AhR**	1.00±0.04	0.31±0.06*	0.92±0.04#	0.18±0.12#
**CYP1A1**	1.00±0.06	2.02±0.05*	1.43±0.03#	0.35±0.10#
**E-cadherin**	1.00±0.08	0.26±0.07**	0.87±0.05#	0.93±0.11#
**Vimentin**	1.00±0.05	2.28±0.13**	1.32±0.12#	1.36±0.06##

n = 3 in each group, *$p \leqq 0.05$ and **$p \leqq 0.01$ versus DMSO group, #$p \leqq 0.05$, and ##$p \leqq 0.01$ versus 3MC treated group.

Fig. 3(C)

protein	Mean value of relative intensity ± SD			
	DMSO	3MC	3MC+ Calpeptin	3MC+ Y27632
**Vimentin**	1.00±0.11	3.12±0.25*	1.13±0.12#	4.23±0.36**

n = 3 in each group, *$p \leqq 0.05$ and **$p \leqq 0.01$ versus DMSO group, #$p \leqq 0.05$, and ##$p \leqq 0.01$ versus 3MC treated group.

Figure 3(D)

protein	Mean value of relative intensity ± SD				
	DMSO	3MC	3MC+ Sorafenib	3MC+ CARhoA	3MC+ CARhoA+SIM
**HDAC1**	1.00±0.05	1.53±0.08*	1.36±0.08#	1.27±0.10#	1.15±0.04#
**GEF**	1.00±0.08	0.16±0.07*	0.58±0.10#	0.76±0.07#	0.82±0.05#
**GAP**	1.00±0.11	2.78±0.26*	1.44±0.11#	1.47±0.05#	1.41±0.07#
**RhoA**	1.00±0.09	0.27±0.09*	0.87±0.12#	3.22±0.25#	3.13±0.17#
**pVHL**	1.00±0.06	0.35±0.11*	0.84±0.08#	0.91±0.04#	0.89±0.05#
**CD44**	1.00±0.12	3.55±0.32*	1.32±0.06#	1.27±0.08#	1.39±0.10#
**Snail**	1.00±0.04	1.54±0.12*	1.01±0.11#	1.03±0.06#	0.96±0.09#
**Vimentin**	1.00±0.05	2.76±0.19*	1.23±0.12#	1.49±0.09#	1.28±0.11#

n = 3 in each group, *$p \leqq 0.05$ versus DMSO group, #$p \leqq 0.05$ versus 3MC treated group, +$p \leqq$ 0.05 versus scramble treated DNRhoA group.

Fig. 3(E)

protein	Mean value of relative intensity ± SD					
	DMSO	SIM	Calpeptin	Sorafenib	SIM+ Calpeptin	SIM+Calpeptin +Sorafenib
HDAC1	1.00±0.03	0.43±0.12*	0.35±0.06*	0.38±0.12*	0.22±0.07*	0.17±0.11*
GEF	1.00±0.10	1.24±0.10*	1.26±0.05*	1.29±0.05*	1.31±0.06*	1.39±0.09*
GAP	1.00±0.10	0.83±0.13*	0.82±0.03*	0.79±0.04*	0.71±0.03*	0.74±0.07*
RhoA	1.00±0.12	1.46±0.12*	1.34±0.02*	1.36±0.12*	1.35±0.05*	1.31±0.08*
pVHL	1.00±0.02	1.18±0.07*	1.19±0.08*	1.22±0.11*	1.32±0.02*	1.37±0.04*
CD44	1.00±0.03	0.32±0.08*	0.28±0.04*	0.33±0.07*	0.31±0.01*	0.28±0.03*
Snail	1.00±0.04	0.64±0.06*	0.51±0.09*	0.55±0.04*	0.56±0.12*	0.49±0.06*
Vimentin	1.00±0.03	0.43±0.03*	0.36±0.07*	0.41±0.09*	0.34±0.06*	0.33±0.06*

protein	Mean value of relative intensity ± SD		
	CARhoA	CARhoA+ SIM	CARhoA+ Sorafenib
HDAC1	0.28±0.08*	0.32±0.13*	0.16±0.09*
GEF	1.43±0.12*	1.36±0.20*	1.88±0.12*
GAP	0.65±0.11*	0.62±0.11*	0.66±0.05*
RhoA	2.53±0.22**	2.44±0.24**	2.32±0.27**
pVHL	1.15±0.13	1.31±0.14*	1.33±0.18*
CD44	0.32±0.04*	0.22±0.12*	0.14±0.12*

n = 3 in each group, *$p \leqq 0.05$ and **$p \leqq 0.01$ versus DMSO group.

Table S4: Quantification for Figure 4

Fig. 4(A)

IP	IB	Mean value of relative intensity ± SD			
		Ctrl	3-MC	3-MC+CAPT	3-MC+SIM
AhR	HIF-1α	1.00±0.11	2.97±0.12**	1.14±0.14##	1.11±0.07##
	ARNT2	1.00±0.12	5.18±0.33**	1.20±0.09##	1.22±0.11##
	HDAC1	1.00±0.10	4.15±0.15**	1.20±0.08##	1.12±0.09##
HIF-1α	AhR	1.00±0.07	2.15±0.09*	1.17±0.04#	1.12±0.04#
	ARNT2	1.00±0.10	3.82±0.26**	1.30±0.05##	1.30±0.03##
	HDAC1	1.00±0.10	1.56±0.13**	1.05±0.09#	1.04±0.07#
ARNT2	AhR	1.00±0.11	1.82±0.12*	1.07±0.09#	1.07±0.06#
	HIF-1α	1.00±0.11	2.46±0.15*	1.16±0.07#	1.07±0.05#
	HDAC1	1.00±0.06	4.54±0.32**	1.36±0.12##	1.40±0.12##

n= 3 in each group, *p≦0.05 and **p≦0.01 versus control group, #p≦0.05, ##p≦0.01 versus 3-MC treated group.

Fig. 4(B)

Gene	Element	Mean value of relative intensity ± SD			
		Ctrl	3-MC	3-MC+CAPT	3-MC+SIM
AhR	DRE	1.00±0.09	4.72±0.36**	1.16±0.06##	1.11±0.05##
	HRE	1.00±0.09	3.11±0.22**	1.19±0.06##	1.15±0.06##
HIF-1α	DRE	1.00±0.10	3.64±0.31*	1.23±0.11##	1.22±0.08##
	HRE	1.00±0.09	3.54±0.34*	1.16±0.04#	1.09±0.11#
ARNT2	DRE	1.00±0.08	2.68±0.23*	1.11±0.09##	1.06±0.11##
	HRE	1.00±0.09	1.85±0.09*	1.09±0.13#	1.12±0.09#
HDAC1	DRE	1.00±0.09	2.83±0.18*	1.25±0.09##	1.27±0.11##
	HRE	1.00±0.08	1.92±0.15*	1.13±0.06#	1.10±0.06#

n= 3 in each group, **p*≦0.05 and ***p*≦0.01 versus control group, #*p*≦0.05, ##*p*≦0.01 versus 3-MC treated group.

Table S5: Quantification for Figure 6(C)

**protein**	**Mean value of relative intensity ± SD**			
	**DMSO**	**3MC**	**3MC+ SIM**	**SIM**
**CYP1A1**	1.00±0.07	2.23±0.16*	1.76±0.24	1.12±0.12#
**Vimentin**	1.00±0.03	3.08±0.23*	1.22±0.15#	1.10±0.09#
**E-cadherin**	1.00±0.06	0.36±0.08*	0.90±0.11#	1.04±0.13#

n = 3 in each group, **p* ≦ 0.05 versus DMSO group, #*p* ≦ 0.05 versus 3MC treated group.

Supplementary Materials and Methods

Table S6: List of antibodies for immunoblot or immunofluorescence analysis.

Primary antibody	Supplier	Species	Type	Catalog No.	Dilution
LaminA/C(N-18)	Santa Cruz Biotechnology, Inc., Dallas, TX, USA	goat	polyclonal	sc-6215	1:500
p190RhoGEF (RGNEF, T-15)		goat	polyclonal	sc-161184	1:250
CYP1A1(A-9)		mouse	monoclonal	sc-393979	1:250
RhoA(26C4)		mouse	monoclonal	sc-418	1:1000
GAPDH(6C5)		mouse	monoclonal	sc-32233	1:2500
pVHL(FL-181)		rabbit	polyclonal	sc-5575	1:250
VE-cadherin(C-19)		goat	polyclonal	sc-6458	1:500
HDAC1(H-51)		rabbit	polyclonal	sc-7872	1:500
Acetyl-Histone H3 (AH3-120)		mouse	monoclonal	sc-56616	1:250
MMP2(H-76)		rabbit	polyclonal	sc-10736	1:250
Histone H3(E.960.2)	ThermoFisher Scientific, Inc., Waltham, MA, USA	rabbit	monoclonal	MA5-15150	1:1000
CD44(8E2)	Cell Signaling Technology, Denvers, MA, USA	mouse	monoclonal	5640S	1:1000
Snail	GeneTex, Inc., Irvine, CA, USA	rabbit	polyclonal	GTX121924	1:1000
HIF-1a		rabbit	polyclonal	GTX127309	1:1000
Vimentin		rabbit	polyclonal	GTX100619	1:1000
ARNT2(N2C1)		rabbit	polyclonal	GTX115314	1:1000
E-cadherin		rabbit	polyclonal	GTX100443	1:250
AhR	Enzo Life Sciences, Inc., Farmingdale, NY, USA	rabbit	polyclonal	BML-SA210 -0100	1:500
p190RhoGAP	BD Biosciences, SanJose, CA, USA	mouse	monoclonal	610149	1:1000
α-Tubulin	Sigma-Aldrich, Inc., St. Louis, MO, USA	mouse	monoclonal	T5168-.2ML	1:2500

Secondary antibody	Supplier	Catalog No.	Dilution
IP/WB Matrix (mouse) Optima E	Santa Cruz Biotechnology, Inc., Dallas, TX, USA	sc-45042	1:5000
IP/WB Matrix (rabbit) Optima E		sc-45043	1:5000
Anti-goat IgG (whole molecule)-Peroxidase (H+L)		A5420-1ML	1:2500
Anti-mouse IgG (whole molecule)-Peroxidase(H+L)		A9044-2ML	1:5000
Anti-rabbit IgG (whole molecule)-Peroxidase(H+L)		A0545-1ML	1:5000
Protein A/G PLUS-Agarose		sc-2003	1:5
Protein A agarose/Salmon Sperm DNA	MilliporeSigma, Burlinton, MA, USA	16-57	1:5
Fluorescein(FITC)-conjugated AffiniPure goat anti-rabbit IgG(H+L)	Jackson ImmunoResearch Laboratories, Inc., West Grove, PA, USA	111-095-144	1:200
Alexa Fluor® 488 AffiniPure Goat anti-mouse IgG(H+L)		115-545-003	1:200
DAPI	Biotium, Inc., Fremont, CA, USA	40043	1:1000

Table S7: siRNA sequences for AhR and HDAC1 knockdown.

Target gene	Product ID		Sequence (5′-3′)	Supplier
AhR	s1199	Sense:	ACGUGACACGUUCGGAGAATT	Ambion
		Antisense:	GCAUGAUAGUUUUCCGGCUTT	
	S1198	Sense:	GGCUCUUUCAAGAUAGUAATT	
		Antisense:	UUACUAUCUUGAAAGAGCCCT	
HDAC1	822	Sense:	CCGGUUAGGUUGCUUCAAUTT	MDBio, Inc.
		Antisense:	AUUGAAGCAACCUAACCGGTT	
	375	Sense:	AGUGCUGUGAAGCUUAAUATT	
		Antisense:	UAUUAAGCUUCACAGCACUTT	
	247	Sense:	GUUCUAUUCGCCCAGAUAATT	
		Antisense:	UUAUCUGGGCGAAUAGAACTT	
Negative control	29551	Antisense:	UUCUCCGAACGUGUCACGUTT	

Table S8: Primers for RT-/Q PCR analysis.

mer name		Primer sequence (5′-3′)	Product size (bps)
RhoA	Forward:	AAGGACCAGTTCCCAGAGGT	209
	Reverse:	TTCTGGGGTCCACTTTTCTG	
CD44	Forward:	CTGATCATCTTGGCATCCCTC	207
	Reverse:	CCATTTCCTGAGACTTGCTG	
KIM-1	Forward:	ACCTATCGGAAGGACACACG	210
	Reverse:	GACGGTTGGAACAGTTGTGA	
GAPDH	Forward:	ACCACAGTCCATGCCATCAC	434
	Reverse:	TCCACCACCCTGTTGCTGTA	

Table S9: Primer for ChIP assays.

Enhancer region on promoter (*RhoA*)		Primer sequence (5′-3′)	Product size (bps)
HRE	Forward:	AACTGAGGGGATTGTGCAGA	222
	Reverse:	GAGGGGAGTTCTCAGGCTTT	
DRE	Forward:	TGCCCAGGAGACTTAACACC	162
	Reverse:	CAGTCATGGCGGAGTCCTGG	

Immunofluorescence staining

Cells grown overnight on glass coverslips were pretreated 5 μM of simvastatin for 1 h, followed by 100 nM of 3MC for 3 h. The resulting cells were washed once with cold phosphate-buffered saline (PBS) and fixed for 10 min with 4% paraformaldehyde solution. Cells were then permeablized with 0.1% Triton X-100 and 0.05% Tween 20 in PBS at room temperature (RT). Coverslips were blocked with 10% goat serum at RT for 1h, then stained with (rhodaminephalloidin (ICN Immunobiologicals, Costa Mesa, CA, USA) in a 1:100 dilution or with anti-vinculin at 1:100 (Sigma-Aldrich) overnight at 4 °C followed by Texas red-conjugated goat anti-mouse (Jackson ImmunoResearch Laboratories, West Grove, PA, USA) at 1:100 for 60 min at room temperature.), and the indicated antibodies in a 1:100 dilution overnight at 4℃, followed by Alexa Fluor® 488 AffiniPure Goat anti-mouse or Fluorescein (FITC)-conjugated AffiniPure goat anti-rabbit (Jackson ImmunoResearch Laboratories, West Grove, PA) at 1:100 for 60 min at room temperature. Coverslips were then mounted on glass slides with anti-fade solution (Vector Laboratories, Burlingame, CA) diluted 1:1 with PBS. Images of cells were obtained with a DMI 6000B CS laser confocal microscope (Leica, Heidelberg, Germany) using an HCX PL APO l-blue 63x/1.40 to 0.60 NA oil-immersion objective lens. Images were acquired with a CM350 CCD camera (Applied Precision, Issaquah, WA) using the TCS SP5 confocal spectral microscope imaging system software (Leica).

Periodic acid solution staining

Paraffin embedded tissue sections were de-paraffinized and hydrated with xylene and with decreasing concentrations of ethanol, respectively. The sections were oxidized in 0.5% periodic acid solution for 5 min, followed by the addition with Schiff’s reagent for 15 min. After washing in tap water for 5 min, the tissue sections were counterstained with hematoxylin for 1 min, which were examined using light microscopy and assessed for glycogen and lipid deposition.

Supple. Fig. 1(A)

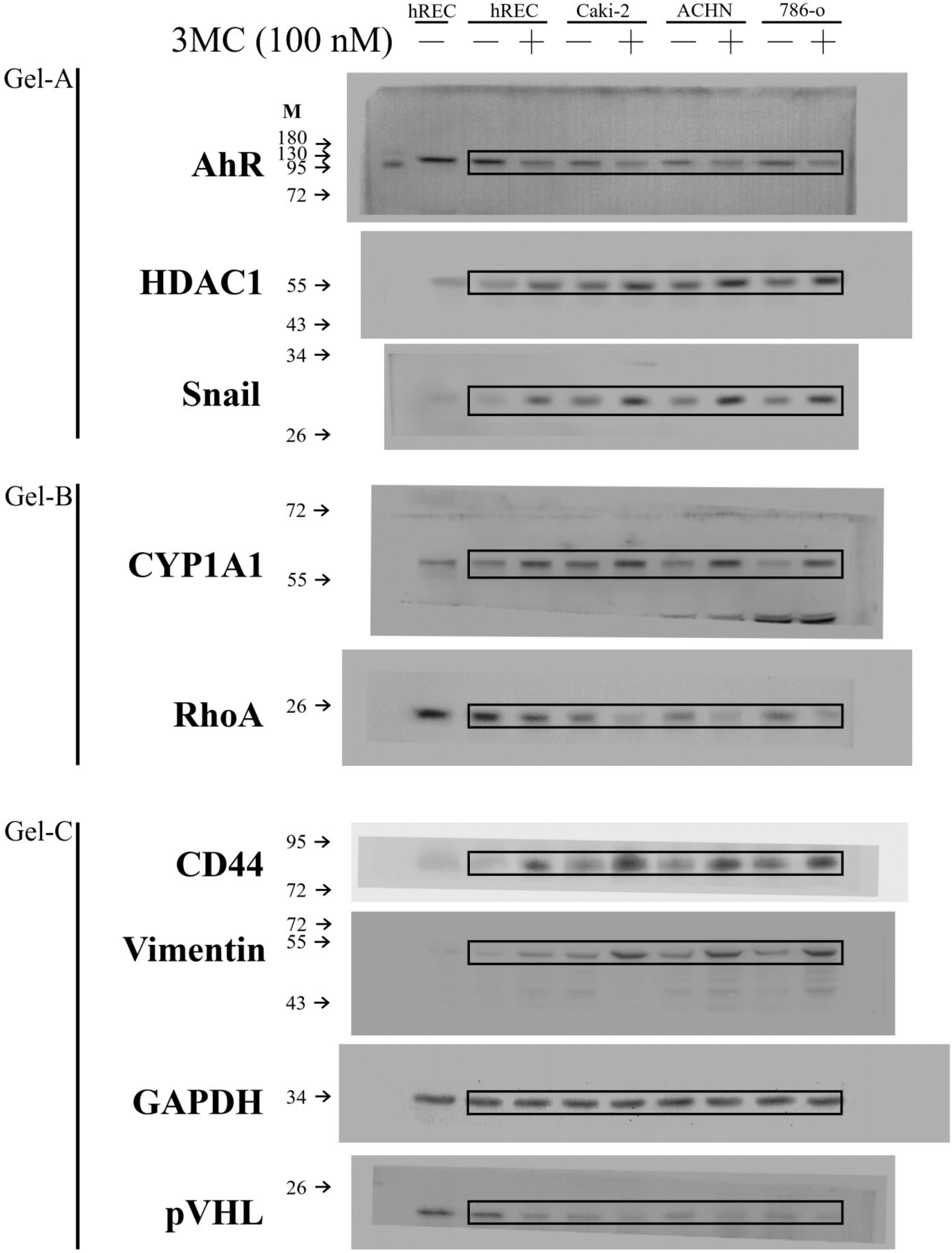

Supple. Fig. 1(C)

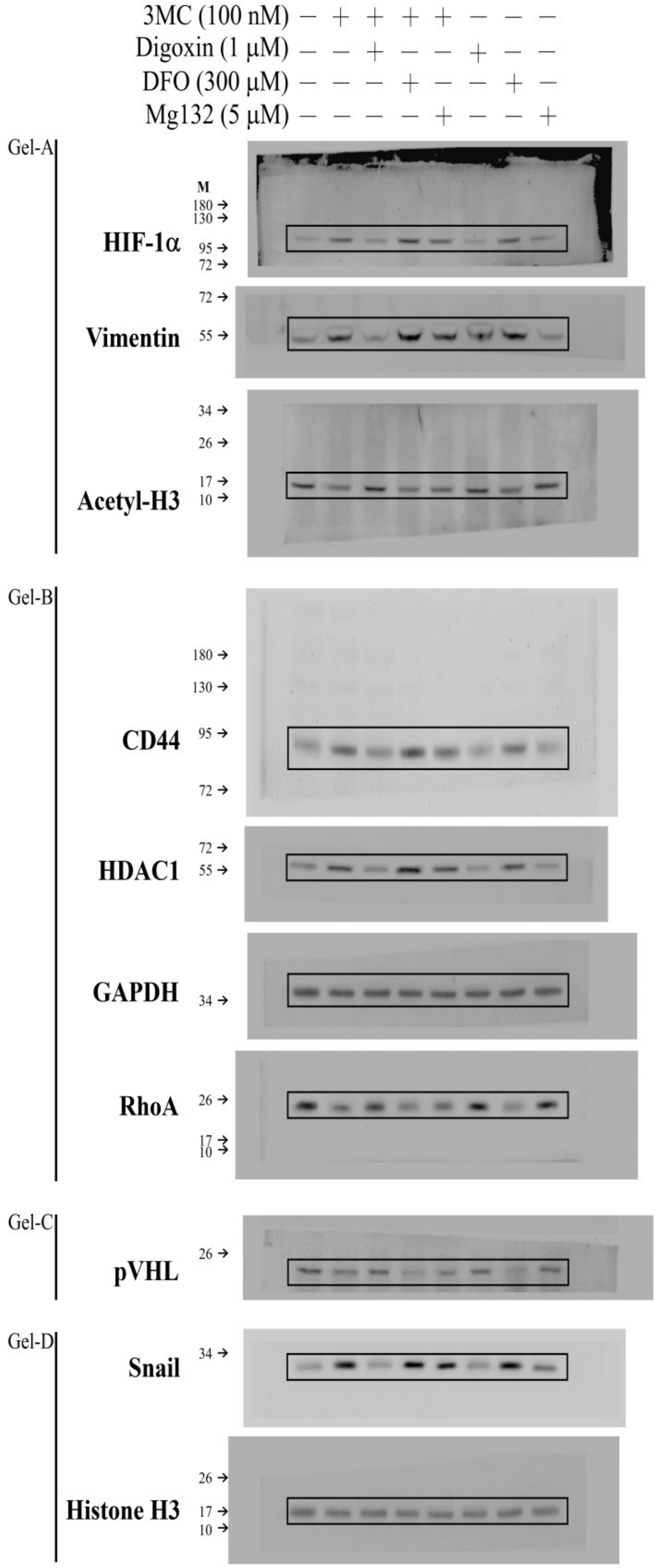

Supple. Fig. 2(A)

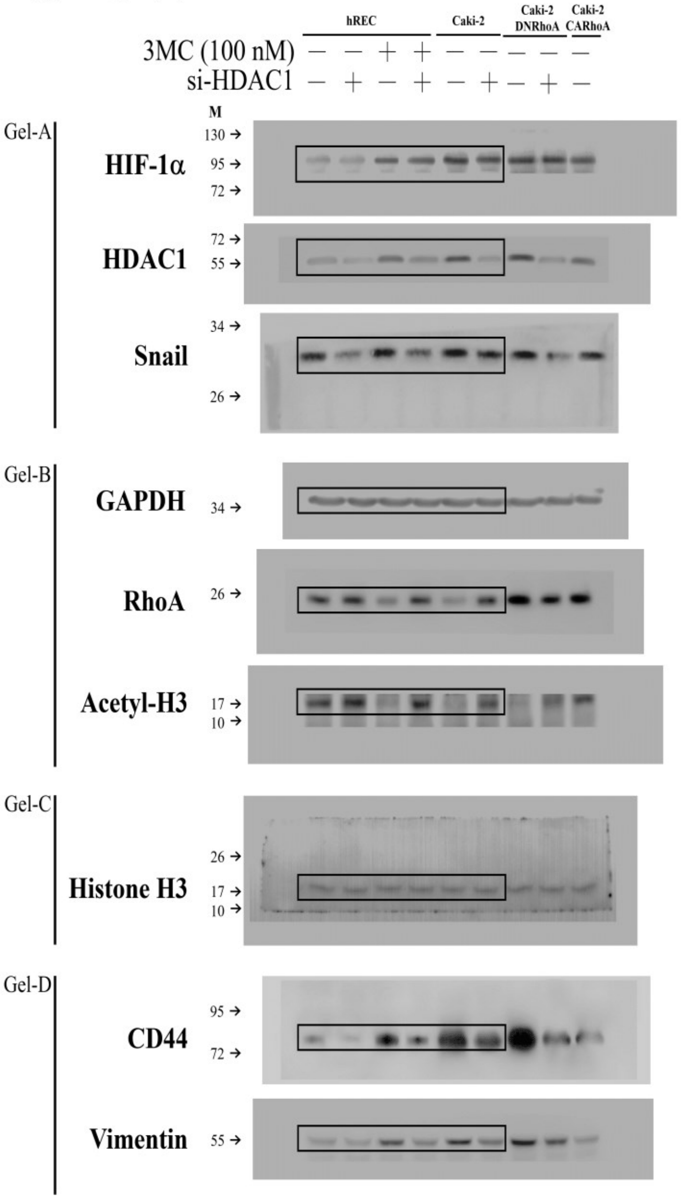

Supple. Fig. 2(B)

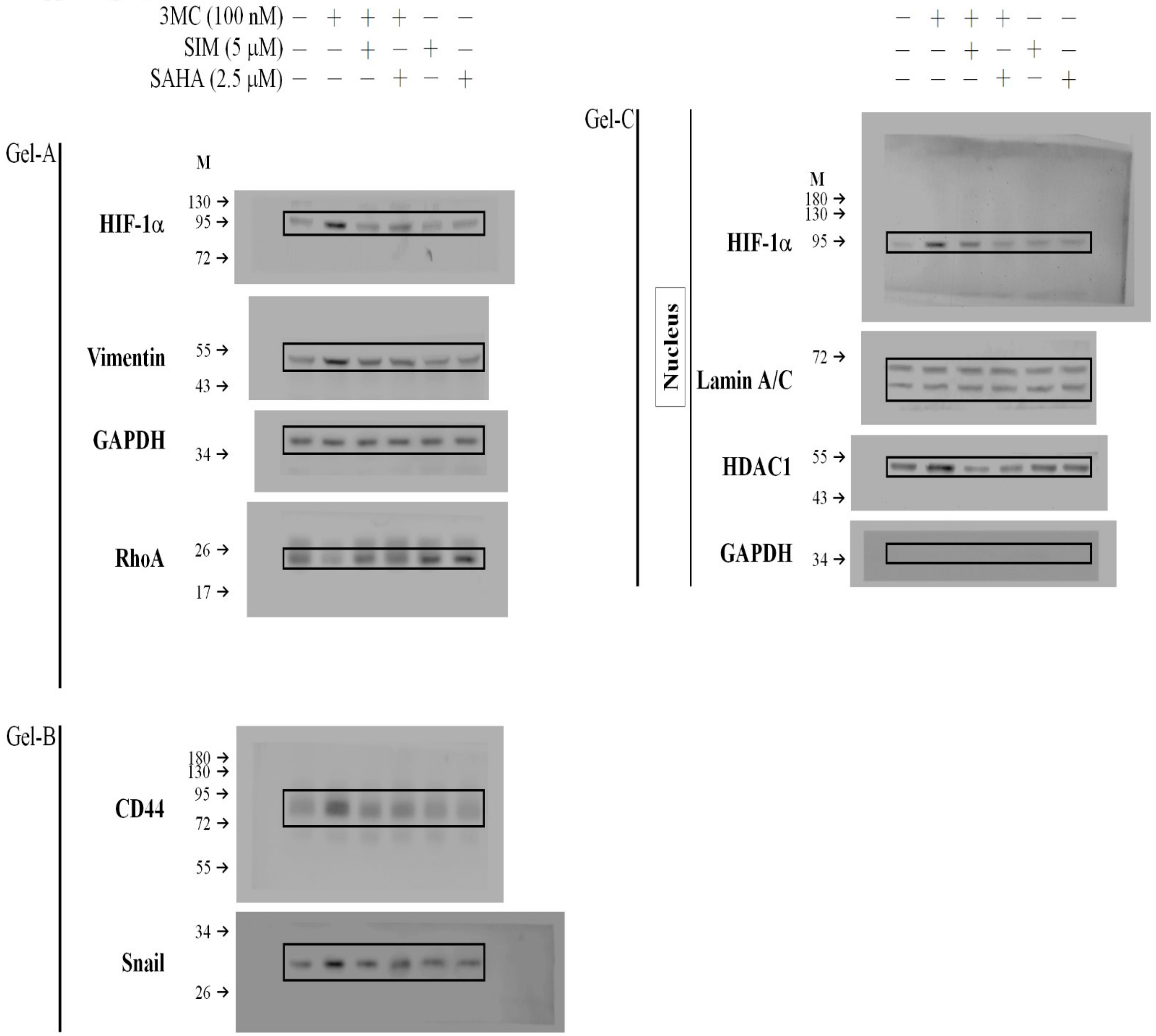

Supple. Fig. 2(C)

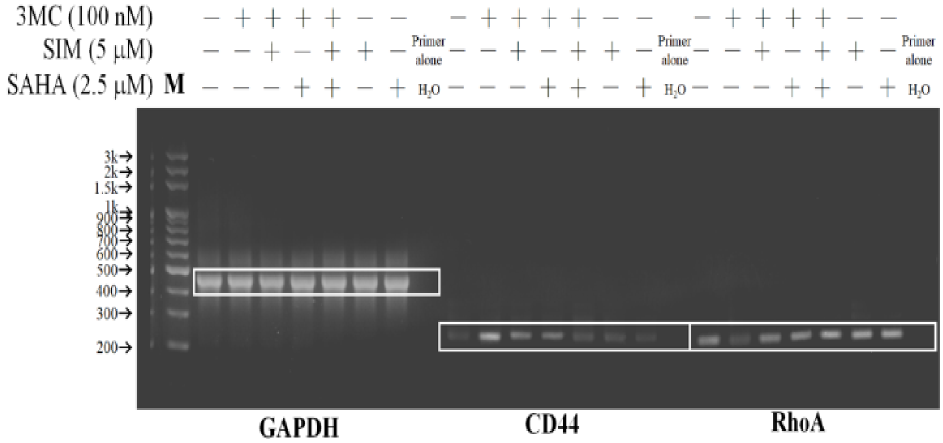

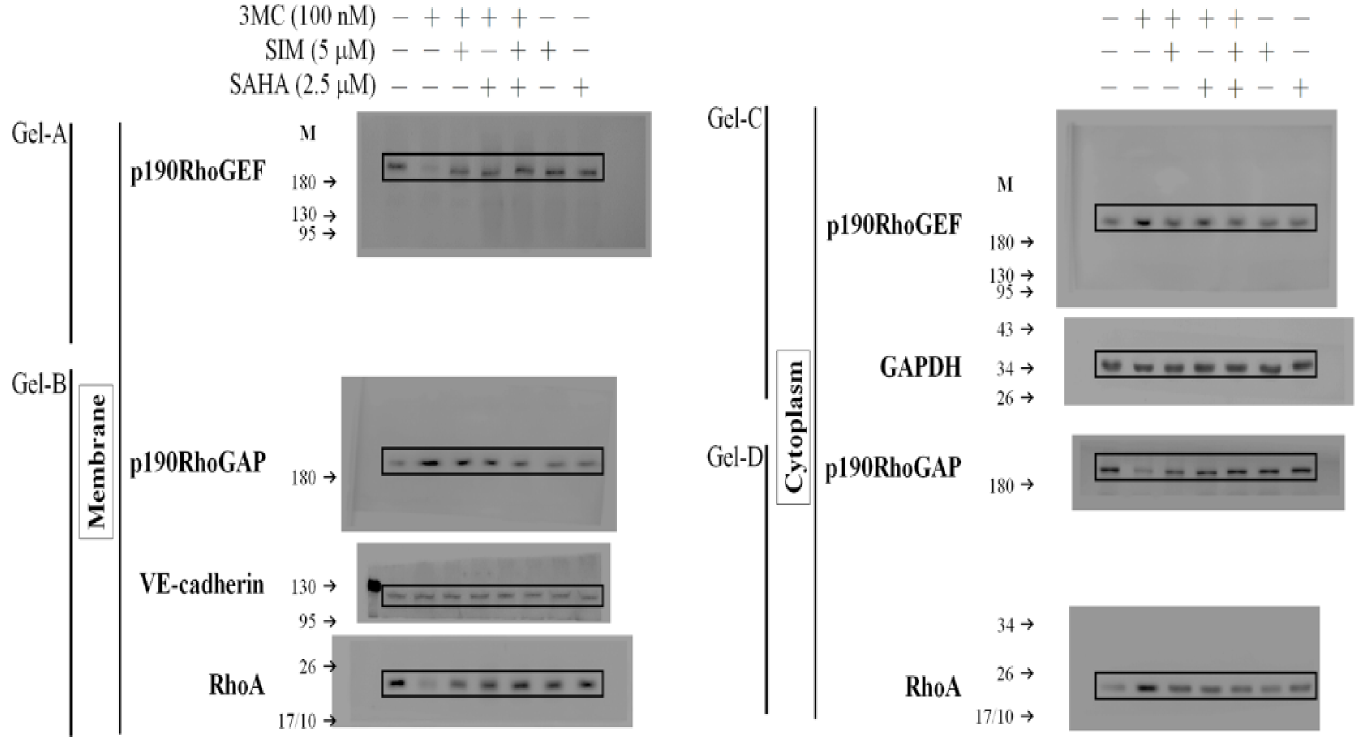

Supple. Fig. 2(D)

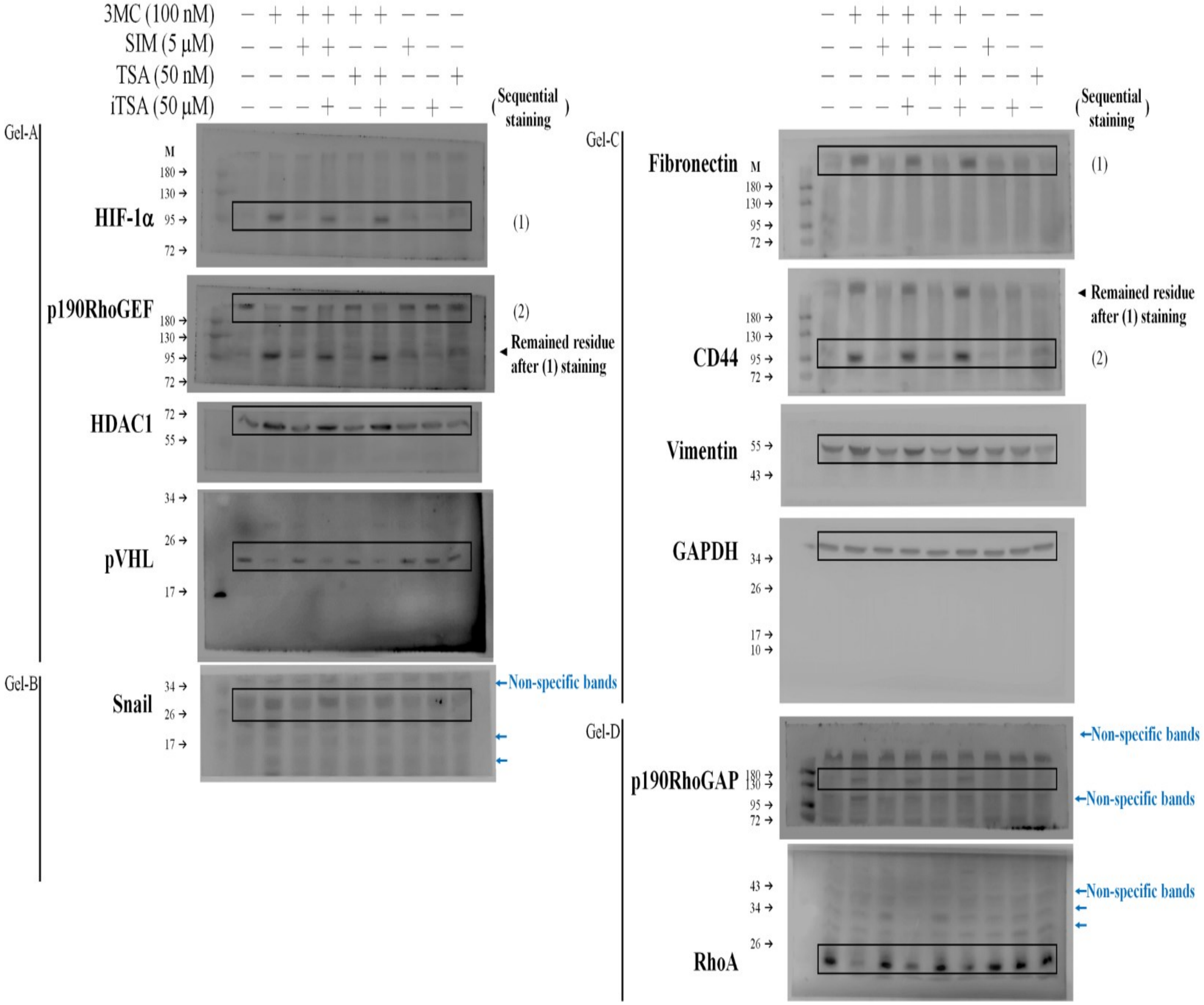

Supple. Fig. 2(E)

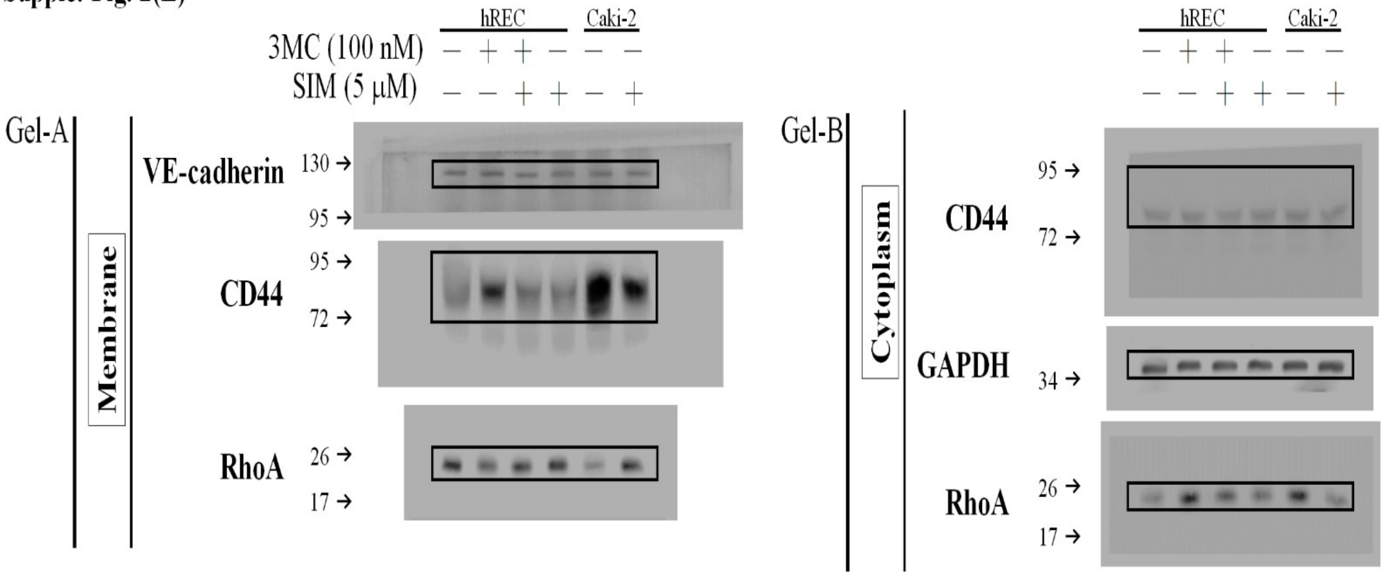

Supple. Fig. 3(A)

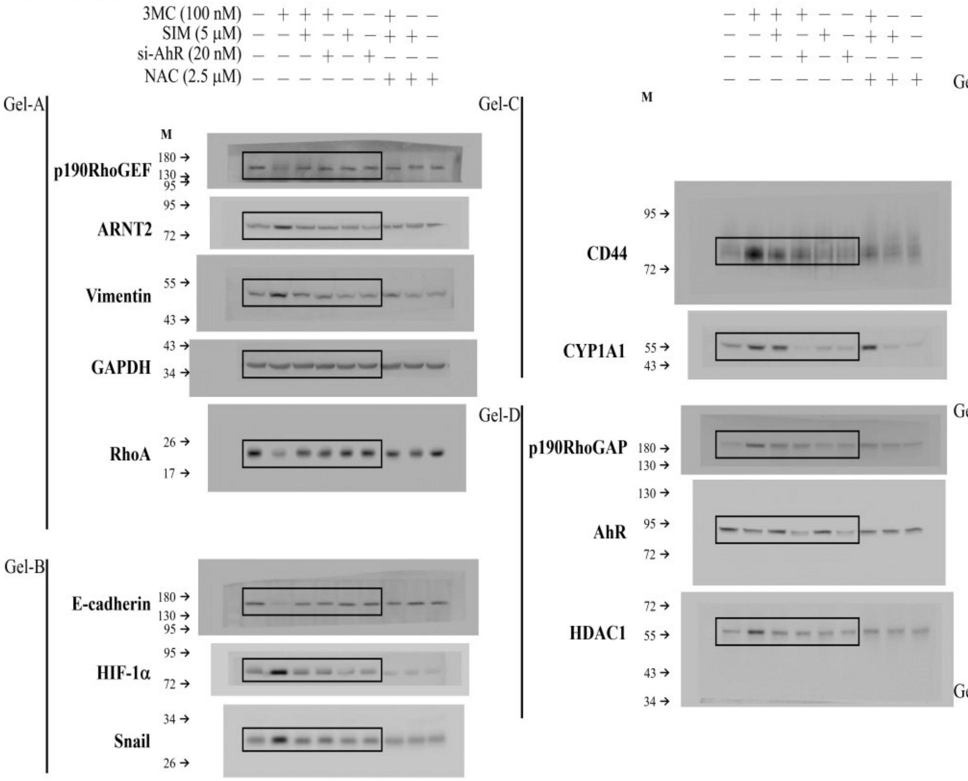

Supple. Fig. 3(C)

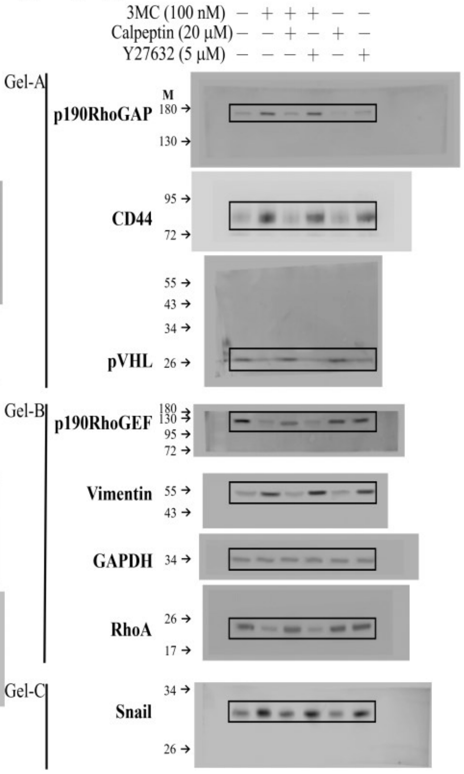

Supple. Fig. 3(D)

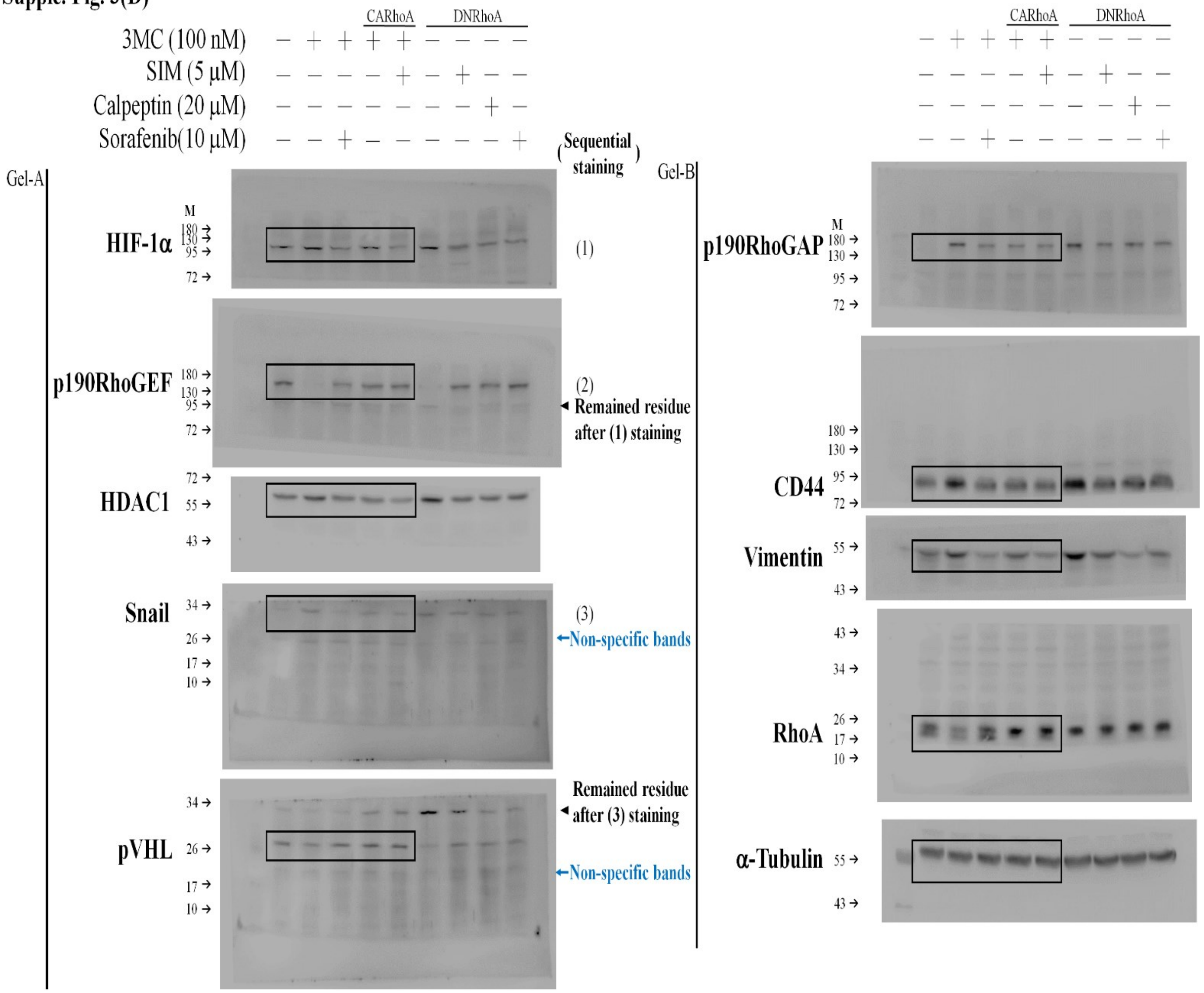

Supple. Fig. 3(E)

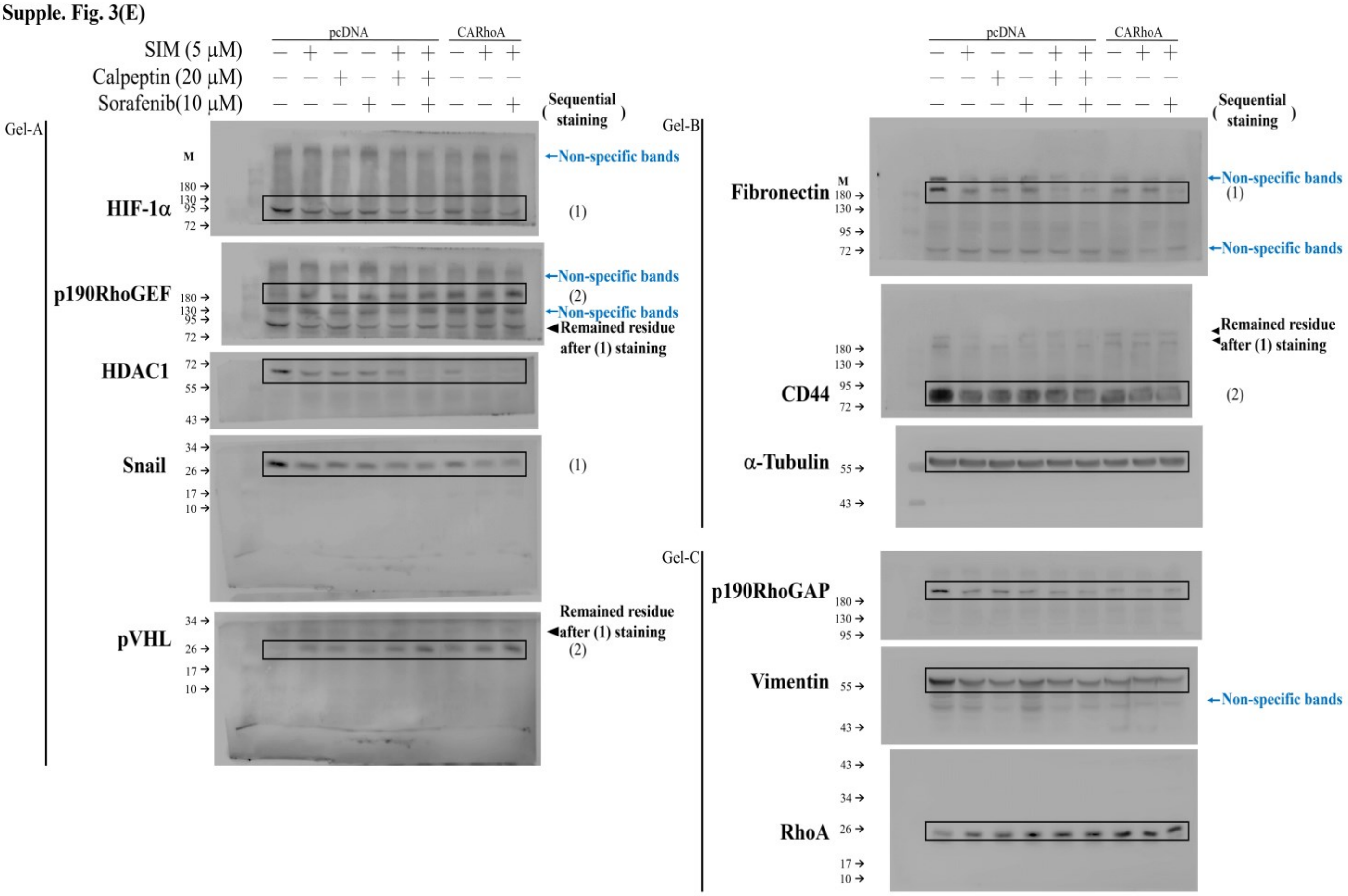

Supple. Fig. 4(A)

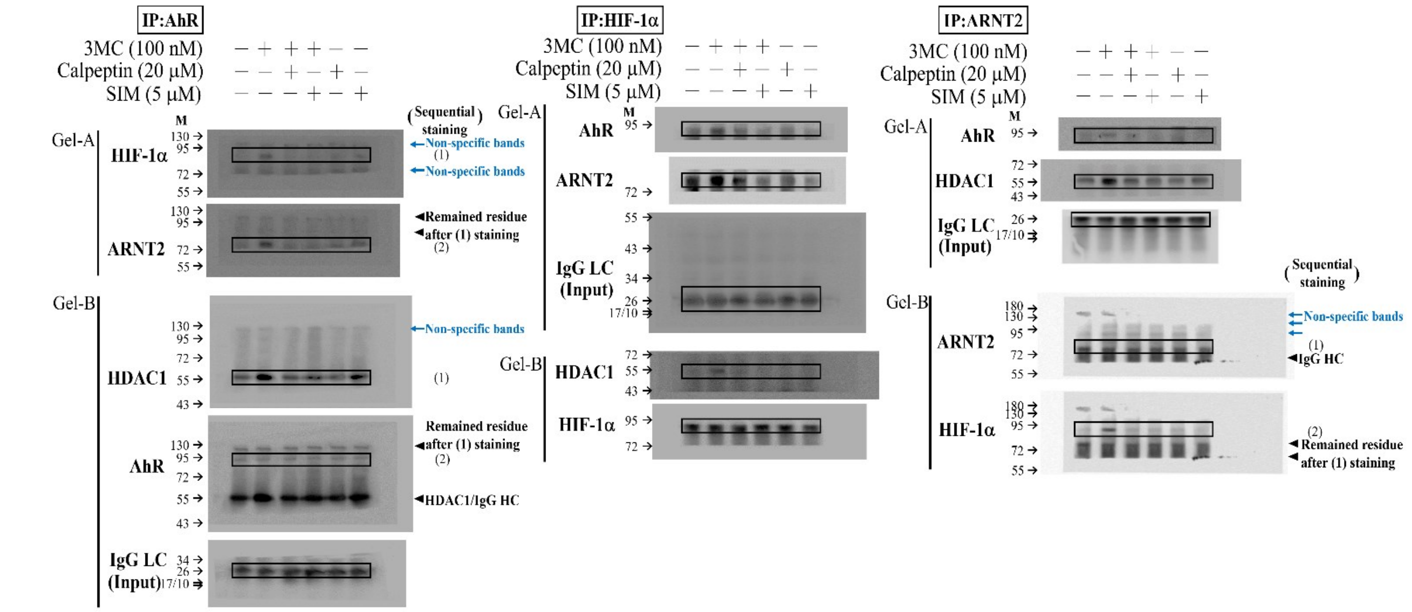

Supple. Fig. 4(B)

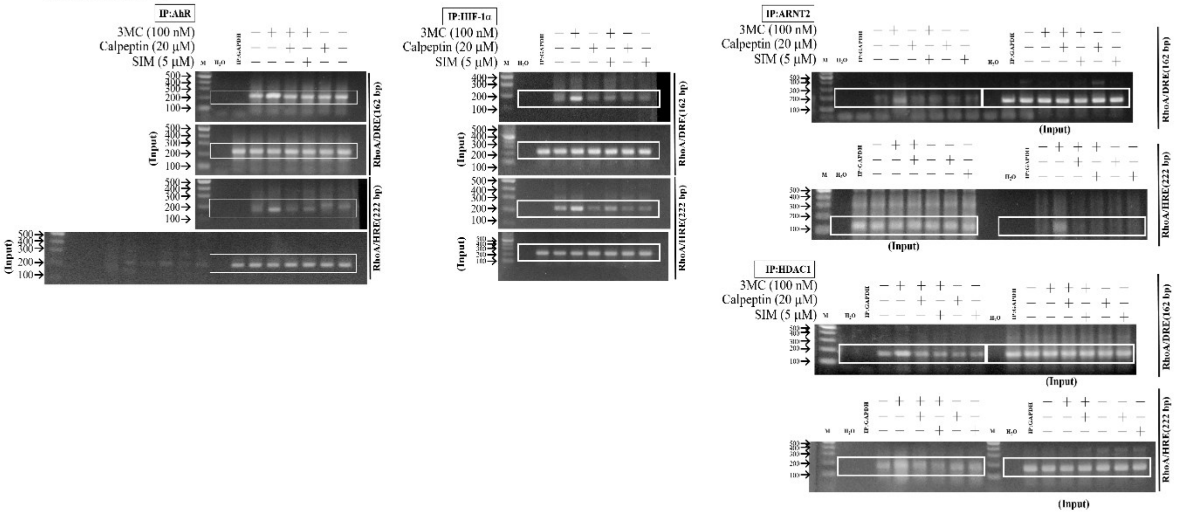

Supple. Fig. 5(B)

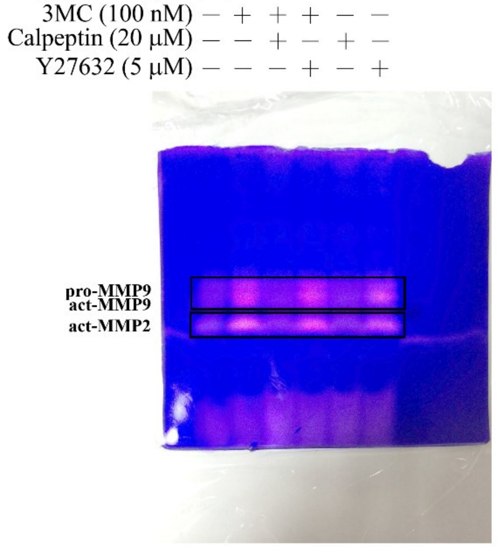

Supple. Fig. 6(C)

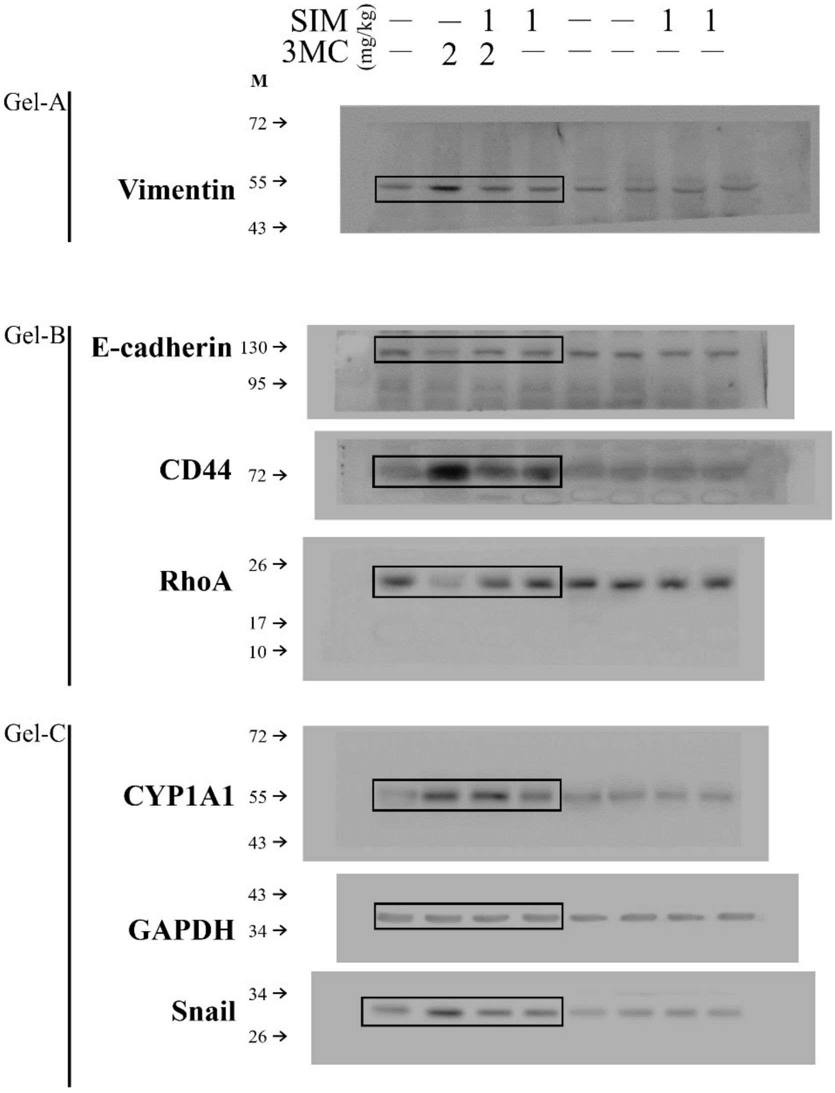

Supple. Fig. S1

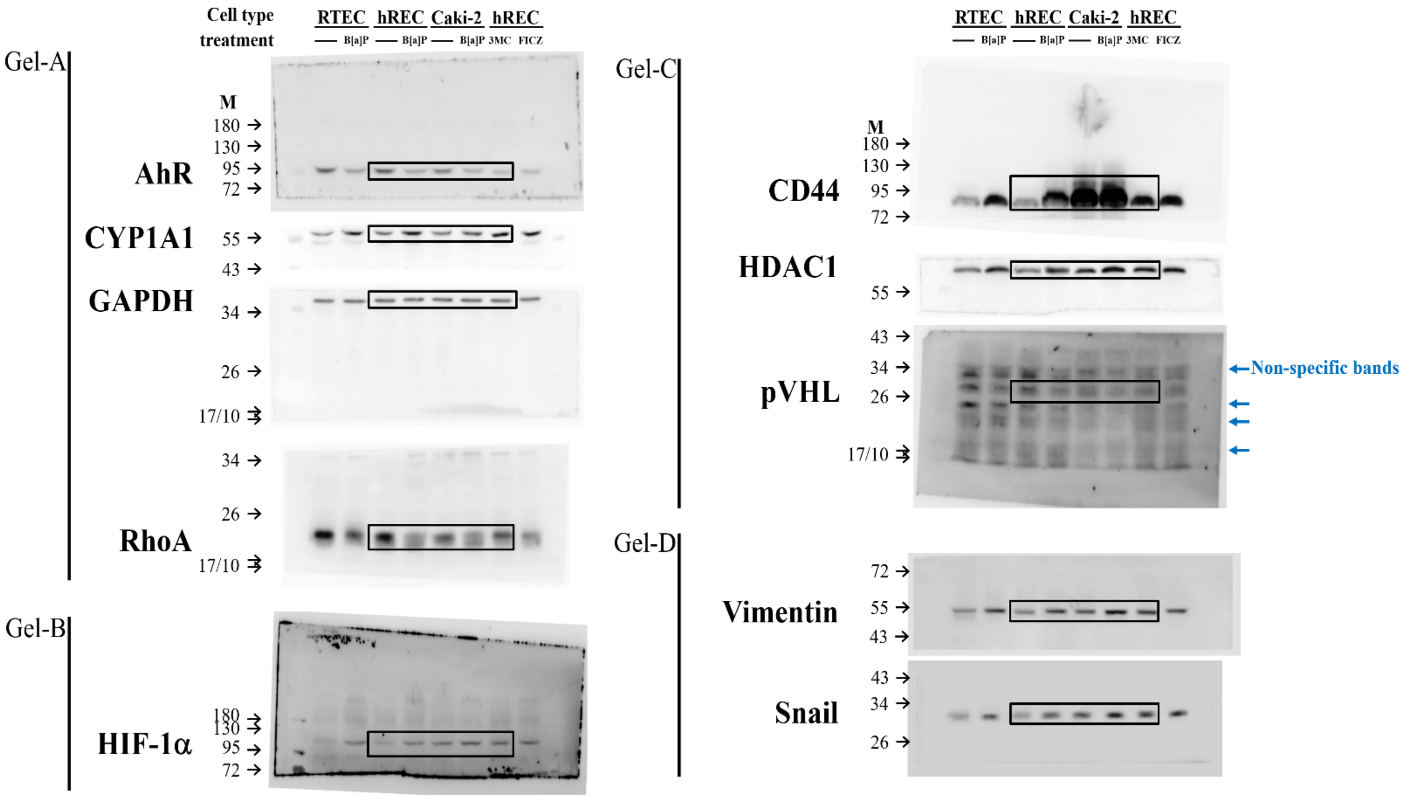

Supplement: Supplementary file 1 — Supplementary Information [file 41598_2019_40757_MOESM1_ESM.pdf]
