# Supplementary material for: Simvastatin reduces the carcinogenic effect of 3-methylcholanthrene in renal epithelial cells through histone deacetylase 1 inhibition and RhoA reactivation
Source: Sci Rep. 2019 Mar 14;9:4606. doi: 10.1038/s41598-019-40757-6 (PMC6418087; doi:10.1038/s41598-019-40757-6)
Supplement: Supplementary file 2 — Raw data [file 41598_2019_40757_MOESM2_ESM.pdf]

# Additional Review Materials

Simvastatin reduces the carcinogenic effect of 3-methylcholanthrene in renal epithelial cells through histone deacetylase 1 inhibition and RhoA reactivation

Chih-Cheng Chang,[1,*] Kuo-How Huang,[2,] Sung-Po Hsu,[1] Yuan-Chii G. Lee,[3] Yuh-Mou Sue,[4] and Shu-Hui Juan[1]

[1] Department of Physiology, School of Medicine, College of Medicine, Taipei Medical University, Taipei, Taiwan

[2] National Taiwan University Hospital; Department of Urology, College of Medicine, National Taiwan University; and National Taiwan University Hospital, Taipei, Taiwan

[3] Graduate Institute of Biomedical Informatics, College of Medical Science and Technology, Taipei Medical University, Taipei, Taiwan

[4] Division of Nephrology, Department of Internal Medicine, School of Medicine, College of Medicine and Division of Nephrology, Department of Internal Medicine, Wan Fang Hospital, Taipei Medical University, Taipei, Taiwan.

**Corresponding author:**

Shu-Hui Juan, Ph.D.

Department of Physiology

Graduate Institute of Medical Sciences, Taipei Medical University

250 Wu-Hsing Street, Taipei 110, Taiwan

e-mail: juansh@tmu.edu.tw Tel: 886-2-27361661

**Running title:** Simvastatin inhibits 3MC-mediated carcinogenesis through RhoA reactivation

Supple. Fig. 1(A)

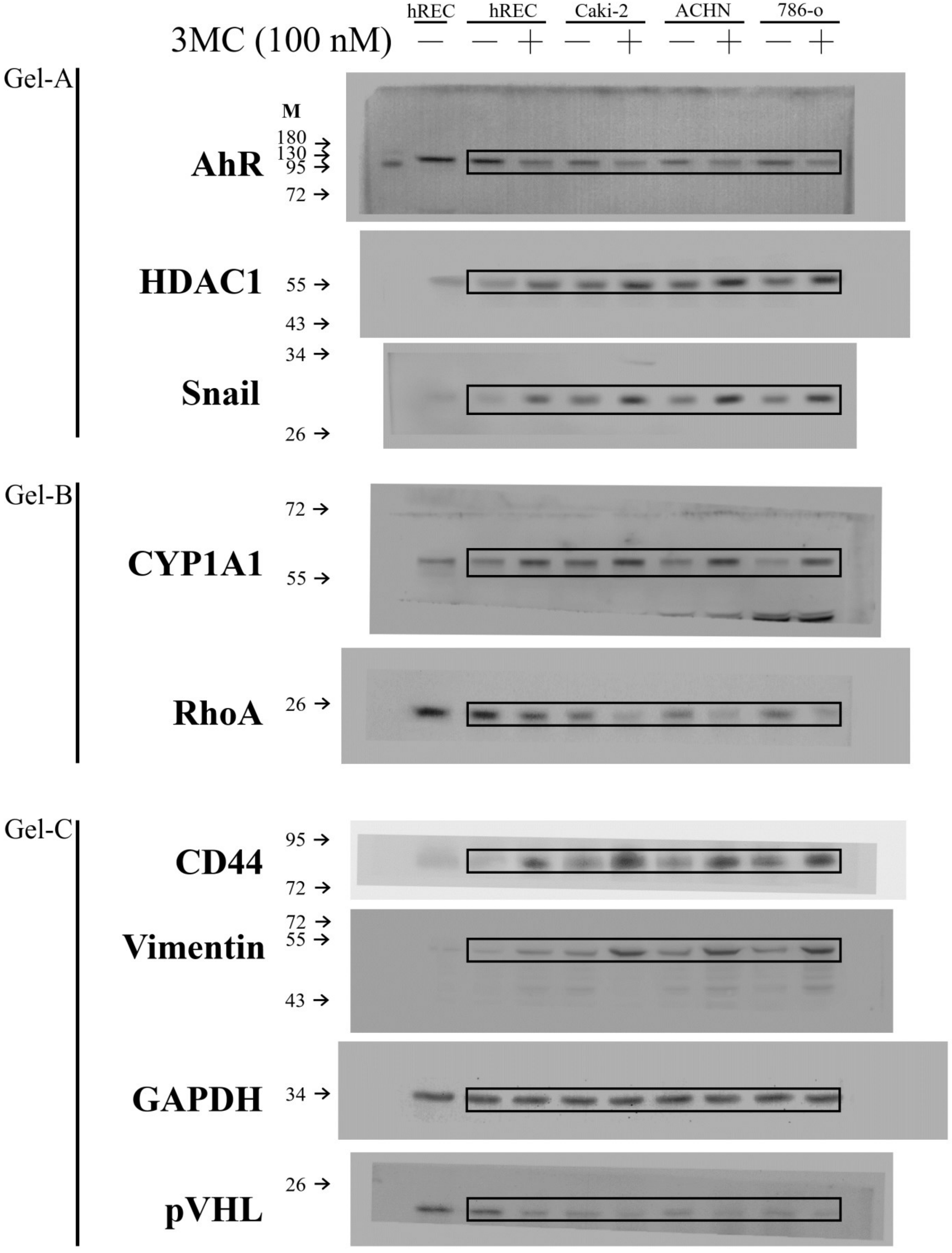

**Supple. Fig. 1(C)**

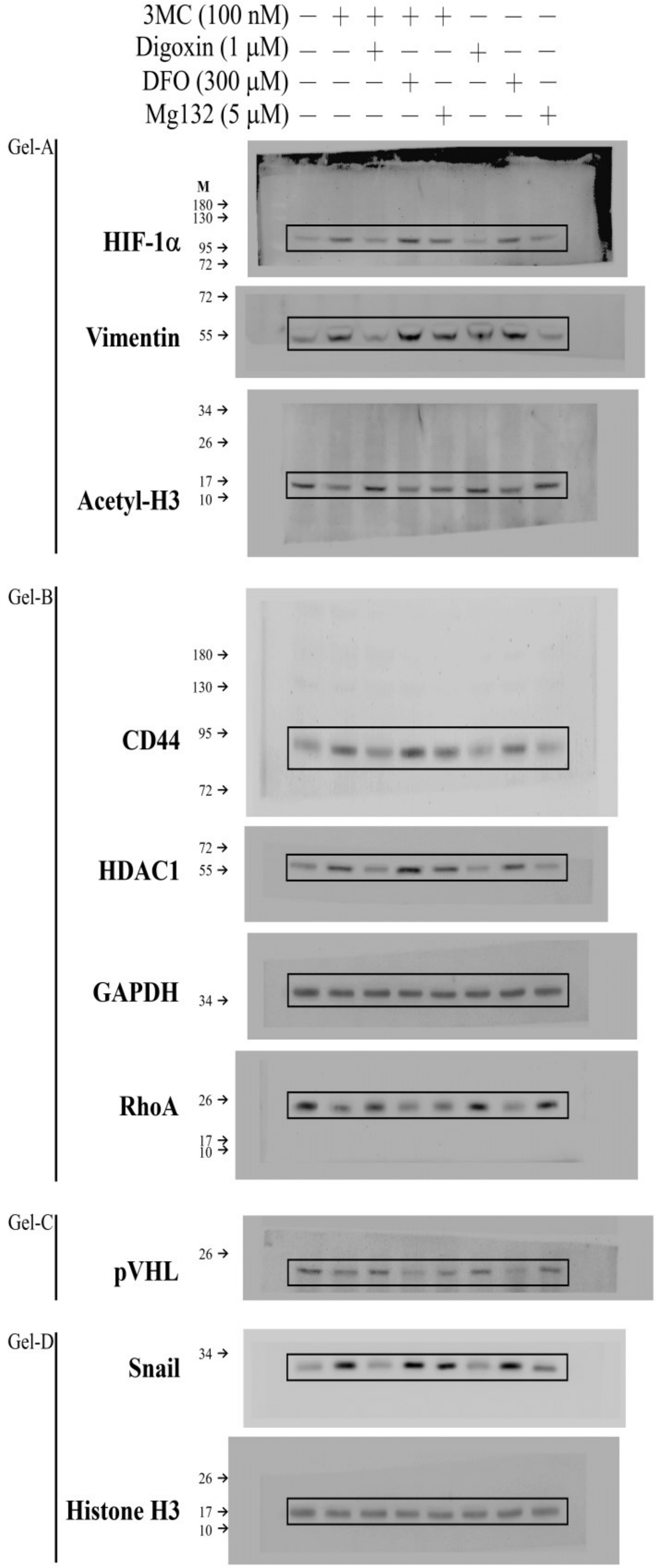


**Supple. Fig. 2(A)**

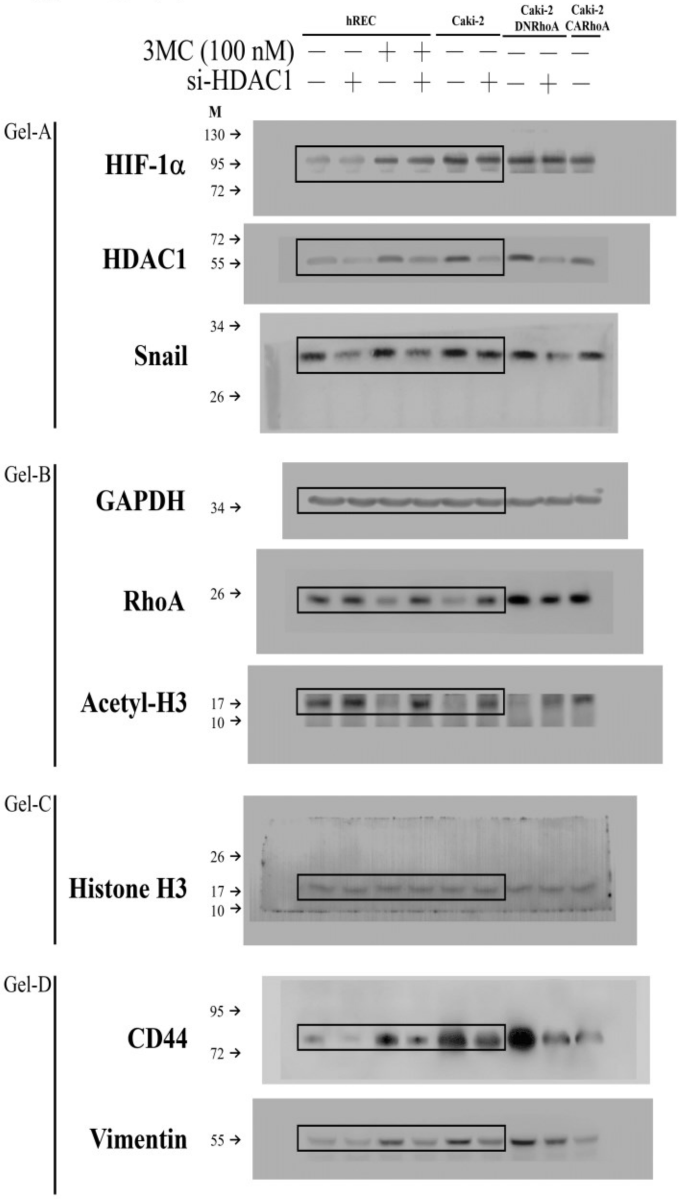

**Supple. Fig. 2(B)**

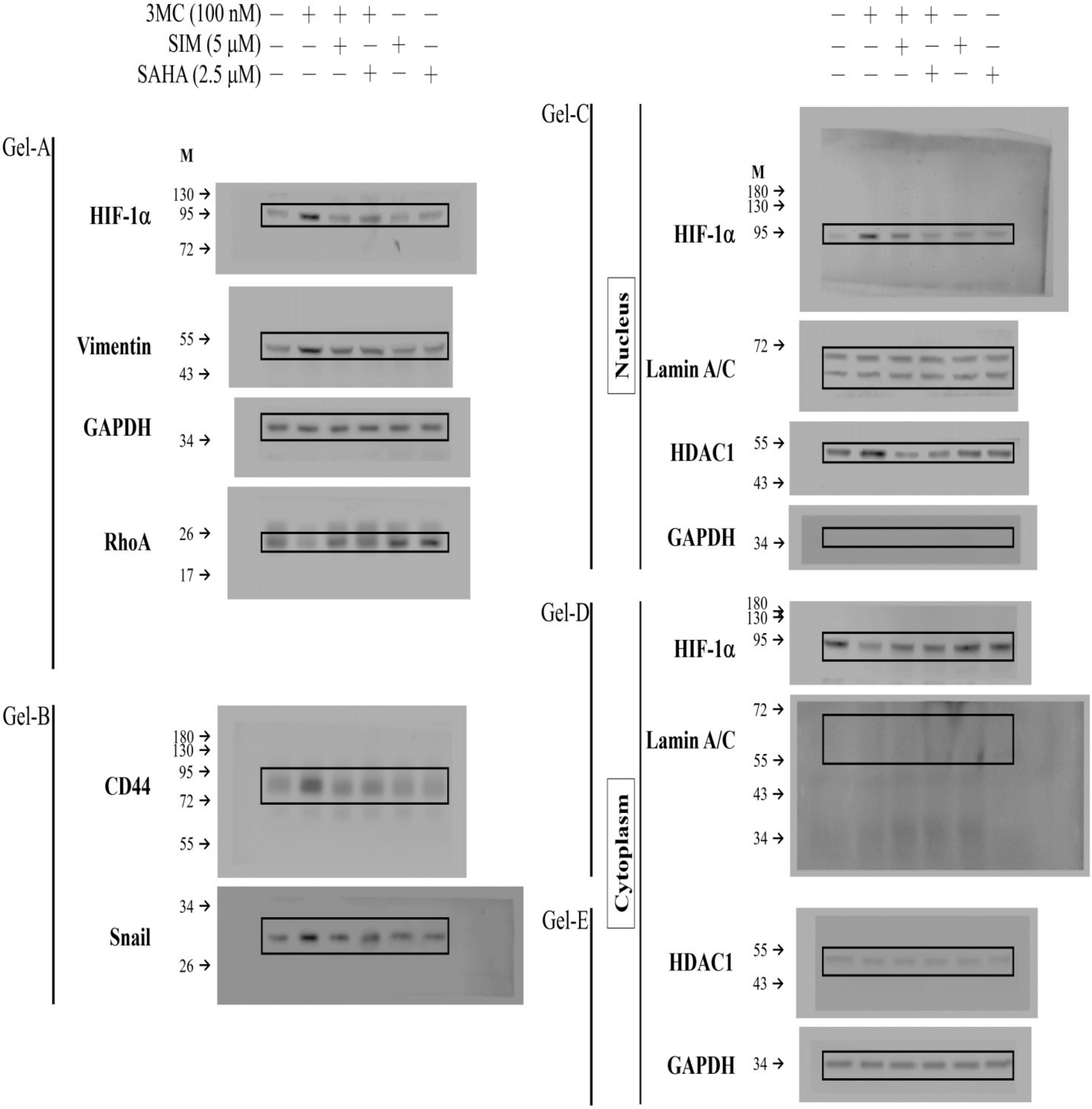

**Supple. Fig. 2(C)**

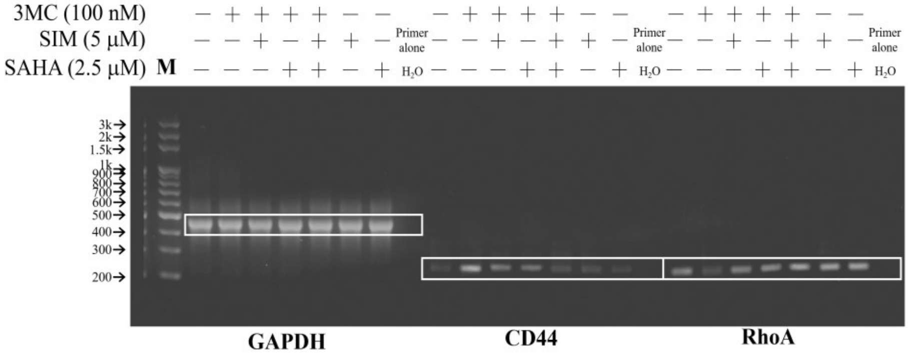


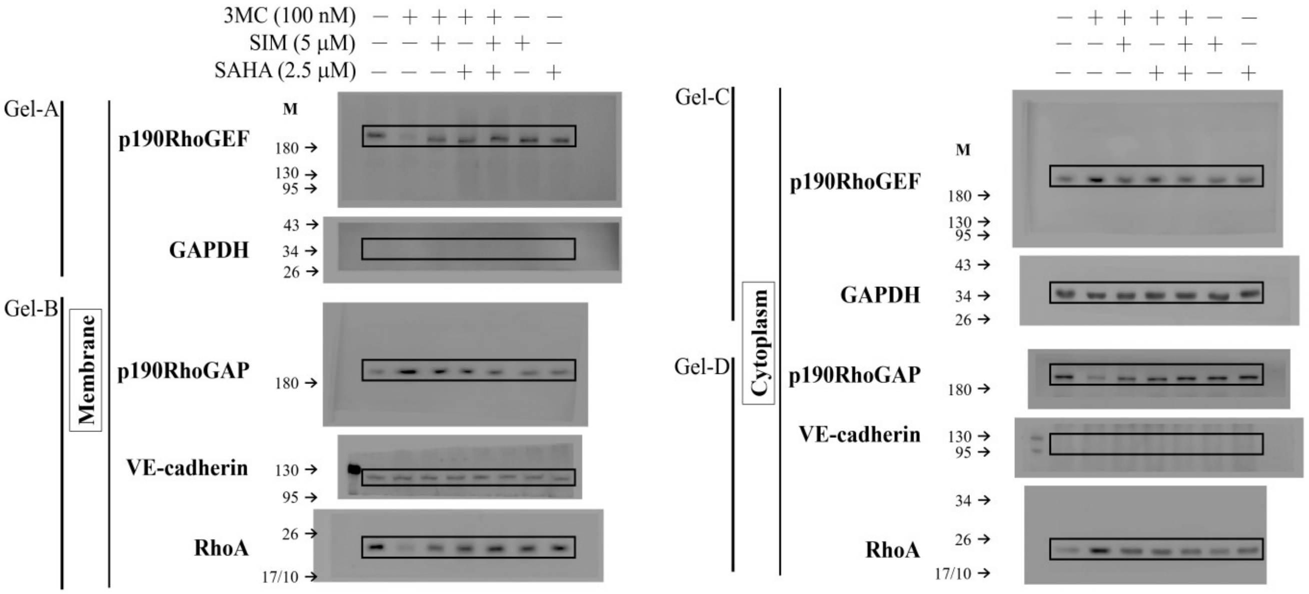

**Supple. Fig. 2(D)**

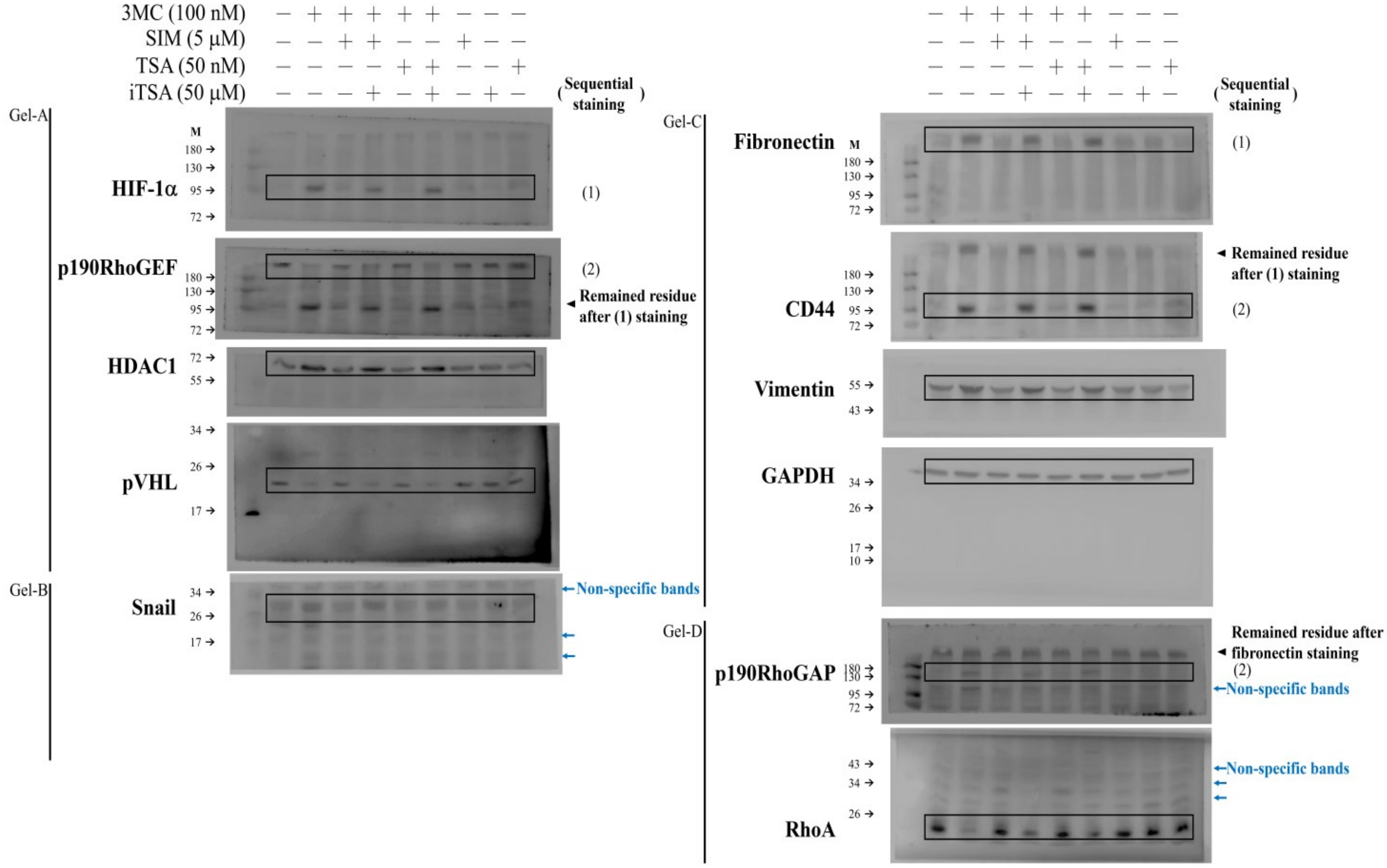

Supple. Fig. 2(E)

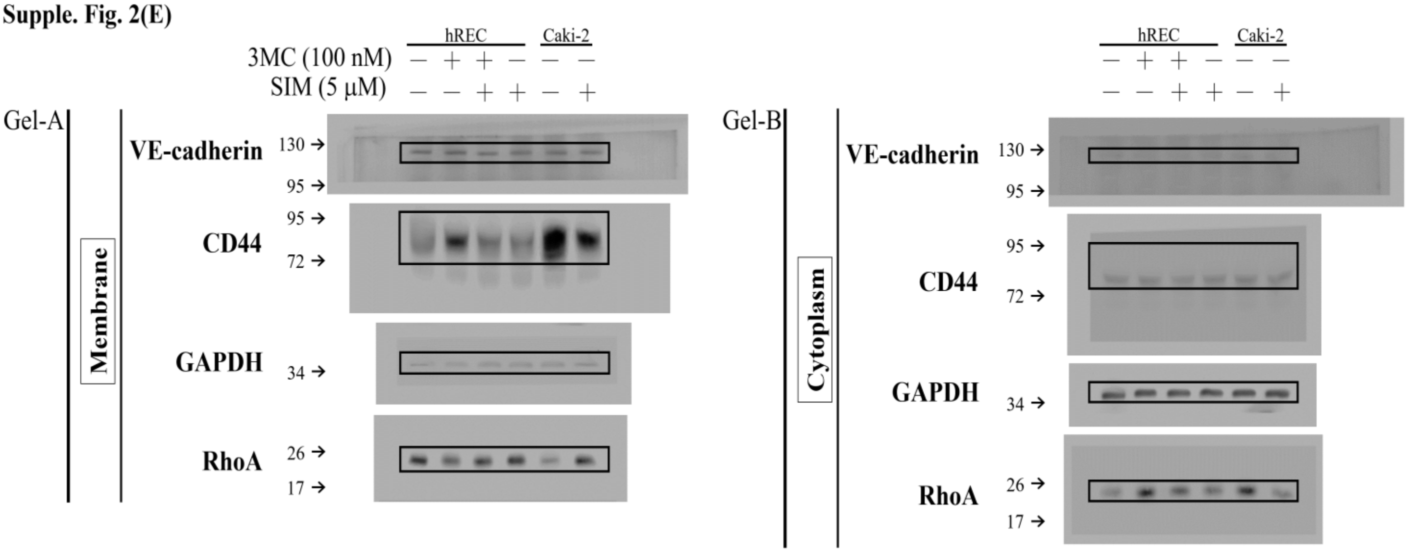

Supple. Fig. 3(A)

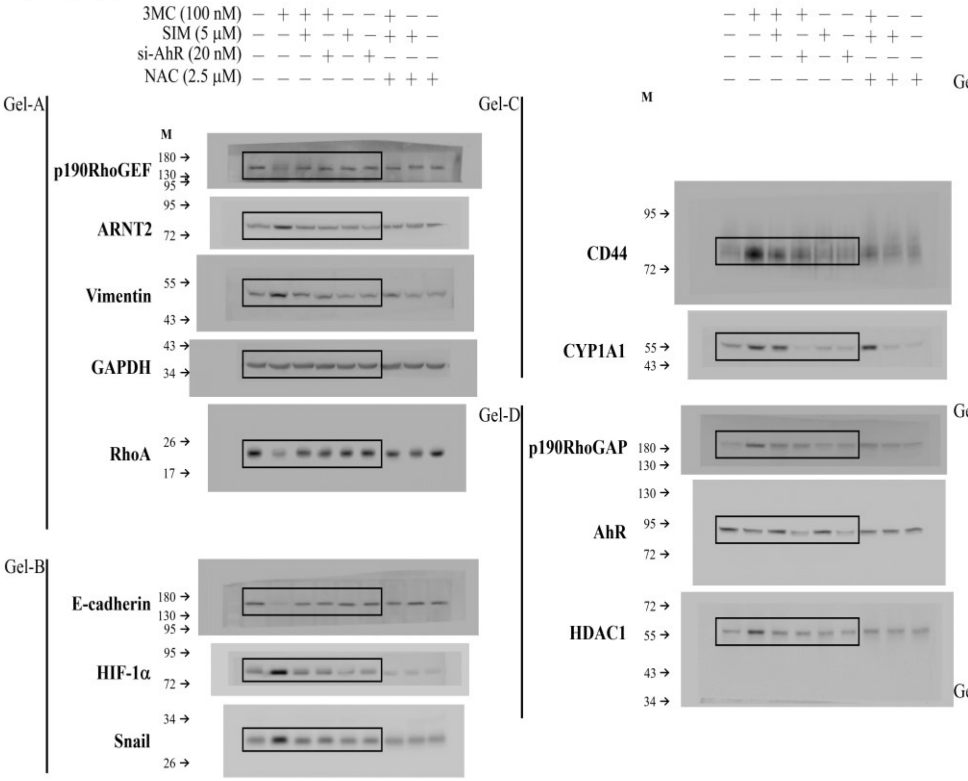


Supple. Fig. 3(C)

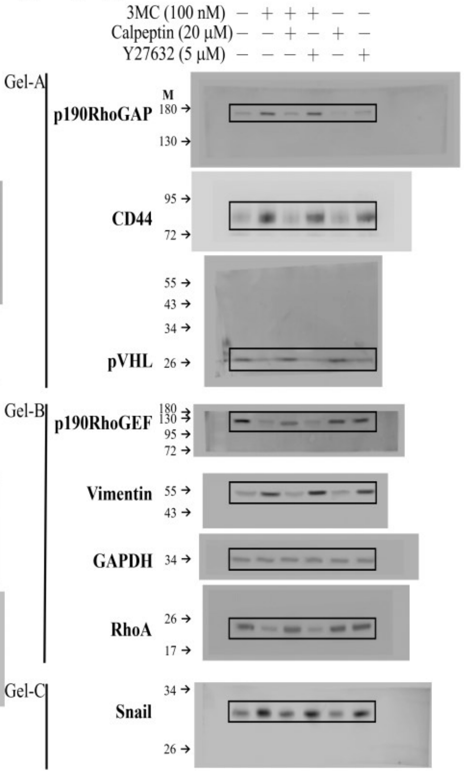

**Supple. Fig. 3(D)**

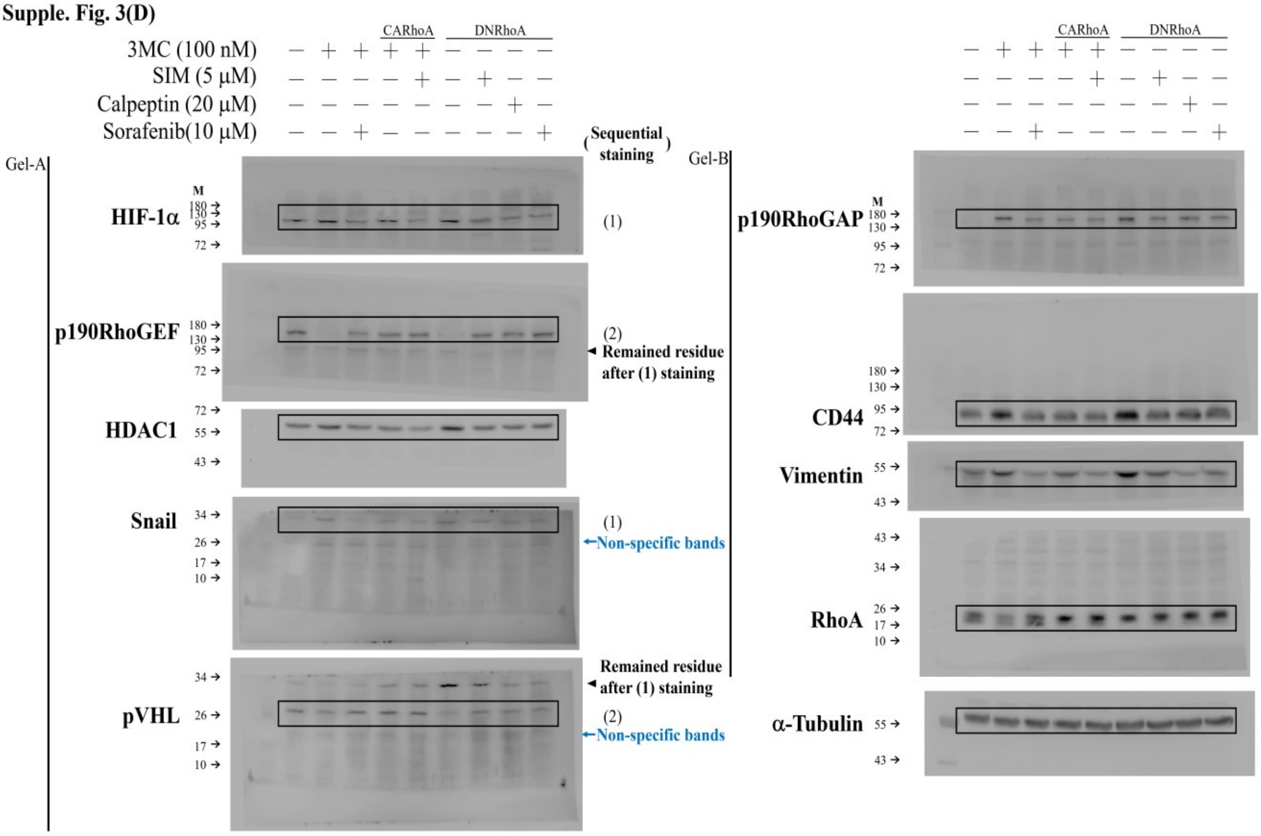

**Supple. Fig. 3(E)**

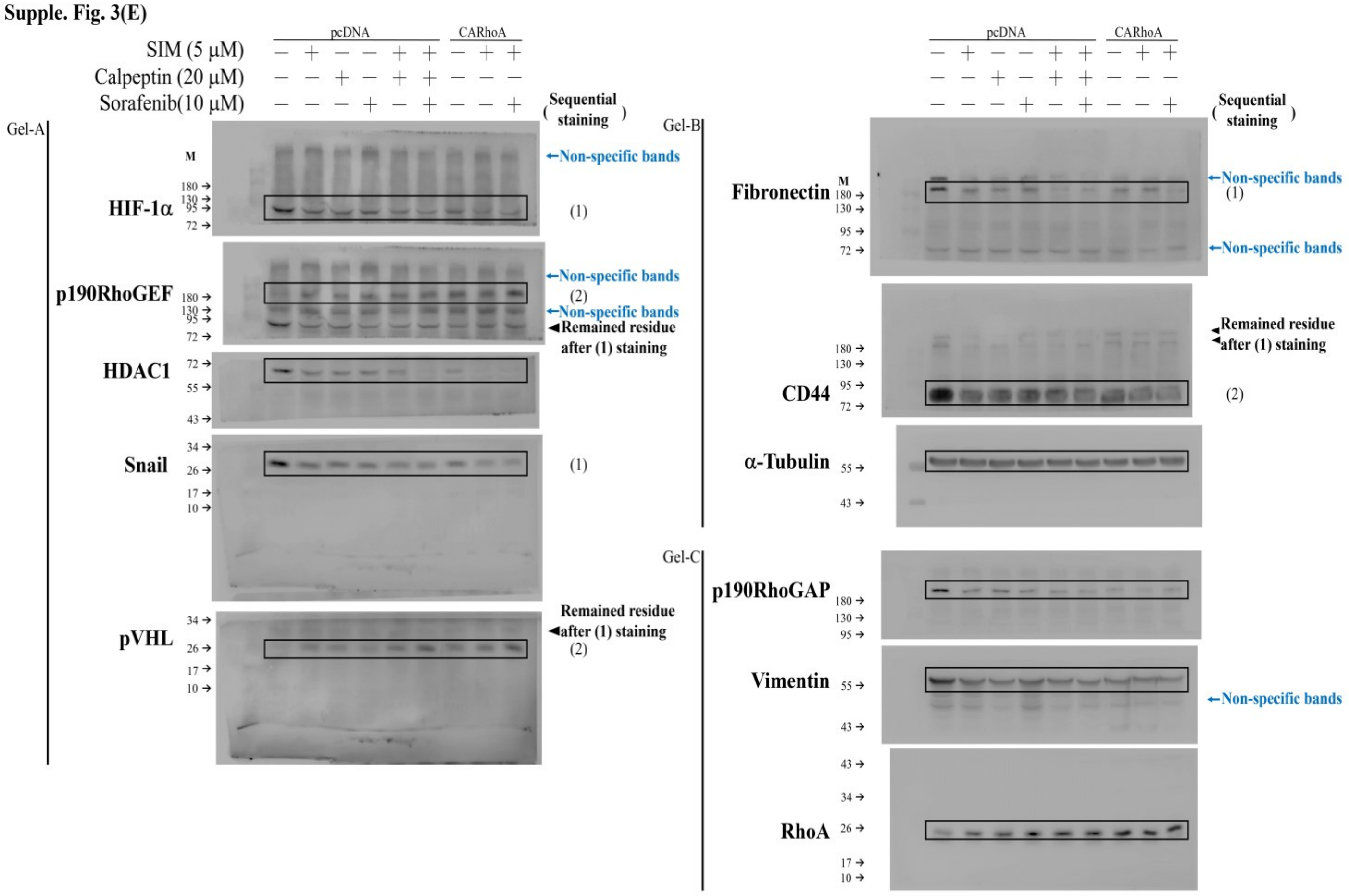

Supple. Fig. 4(A)

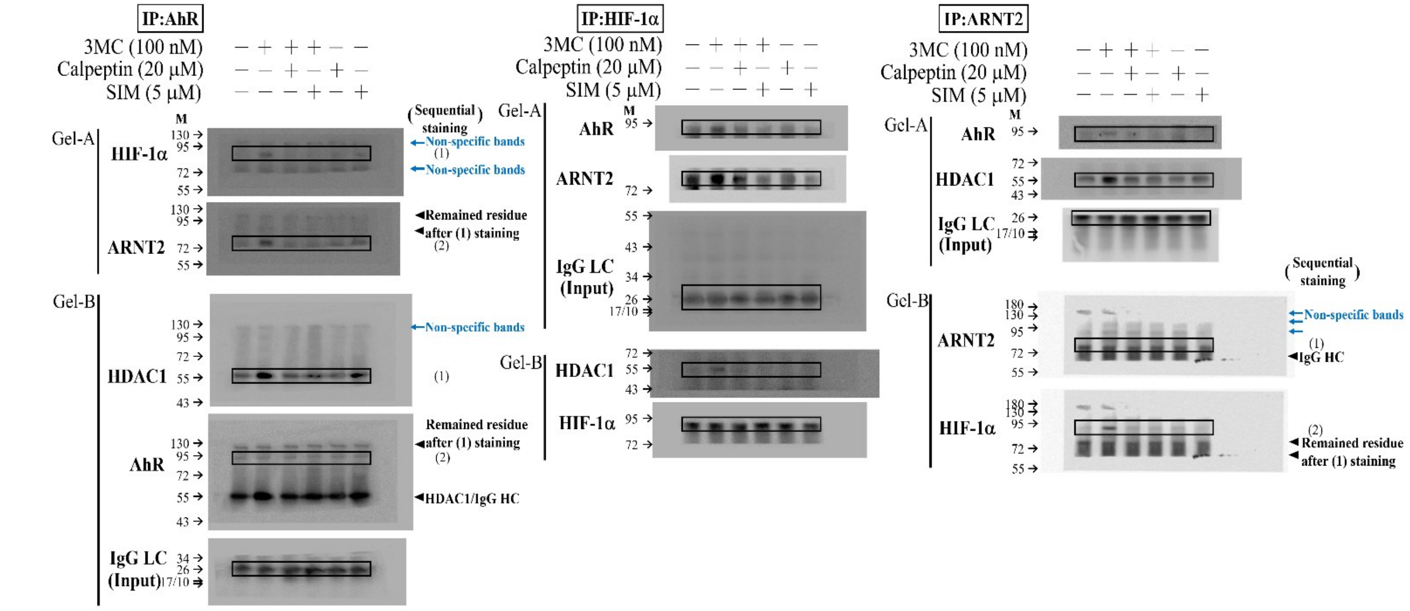


Supple. Fig. 4(B)

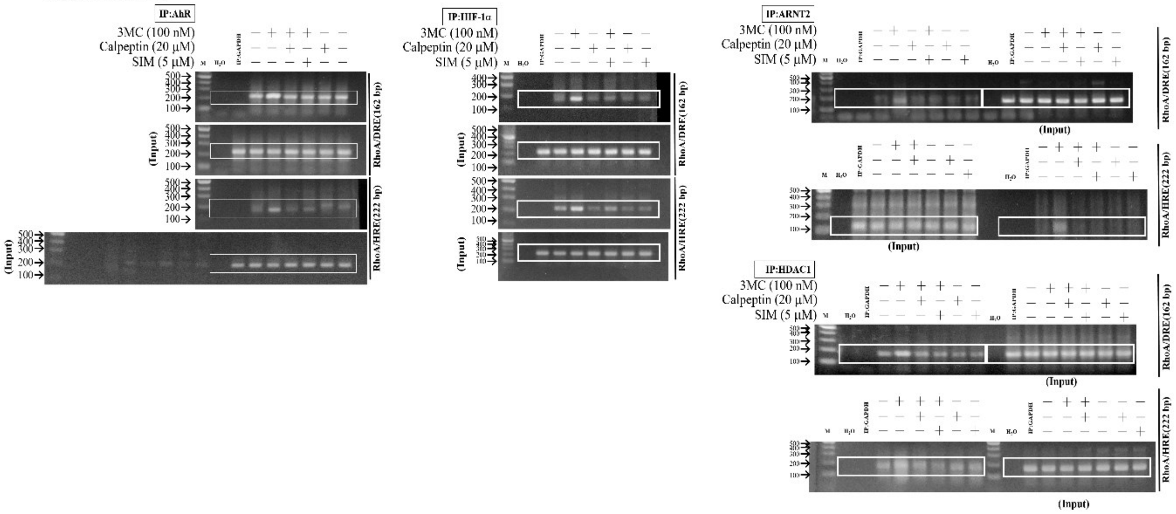

## Supple. Fig. 5(B)

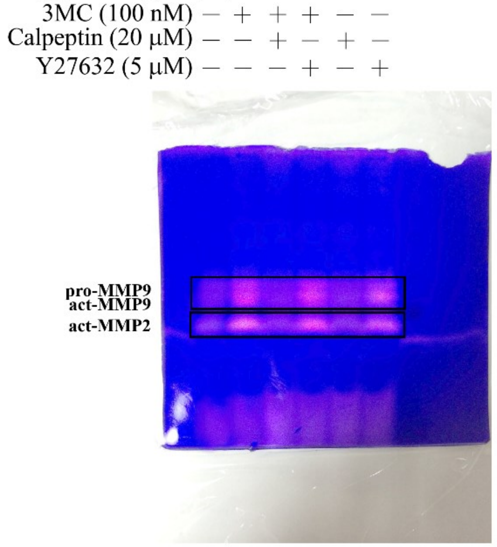


## Supple. Fig. 6(C)

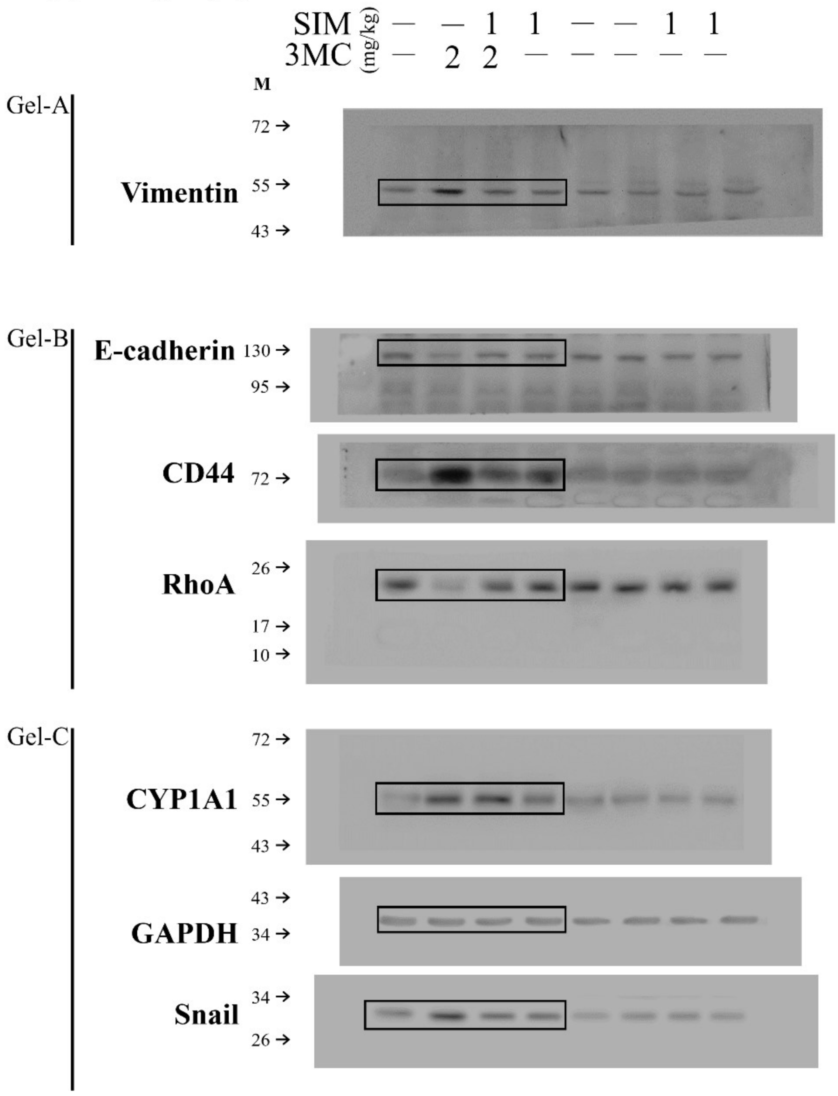

## Supple. Fig. S1

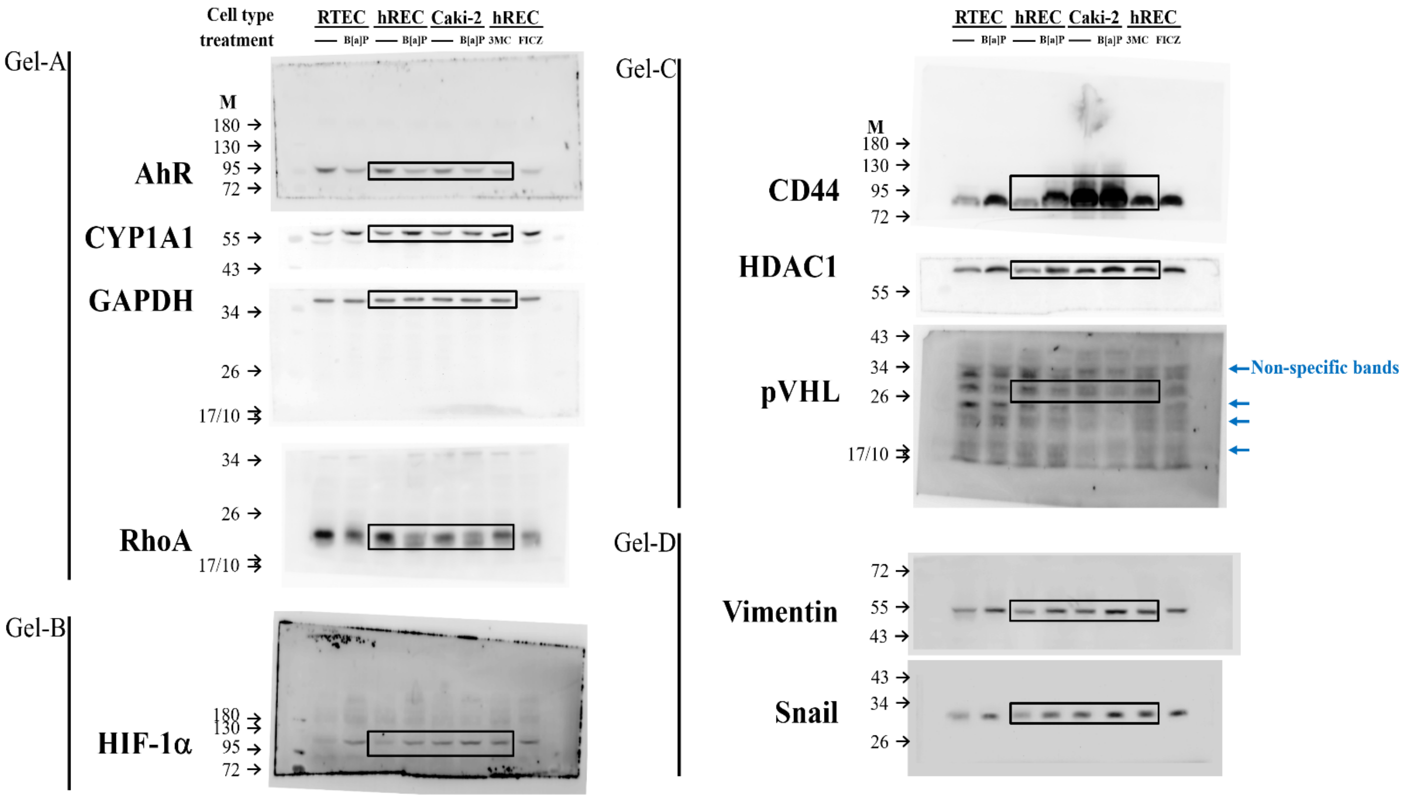

# Cell Line DNA Typing Report

Mission Biotech
10F-3, No.3, Yuanchi Street
Nangang, Taipei
Taiwan 115
Tel: 886 2 26557128
Email: service@missionbio.com.tw

Case Number:CID20170008

Report Date:01/25/2017

**Sample Information：**

i. Applicant Name:阮淑慧 Shu-Hui Juan
ii. Institution:臺北醫學大學醫學系生理學科 Department of Physiology, School of Medicine, Taipei Medical University
iii. Sample Description:caki-2
iv. Sample type: Cell Pellet
v. Sample Received Date:01/18/2017

**Allele table for the tested cell DNA**

| STR Locus | Repeat Numbers |
|---|---|
| D5S818 | 11,12 |
| D13S317 | 11,12 |
| D7S820 | 8,12 |
| D16S539 | 12 |
| vWA | 15,17 |
| TH01 | 6,8 |
| Amelogenin | X |
| TPOX | 8,11 |
| CSF1PO | 10,11 |
| D21S11 | 28,30 |

**Test Description：**

CaseNumber: CID20170008
Test Date: 01/23/2017

Sample was extracted by Roche MagNA Pure Compact System.
DNA conc.=75.9 ng/µl
OD260/280 = 1.97
OD260/230 = 1.97

This test was performed by using the PromegaGenePrint® 10 System and analyzed by ABI PRISM 3730 GENETIC ANALYZER and GeneMapper® Software V3.7.

*Verified by:*
*Laboratory Director(Title)*

Liang Kuei Chang

*01/25/2017*

# Allele Report

Case Number: CID20170008

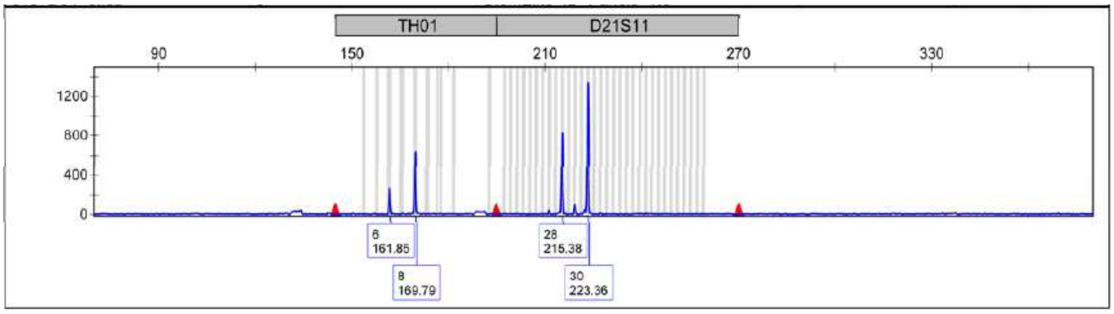


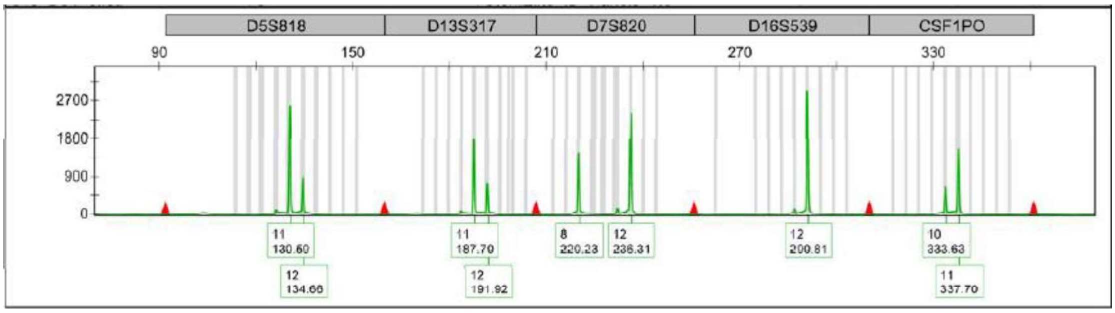


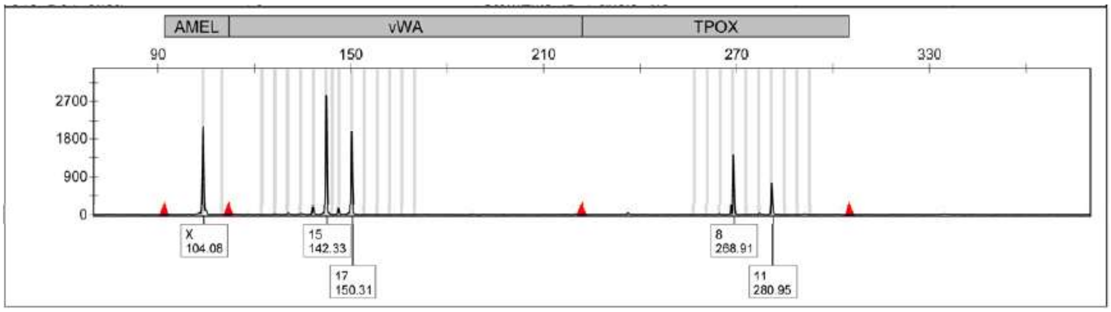


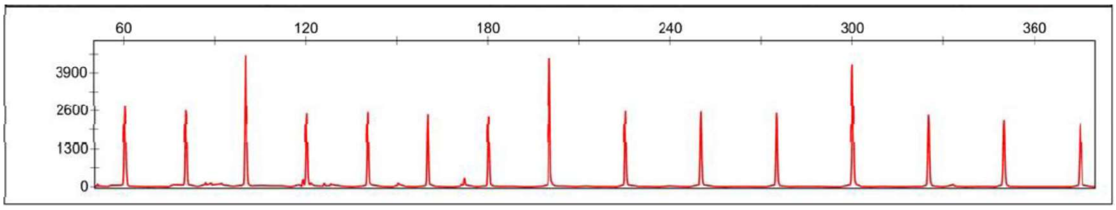

| | Dye | Sample File Name | Marker | Allele | Size | Height | Area |
|---|---|---|---|---|---|---|---|
| 1 | B,1 | 013_D04_caki-2.fsa | TH01 | 6 | 161.85 | 277 | 1591 |
| 2 | B,2 | 013_D04_caki-2.fsa | TH01 | 8 | 169.79 | 646 | 3861 |
| 3 | B,3 | 013_D04_caki-2.fsa | D21S11 | 28 | 215.38 | 835 | 5113 |
| 4 | B,4 | 013_D04_caki-2.fsa | D21S11 | 30 | 223.36 | 1347 | 8402 |
| 5 | G,1 | 013_D04_caki-2.fsa | D5S818 | 11 | 130.59 | 2591 | 16114 |
| 6 | G,2 | 013_D04_caki-2.fsa | D5S818 | 12 | 134.66 | 876 | 5451 |
| 7 | G,3 | 013_D04_caki-2.fsa | D13S317 | 11 | 187.7 | 1801 | 11085 |
| 8 | G,4 | 013_D04_caki-2.fsa | D13S317 | 12 | 191.92 | 742 | 4634 |
| 9 | G,5 | 013_D04_caki-2.fsa | D7S820 | 8 | 220.23 | 1482 | 9503 |
| 10 | G,6 | 013_D04_caki-2.fsa | D7S820 | 12 | 236.31 | 2399 | 15862 |
| 11 | G,7 | 013_D04_caki-2.fsa | D16S539 | 12 | 290.81 | 2931 | 19782 |
| 12 | G,8 | 013_D04_caki-2.fsa | CSF1PO | 10 | 333.63 | 674 | 4694 |
| 13 | G,9 | 013_D04_caki-2.fsa | CSF1PO | 11 | 337.7 | 1580 | 11126 |
| 14 | Y,1 | 013_D04_caki-2.fsa | AMEL | X | 104.08 | 2129 | 13118 |
| 15 | Y,2 | 013_D04_caki-2.fsa | vWA | 15 | 142.33 | 2854 | 18114 |
| 16 | Y,3 | 013_D04_caki-2.fsa | vWA | 17 | 150.31 | 1983 | 12547 |
| 17 | Y,4 | 013_D04_caki-2.fsa | TPOX | 8 | 268.91 | 1397 | 9262 |
| 18 | Y,5 | 013_D04_caki-2.fsa | TPOX | 11 | 280.95 | 774 | 5237 |

# Allelic Ladder

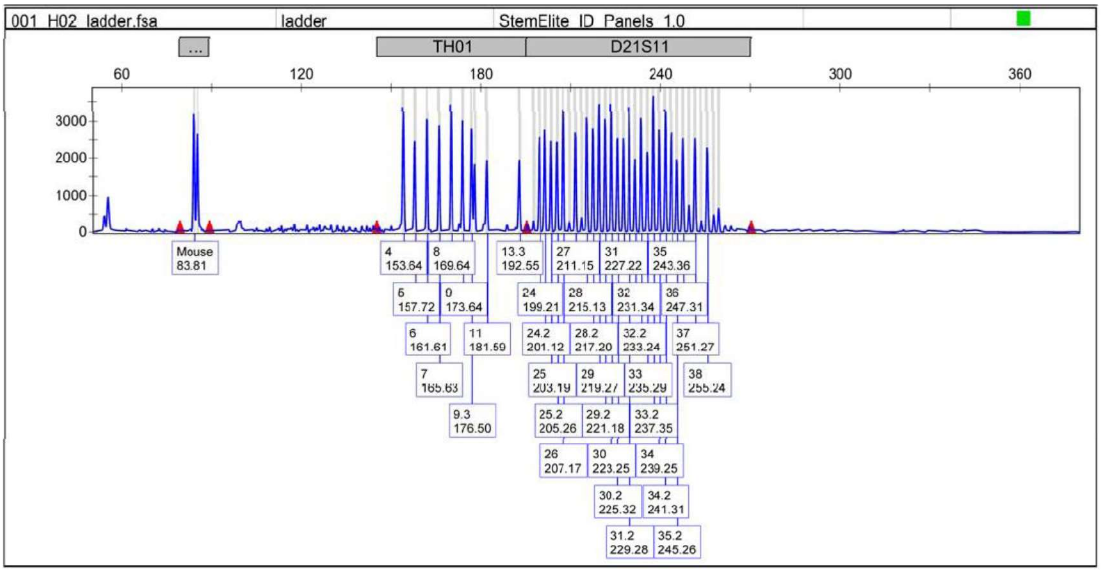


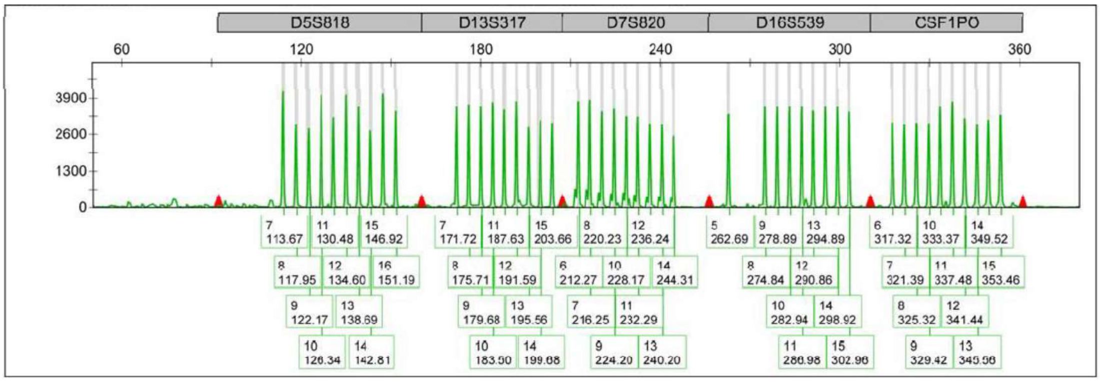


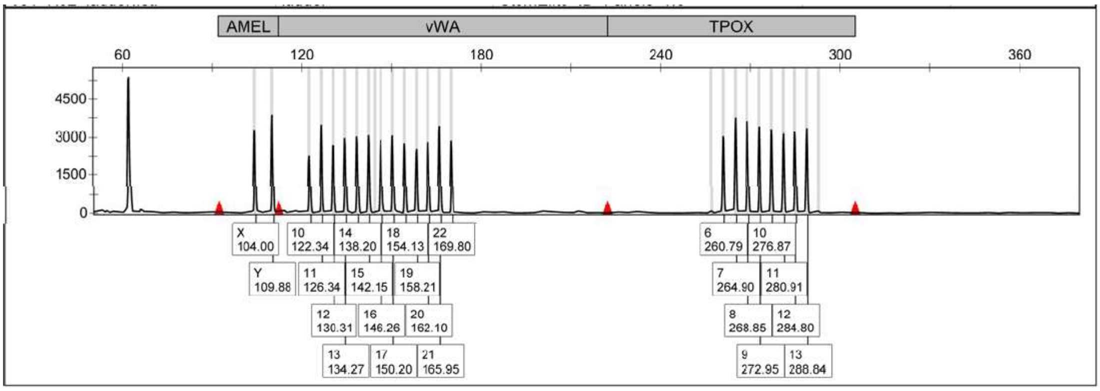


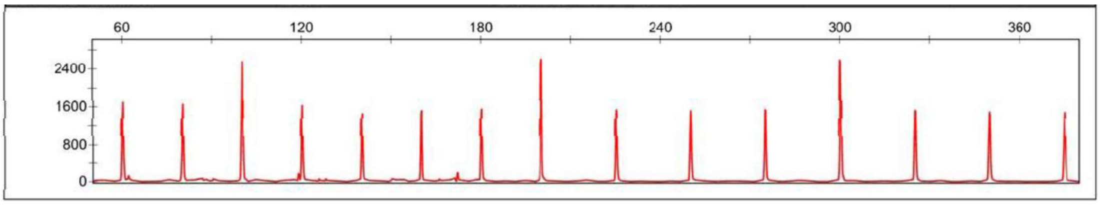

- *This STR analysis testing service is for research purposes only, andis not to be used for clinical diagnosis or applications involving humans.*
- *This test is just for typing the specific loci of the applied sample. The applicants may have to compare the results with the database of some bioresource institutes such as ATCC, JCRB or DSMZ by themselves.*

*Genelabs Life science* www.genelabs.com.tw
Phone:(02)26557678, (04)22633813, (06)2094380
Freecall:0800-231914,0800-094380

# Cell Line DNA Typing Report

Mission Biotech
10F-3, No.3, Yuanchi Street
Nangang, Taipei
Taiwan 115
Tel: 886 2 26557128
Email: service@missionbio.com.tw

Case Number:CID20170110
Report Date:06/20/2017

**Sample Information：**

i. Applicant Name:阮淑慧教授 Dr. Shu-Hui Juan
ii. Institution:臺北醫學大學醫學系生理學科 Department of Physiology, School of Medicine, Taipei Medical University
iii. Sample Description:ACHN
iv. Sample type: Cell Pellet
v. Sample Received Date:06/13/2017

**Allele table for the tested cell DNA**

| STR Locus | Repeat Numbers |
|---|---|
| D5S818 | 12 |
| D13S317 | 12 |
| D7S820 | 9,11 |
| D16S539 | 12,13 |
| vWA | 16,17 |
| TH01 | 8 |
| Amelogenin | X |
| TPOX | 8,11 |
| CSF1PO | 11 |
| D21S11 | 30 |

**Test Description：**

CaseNumber: CID20170110
Test Date: 06/15/2017

Sample was extracted by Roche MagNA Pure Compact System.
DNA conc.=__149.4__ng/µl
OD260/280 =__2.06__
OD260/230 =__2.31__

This test was performed by using the PromegaGenePrint® 10 System and analyzed by ABI PRISM 3730 GENETIC ANALYZER and GeneMapper® Software V3.7.

*Verified by:*
*Laboratory Director(Title)*

Liang Kuei Chang

*06/20/2017*

# Allele Report

Case Number: CID20170110

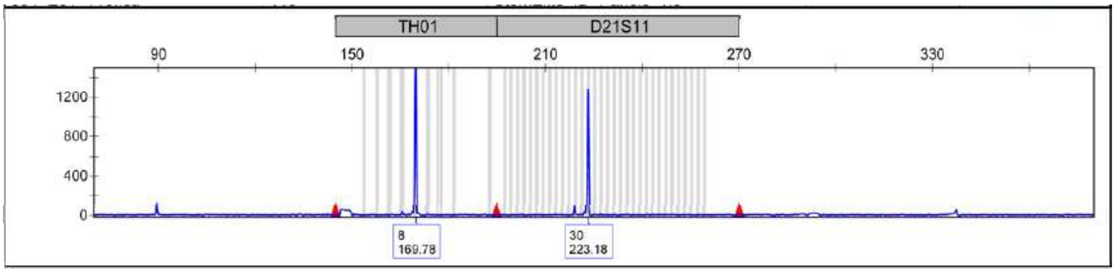


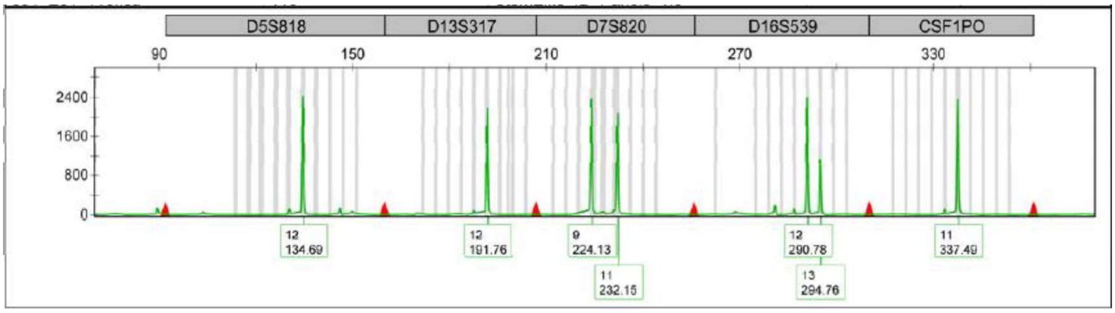


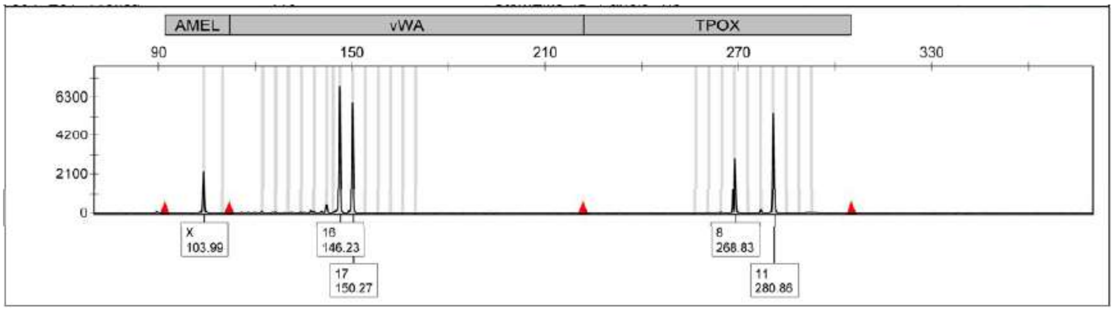


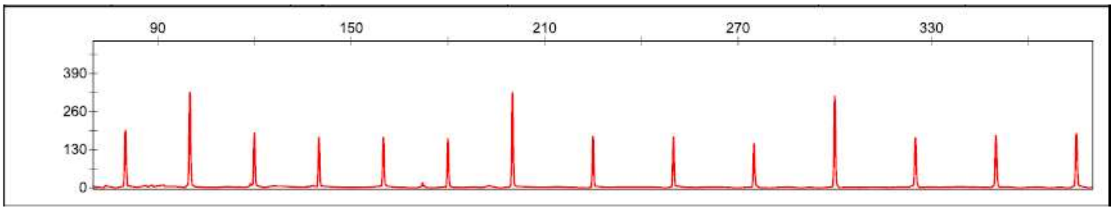

| | Dye | Sample File Name | Marker | Allele | Size | Height | Area |
|---|---|---|---|---|---|---|---|
| 1 | B,1 | 004_E01_ACHN.fsa | TH01 | 8 | 169.78 | 1526 | 9002 |
| 2 | B,2 | 004_E01_ACHN.fsa | D21S11 | 30 | 223.18 | 1290 | 7929 |
| 3 | G,1 | 004_E01_ACHN.fsa | D5S818 | 12 | 134.69 | 2431 | 14987 |
| 4 | G,2 | 004_E01_ACHN.fsa | D13S317 | 12 | 191.76 | 2185 | 13367 |
| 5 | G,3 | 004_E01_ACHN.fsa | D7S820 | 9 | 224.13 | 2371 | 15048 |
| 6 | G,4 | 004_E01_ACHN.fsa | D7S820 | 11 | 232.15 | 2070 | 13219 |
| 7 | G,5 | 004_E01_ACHN.fsa | D16S539 | 12 | 290.78 | 2409 | 16405 |
| 8 | G,6 | 004_E01_ACHN.fsa | D16S539 | 13 | 294.76 | 1130 | 7716 |
| 9 | G,7 | 004_E01_ACHN.fsa | CSF1PO | 11 | 337.49 | 2363 | 16844 |
| 10 | Y,1 | 004_E01_ACHN.fsa | AMEL | X | 103.99 | 2245 | 13612 |
| 11 | Y,2 | 004_E01_ACHN.fsa | vWA | 16 | 146.23 | 6889 | 43134 |
| 12 | Y,3 | 004_E01_ACHN.fsa | vWA | 17 | 150.27 | 6056 | 37317 |
| 13 | Y,4 | 004_E01_ACHN.fsa | TPOX | 8 | 268.83 | 2955 | 19268 |
| 14 | Y,5 | 004_E01_ACHN.fsa | TPOX | 11 | 280.86 | 5493 | 36397 |

# Allelic Ladder

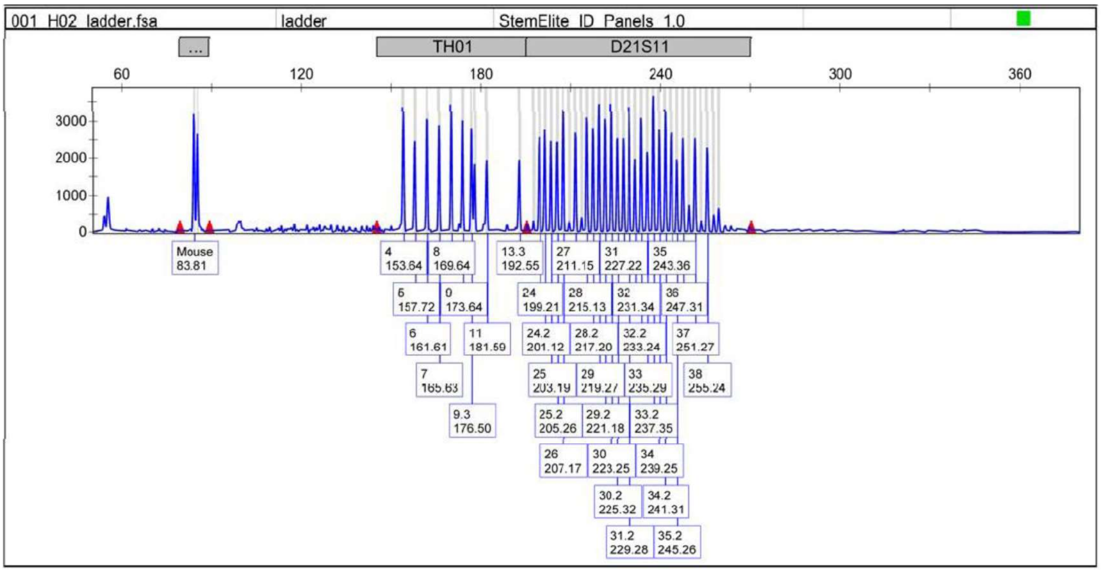


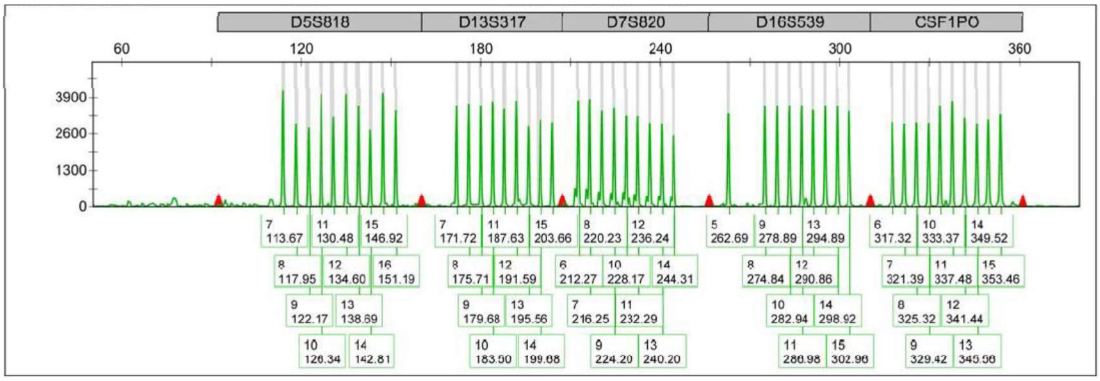


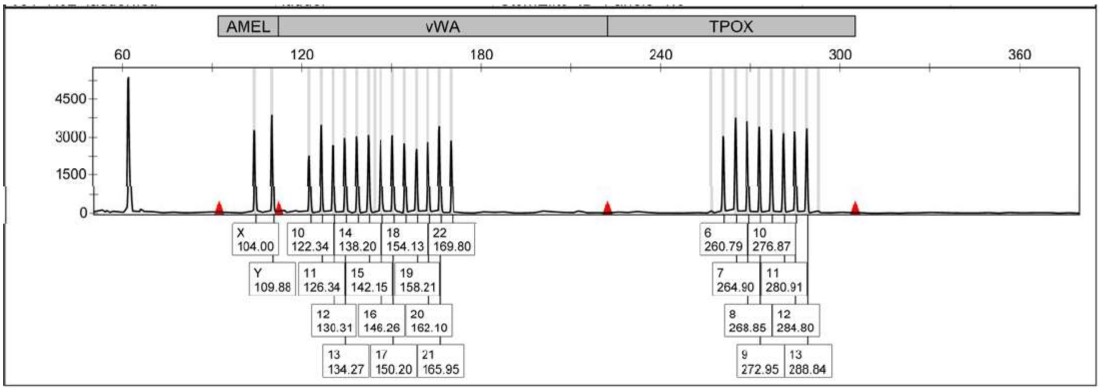


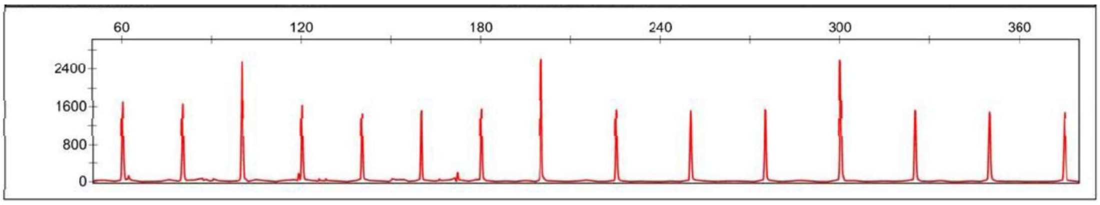

- *This STR analysis testing service is for research purposes only, andis not to be used for clinical diagnosis or applications involving humans.*
- *This test is just for typing the specific loci of the applied sample. The applicants may have to compare the results with the database of some bioresource institutes such as ATCC, JCRB or DSMZ by themselves.*

*Genelabs Life science* www.genelabs.com.tw
Phone:(02)26557678, (04)22633813, (06)2094380
Freecall:0800-231914,0800-094380

# Cell Line DNA Typing Report

Mission Biotech
10F-3, No.3, Yuanchi Street
Nangang, Taipei
Taiwan 115
Tel: 886 2 26557128
Email: service@missionbio.com.tw

Case Number:CID20170111
Report Date:06/20/2017

**Sample Information：**

i. Applicant Name:阮淑慧教授 Dr. Shu-Hui Juan
ii. Institution:臺北醫學大學醫學系生理學科 Department of Physiology, School of Medicine, Taipei Medical University
iii. Sample Description:786-0
iv. Sample type: Cell Pellet
v. Sample Received Date:06/13/2017

**Allele table for the tested cell DNA**

| STR Locus | Repeat Numbers |
|---|---|
| D5S818 | 9 |
| D13S317 | 8 |
| D7S820 | 11,12 |
| D16S539 | 12 |
| vWA | 15,17 |
| TH01 | 6,9.3 |
| Amelogenin | X,Y |
| TPOX | 8,11 |
| CSF1PO | 10 |
| D21S11 | 29,30 |

**Test Description：**

CaseNumber: CID20170111
Test Date: 06/15/2017

Sample was extracted by Roche MagNA Pure Compact System.
DNA conc.=148.0 ng/μl
OD260/280 =1.95
OD260/230 =1.18

This test was performed by using the PromegaGenePrint® 10 System and analyzed by ABI PRISM 3730 GENETIC ANALYZER and GeneMapper® Software V3.7.

*Verified by:*
*Laboratory Director(Title)*

Liang Kuei Chang

*06/20/2017*

# Allele Report

Case Number: CID20170111

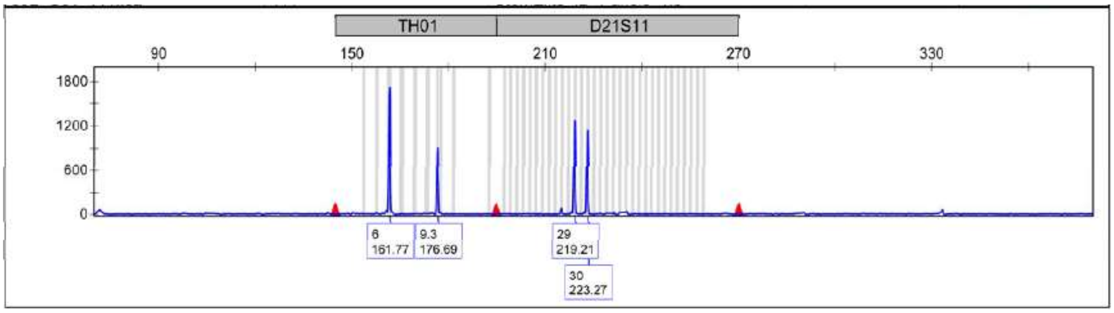


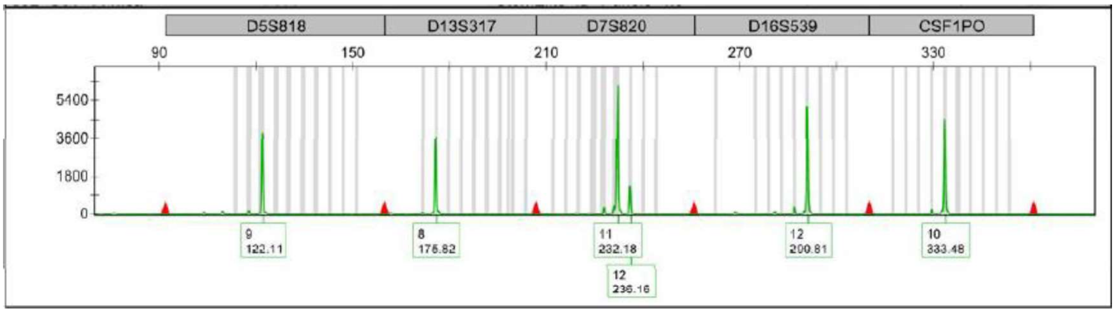


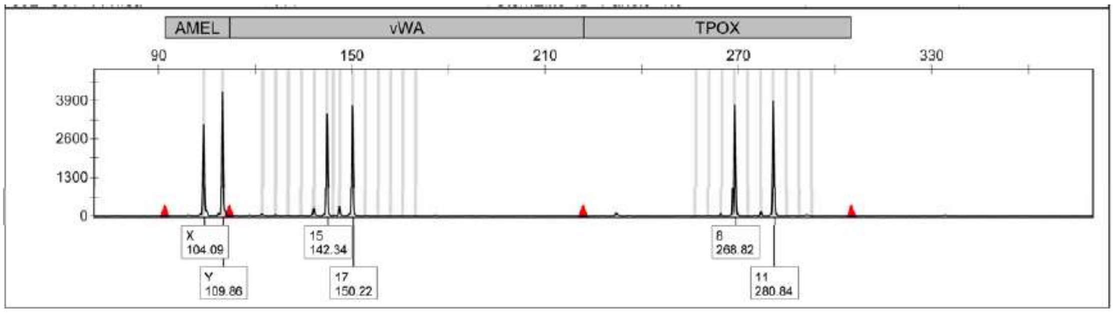


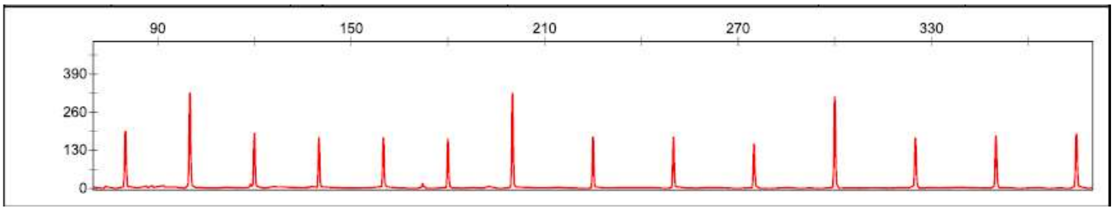

| | Dye | Sample File Name | Marker | Allele | Size | Height | Area |
|---|---|---|---|---|---|---|---|
| 1 | B,1 | 002_G01_786-0.fsa | TH01 | 6 | 161.77 | 1733 | 10224 |
| 2 | B,2 | 002_G01_786-0.fsa | TH01 | 9.3 | 176.69 | 898 | 5134 |
| 3 | B,3 | 002_G01_786-0.fsa | D21S11 | 29 | 219.21 | 1287 | 7864 |
| 4 | B,4 | 002_G01_786-0.fsa | D21S11 | 30 | 223.27 | 1141 | 6908 |
| 5 | G,1 | 002_G01_786-0.fsa | D5S818 | 9 | 122.11 | 3847 | 23782 |
| 6 | G,2 | 002_G01_786-0.fsa | D13S317 | 8 | 175.82 | 3651 | 21899 |
| 7 | G,3 | 002_G01_786-0.fsa | D7S820 | 11 | 232.18 | 6088 | 39051 |
| 8 | G,4 | 002_G01_786-0.fsa | D7S820 | 12 | 236.16 | 1350 | 8537 |
| 9 | G,5 | 002_G01_786-0.fsa | D16S539 | 12 | 290.81 | 5135 | 35141 |
| 10 | G,6 | 002_G01_786-0.fsa | CSF1PO | 10 | 333.48 | 4534 | 32365 |
| 11 | Y,1 | 002_G01_786-0.fsa | AMEL | X | 104.09 | 3119 | 19545 |
| 12 | Y,2 | 002_G01_786-0.fsa | AMEL | Y | 109.86 | 4238 | 25822 |
| 13 | Y,3 | 002_G01_786-0.fsa | vWA | 15 | 142.34 | 3469 | 21624 |
| 14 | Y,4 | 002_G01_786-0.fsa | vWA | 17 | 150.22 | 3757 | 23193 |
| 15 | Y,5 | 002_G01_786-0.fsa | TPOX | 8 | 268.82 | 3785 | 24597 |
| 16 | Y,6 | 002_G01_786-0.fsa | TPOX | 11 | 280.84 | 3894 | 25829 |

# Allelic Ladder

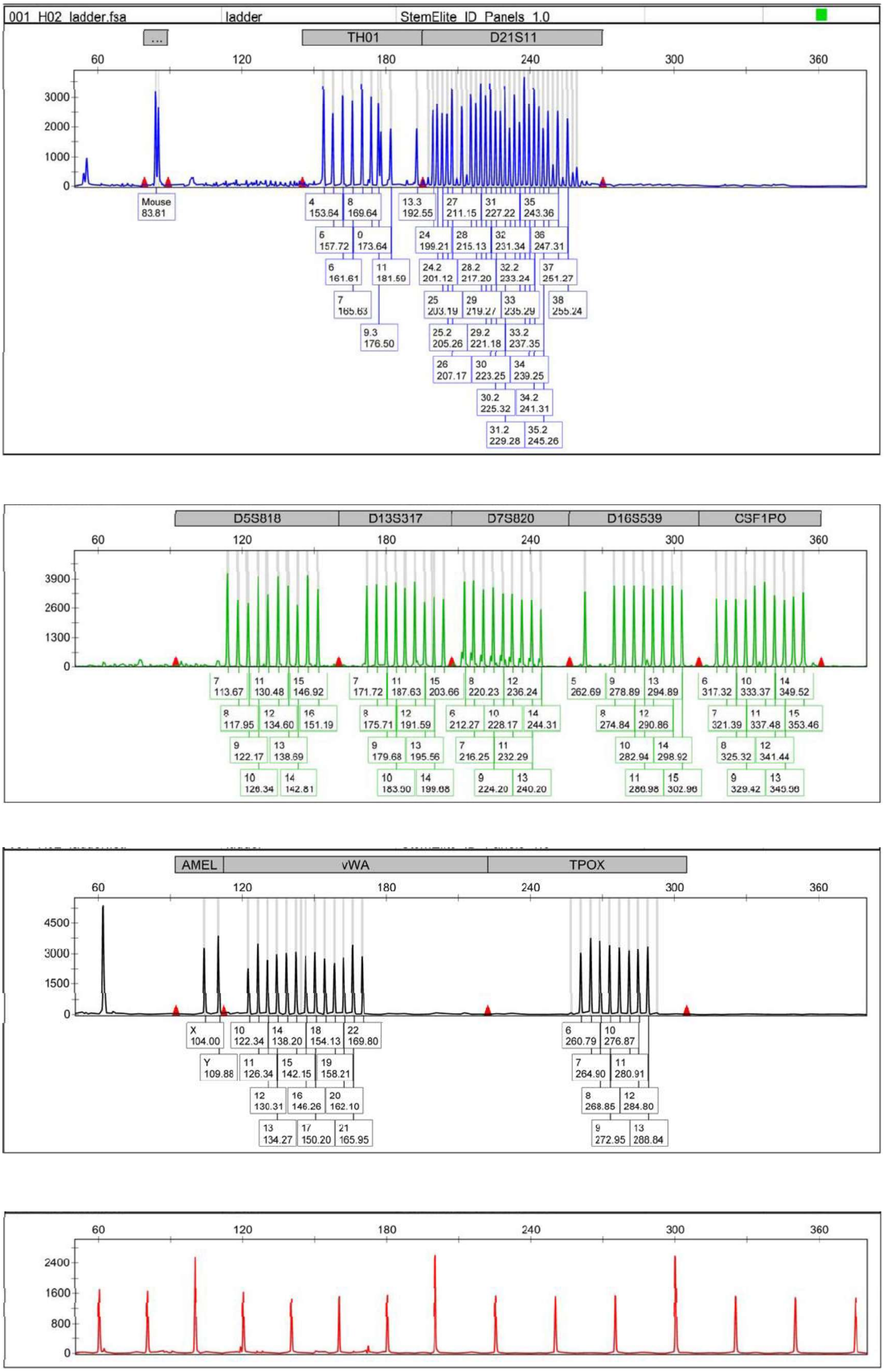

- *This STR analysis testing service is for research purposes only, andis not to be used for clinical diagnosis or applications involving humans.*
- *This test is just for typing the specific loci of the applied sample. The applicants may have to compare the results with the database of some bioresource institutes such as ATCC, JCRB or DSMZ by themselves.*

*Genelabs Life science* www.genelabs.com.tw
Phone:(02)26557678, (04)22633813, (06)2094380
Freecall:0800-231914,0800-094380
